# A novel method for estimating connectivity‐based parcellation of the human brain from diffusion MRI: Application to an aging cohort

**DOI:** 10.1002/hbm.25773

**Published:** 2022-03-11

**Authors:** Ana Coelho, Ricardo Magalhães, Pedro S. Moreira, Liliana Amorim, Carlos Portugal‐Nunes, Teresa Castanho, Nadine Correia Santos, Nuno Sousa, Henrique M. Fernandes

**Affiliations:** ^1^ Life and Health Sciences Research Institute (ICVS), School of Medicine University of Minho Braga Portugal; ^2^ ICVS/3B's—PT Government Associate Laboratory Braga/Guimarães Portugal; ^3^ Clinical Academic Center – Braga Braga Portugal; ^4^ Center for Music in the Brain (MIB) Aarhus University Aarhus Denmark; ^5^ Department of Psychiatry University of Oxford Oxford UK

**Keywords:** aging, brain parcellation, clustering, diffusion MRI, network neuroscience, structural connectivity

## Abstract

Connectivity‐based parcellation (CBP) methods are used to define homogenous and biologically meaningful parcels or nodes—the foundations of brain network fingerprinting—by grouping voxels with similar patterns of brain connectivity. However, we still lack a gold standard method and the use of CBPs to study the aging brain remains scarce. Our study proposes a novel CBP method from diffusion MRI data and shows its potential to produce a more accurate characterization of the longitudinal alterations in brain network topology occurring in aging. For this, we constructed whole‐brain connectivity maps from diffusion MRI data of two datasets: an aging cohort evaluated at two timepoints (mean interval time: 52.8 ± 7.24 months) and a normative adult cohort—MGH‐HCP. State‐of‐the‐art clustering techniques were used to identify the best performing technique. Furthermore, we developed a new metric (connectivity homogeneity fingerprint [CHF]) to evaluate the success of the final CBP in improving regional/global structural connectivity homogeneity. Our results show that our method successfully generates highly homogeneous parcels, as described by the significantly larger CHF score of the resulting parcellation, when compared to the original. Additionally, we demonstrated that the developed parcellation provides a robust anatomical framework to assess longitudinal changes in the aging brain. Our results reveal that aging is characterized by a reorganization of the brain's structural network involving the decrease of intra‐hemispheric, increase of inter‐hemispheric connectivity, and topological rearrangement. Overall, this study proposes a new methodology to perform accurate and robust evaluations of CBP of the human brain.

## 1 INTRODUCTION

Human brain organization is ruled by two main functional principles: integration and segregation. Functional integration is characterized by long‐range connections and functional segregation through local differentiation (Tononi, Sporns, & Edelman, 1994). Each functionally specialized brain region might be described by a different set of connections, so the two concepts of functional integration and segregation are not mutually exclusive, but instead they are closely entangled (Eickhoff, Thirion, Varoquaux, & Bzdok, 2015; Eickhoff, Yeo, & Genon, 2018). This view inspired the development of a new family of methods in neuroimaging research known as connectivity‐based parcellation (CBP; Eickhoff et al., 2015). CBP exploits the heterogeneity of connections within a brain region and divides it according to its voxels' connectivity profiles (Eickhoff et al., 2015; Reuter et al., 2020). After estimating the connectivity profiles (i.e., connection strengths between a seed voxel and a set of target voxels) of each voxel inside a region, voxels with similar connectivity profiles are grouped together. This is usually performed using clustering algorithms (e.g., k‐means clustering, hierarchical clustering, spectral clustering) and results in subregions which represent homogeneous units with regard to the measured connectivity. Connectivity between voxels can be defined as functional connectivity which is estimated from resting‐state functional magnetic resonance imaging (rs‐fMRI), structural connectivity which is derived from diffusion weighted imaging (DWI), or task‐dependent functional connectivity which is computed from meta‐analytic connectivity modeling (MACM).

Network analysis tools allow the characterization of brain's structural and functional organization through quantifiable topological properties, based on the concept that the brain is a complex network of interconnected regions (Bullmore & Sporns, 2009). In this sense, the brain network is modeled as a graph composed of nodes and edges. While edges, defined as either functional or structural connectivity, were already subject of many studies in recent years, nodes are most of the time defined arbitrarily (Tittgemeyer, Rigoux, & Knosche, 2018). The most common approach is to use a pre‐existing parcellation that divides the brain into different regions based in local properties, such as cytoarchitecture (Brodmann, 1985), myelo‐architecture (Vogt & Vogt, 1919), or receptor‐architecture (Zilles et al., 2002). Early efforts to define brain nodes using these local criteria usually required post‐mortem tissues or invasive studies and were extremely time consuming (Gao et al., 2018). As an alternative, local properties can be estimated using measurements derived from MRI, such as myelin density maps, but these will only reflect an indirect measure since these properties are not directly observable through MRI (Eickhoff et al., 2018). Furthermore, and although these parcellations define nodes with a biological meaning, they might not adequately reflect brain organization and inter‐individual variability, as connectivity also plays a role in brain differentiation (Arslan et al., 2018; Eickhoff et al., 2015). In contrast, nodes generated with CBP present high homogeneity and functional coherence and distinct connectivity patterns between them, making them suitable for network analysis (Arslan et al., 2018).

First studies performing CBP segmented only a single region of the brain. Examples include the thalamus (Behrens et al., 2003), medial frontal cortex (Johansen‐Berg et al., 2004), and Broca's area (Anwander, Tittgemeyer, von Cramon, Friederici, & Knosche, 2007). With the advent of new computational tools, whole‐brain approaches are becoming popular, yielding a great heterogeneity of methods (Eickhoff et al., 2018). However, to date, a robust and standard method to perform whole‐brain CBP is still missing. Moreover, the application of CBP methods to study the aging brain is very scarce and limited to specific brain regions (Fritz et al., 2019). Herein we propose a new method to create a CBP of the human brain using diffusion MRI data. For this, we implemented and tested different state‐of‐the‐art clustering techniques and selected the best performing according to different criteria (Silhouette scores and consistency of clusters' sizes). Additionally, we developed a new metric (connectivity homogeneity fingerprint [CHF]) to evaluate the final CBP and prove its possibly advantage over the original parcellation. This metric reflects if the voxels inside a region establish more homogeneous connections (i.e., if they are connected to the same parts of the brain) or more heterogeneous connections (i.e., if they are connected to different parts of the brain) and thus it demonstrates if the main goal of CBP was accomplished. We hypothesized that the generated CBP would present higher values of CHF in comparison to the original partition. Moreover, with the developed CBP, we characterized longitudinal changes in topological features of white matter structural connectivity networks during normal aging. To the best of our knowledge, this is the first study applying CBP methods to study age‐related longitudinal changes in the whole brain and we hypothesized that our method would be suitable to explore white matter structural connectivity changes during aging.

## 2 METHODS

### 2.1 Ethics statement

The present study was conducted in accordance with the principles expressed in the Declaration of Helsinki and was approved by the national ethical committee (Comissão Nacional de Proteção de Dados) and by the local ethics review boards (Hospital de Braga, Braga; Centro Hospitalar do Alto Ave, Guimarães and Unidade Local de Saúde do Alto Minho, Viana do Castelo/Ponte de Lima). The study goals and procedures were explained to the participants and all gave informed written consent.

### 2.2 Participants

The participants included in this study are part of a larger sample recruited for the SWITCHBOX Consortium project (www.switchbox-online.eu/), and are representative of the general Portuguese population with respect to age, gender, and education (Costa, Santos, Cunha, Palha, & Sousa, 2013; Santos et al., 2013, 2014). Primary exclusion criteria were inability to understand the informed consent, participant choice to withdraw from the study, incapacity and/or inability to attend MRI sessions, dementia and/or diagnosed neuropsychiatric, and/or neurodegenerative disorder (from medical records). Mini Mental State Examination (MMSE) scores below the adjusted thresholds for cognitive impairment were also used as exclusion criteria. The adjusted thresholds were the following: MMSE score <17 if individual with ≤4 years of formal school education and/or ≥72 years of age, and MMSE score <23 otherwise (follows the MMSE validation study for the Portuguese population; Guerreiro et al., 1994). These exclusion criteria were applied at both evaluations. Subjects were evaluated at two timepoints, with a mean interval time between first and last assessments of 52.8 months (*SD* = 7.24). At each evaluation, participants underwent an imaging session and a battery of neurocognitive/neuropsychological tests.

In the first assessment, 100 subjects were contacted for MRI screening. In the last assessment, 55 subjects accepted to participate and underwent MRI acquisition protocol, but one did not finish the diffusion acquisition. From these, one subject did not finish the diffusion acquisition and four subjects had brain lesions/pathology. A total of 51 individuals with data from both the first and last evaluations met all the inclusion criteria for this study.

Furthermore, in order to evaluate the robustness of the developed CBP method, we included a dataset from the Human Connectome Project (HCP). Specifically, 32 healthy adult participants of the Massachusetts General Hospital‐Human Connectome Project (MGH‐HCP) diffusion dataset were used (Fan et al., 2016). This dataset was acquired with the Siemens 3T Connectom scanner that has the capacity to produce a magnetic field gradient of up to 300 mT/m strength, which provides diffusion MRI data with high angular and spatial resolution and with ultra‐high *b* values up to 10,000 s/mm^2^ (Fan et al., 2016).

### 2.3 MRI data acquisition

All MRI assessments of the SWITCHBOX dataset were performed at Hospital de Braga (Braga, Portugal) on a clinical approved Siemens Magnetom Avanto 1.5T MRI scanner (Siemens Medical Solutions, Erlangen, Germany) with a 12‐channel receive‐only head‐coil. The imaging protocol included several different acquisitions. For the present study, two types of acquisition were considered: diffusion weighted imaging (DWI) and structural scans. For the DWI acquisition, a spin‐echo echo‐planar imaging (SE‐EPI) sequence was acquired with the following parameters: TR = 8,800 ms, TE = 99 ms, FoV = 240 × 240 mm, acquisition matrix = 120 × 120, sixty‐one 2‐mm axial slices with no gap, thirty non‐collinear gradient direction with *b* = 1,000 s mm^−2^, one *b* = 0 s mm^−2^, and one repetition. For the structural acquisition, a T1‐weighted magnetization prepared rapid gradient echo sequence was acquired with the following parameters: 176 sagittal slices, TR/TE = 2730/3.48 ms, FA = 7°, slice thickness = 1 mm, slice gap = 0 mm, voxel size = 1 × 1 mm^2^, FoV = 256 mm.

All acquisitions were visually inspected by a certified neuroradiologist, before any pre‐processing step, in order to ensure that none of the individuals had brain lesions and/or critical head motion or artifacts that could affect the quality of the data and reliability of our findings.

A summary of the scanning protocol for the MGH‐HCP data is available at: https://humanconnectome.org/study/hcp-young-adult/document/mgh-adult-diffusion-data-acquisition-details. This protocol included both structural and diffusion scans.

### 2.4 MRI data pre‐processing

#### 2.4.1 Diffusion data

DWI data of the SWITCHBOX dataset was pre‐processed using FMRIB Diffusion Toolbox (FDT) provided with the FMRIB Software Library (FSL v5.0; https://fsl.fmrib.ox.ac.uk/fsl/). Pre‐processing included: correction for motion and eddy current distortions; rotation of gradient vectors accordingly to the affine transformations used to register each volume; extraction and skull stripping of the first b0 volume that created a mask which was then applied to remove non‐brain structures of the remaining volumes; local modeling of diffusion parameters using *bedpostx* algorithm that runs Markov Chain Monte Carlo sampling to build up probability distributions of the diffusion parameters at each voxel and allows modeling of crossing fibers (Behrens, Berg, Jbabdi, Rushworth, & Woolrich, 2007).

Details of the preprocessing procedure applied to the MGH‐HCP dataset are given in (Fan et al., 2014).

#### 2.4.2 Structural data

Structural data of both datasets was processed using the standard semi‐automatic workflow implemented in FreeSurfer toolkit version 6.0 (http://surfer.nmr.mgh.harvard.edu/). In summary, the entire pipeline involves 31 processing steps which include the spatial normalization to Talairach standard space, skull stripping, intensity normalization, tessellation of gray matter (GM)‐white matter (WM) boundary, and cortical, subcortical, and WM segmentation. This pipeline has been validated against manual segmentations (Fischl et al., 2002) and is considered reliable across sessions, scanner platforms, updates, and field strengths (Jovicich et al., 2009). It has suffered several improvements throughout the years and details of the procedures are described in several publications (Desikan et al., 2006; Destrieux, Fischl, Dale, & Halgren, 2010; Fischl et al., 2002). For the present study, the cortical segmentation according to the Desikan–Killiany–Tourville (DKT40) template (A. Klein et al., 2017) and the subcortical segmentation according to the Buckner (Buckner40) template (Fischl et al., 2002) were considered.

### 2.5 Voxel‐wise structural connectivity network construction

Probabilistic tractography was used to estimate connections between brain voxels. The 76 regions of the DKT40 and Buckner40 templates obtained with FreeSurfer were used as seed masks. These masks were first converted to the volumetric space of FSL using in‐house scripts, and then normalized to each subject native diffusion space by applying the affine transformation from diffusion space to structural space. Then, probabilistic tractography was run using *probtrackx2* algorithm from FDT toolbox. 5,000 streamlines were sampled from each voxel in the seed mask. This allowed us to obtain the structural connectivity (SC) profiles of each voxel, by counting the number of streamlines that reached any voxel belonging to any seed mask. Tractography of the SWITCHBOX dataset was performed only for data of the first assessment.

### 2.6 Connectivity‐based parcellation

**FIGURE 1:**
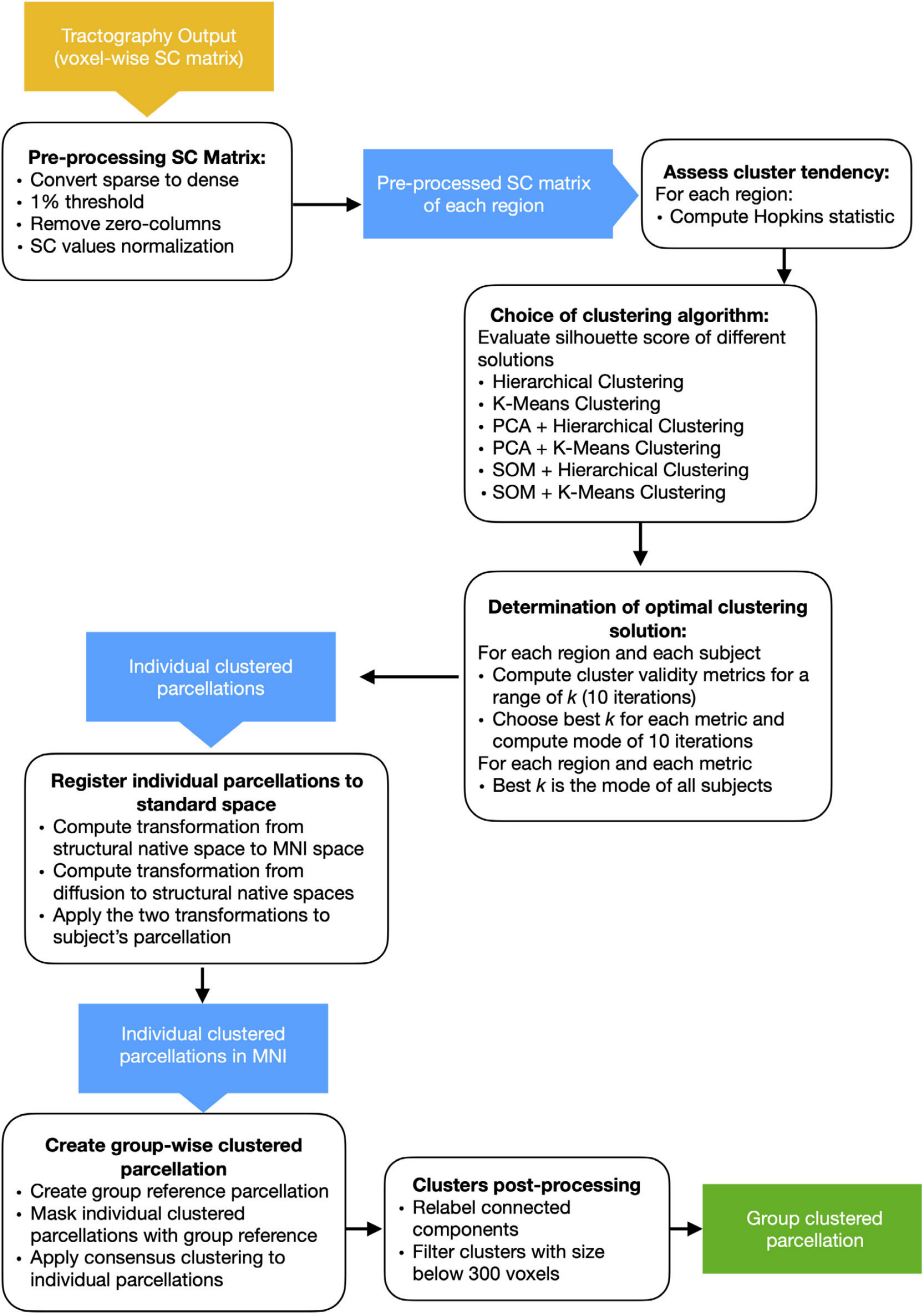
Overview of the workflow employed for the CBP method. Yellow boxes represent the initial input, blue boxes represent intermediate outputs, and green boxes the final output

After running probabilistic tractography, each region was subdivided based on its voxels' connectivity patterns and the results of all subjects were merged in a group‐wise parcellation. The multiple steps performed are described below and an outline of the method can be seen in Figure 1.

#### 2.6.1 Pre‐processing structural connectivity matrix

The output of probabilistic tractography was a sparse voxel‐wise SC matrix for each region, which contains the connectivity values between each voxel of the seed mask and the voxels in all 76 masks (including the mask used as seed). These matrices were pre‐processed before applying the clustering algorithm to group voxels with similar connectivity patterns. First, we performed sparse to dense format conversion, which resulted in a matrix per region with each row representing a voxel of the seed mask and each column the voxels of all 76 masks. Second, a threshold of 1% of the strongest connection was applied to each matrix to remove spurious connections resulting from the probabilistic nature of tractography. After this, the columns of each matrix containing only null elements were removed, given that these represent brain voxels with no connections to seed mask voxels, and thus do not add valuable information for the clustering step. Finally, the connectivity values of each matrix were normalized by applying the Box‐Cox transformation. Different options for normalization of connectivity values were tested, namely log, cubic, and Box–Cox transform, and the latter was selected since it gave the best approximation of a normal distribution, taking into account skewness and kurtosis values, and the histograms with the distribution of values (Figure S1; Table S1).

#### 2.6.2 Assess cluster tendency

Prior to applying clustering algorithms to voxel‐wise connectivity matrices, we assessed the cluster tendency of each region to confirm the existence of clusters. To do this, we calculated the Hopkins statistic for each region. This metric is based on the null hypothesis $H_{0}$ that the data are uniformly distributed and thus has no cluster tendency (Banerjee & Dave, 2004; Hopkins & Skellam, 1954; Pierna & Massart, 2000). In this article, we used a Python implementation of this test (https://pyclustertend.readthedocs.io), where values below 0.5 indicate that there is cluster tendency, while values above 0.5 indicate the presence of uniformly distributed data. This threshold for statistical significance was defined based on the formula to calculate the Hopkins statistic, which is the following:

$$H=\frac{\sum_{i=1}^{n}y_{i}}{\sum_{i=1}^{n}x_{i}+\sum_{i=1}^{n}y_{i}},$$

where $\sum_{i=1}^{n}y_{i}$ represents the distance between each artificial point to the nearest real data point and $\sum_{i=1}^{n}x_{i}$ represents the distance from each real point to each nearest neighbor. So, if the data is uniformly distributed, then $\sum_{i=1}^{n}y_{i}$ and $\sum_{i=1}^{n}x_{i}$ would be close to each other and *H* would be about 0.5. Yet, if clusters are present in the data, the distances for artificial points ($\sum_{i=1}^{n}y_{i}$) would be substantially larger than for the real ones ($\sum_{i=1}^{n}x_{i}$) and so the values of *H* will increase. In this study, we used an implementation of the Hopkins statistic that computed 1‐*H*, thus in our case when clusters are present, the values of *H* will decrease.

We also computed the Hopkins statistic for the voxel‐wise connectivity matrices of the generated parcellation used in the longitudinal analysis to confirm that after clustering the regions do not display cluster tendency.

#### 2.6.3 Choice of clustering algorithm

Several different clustering algorithms can be used to group voxels according to their connectivity profiles. Here, we applied two of the most common used clustering algorithms in CBP: *k*‐means and hierarchical clustering. After clustering was performed, we evaluated the performance of the two algorithms on our dataset to identify the best method for our CBP pipeline. This was done by calculating the silhouette coefficient of the clustering results for each method and selecting the one that scored highest. Silhouette coefficient is an internal cluster validation metric that is used when the ground truth labels are not known, and its value is higher when the clusters are dense and well separated (Rousseeuw, 1987). Furthermore, since the total number of voxels in all brain regions can go up to 200,000 and we are considering connectivity between voxels, our data are high‐dimensional which can undermine the performance of the clustering algorithm. This is known as the curse of dimensionality as termed by Richard Bellman (Bellman, Corporation, & Collection, 1957) and dimensionality reduction techniques can help overcome this issue. As such, we used the silhouette coefficient to evaluate the performance of the two clustering algorithms in conjunction with a dimensionality reduction applied prior to the clustering. Two methods were used, namely principal component analysis (PCA) and self‐organizing maps (SOM). PCA is a linear technique which reduces a large set of variables to a smaller set, known as principal components (PC), while preserving as much of the data's variance as possible (Bishop, 2006). Here, we selected the PCs that preserved 95% of the variance. SOMs are unsupervised learning neural networks that are trained to produce a low‐dimensional representation of the data while preserving the topology of the input space and were inspired by the topographical organization of the sensory cortex of the mammalian brain (Kohonen, 1982, 1990). This method has already been applied to perform CBP of functional data (Mishra, Rogers, Chen, & Gore, 2014).

#### 2.6.4 Determination of optimal clustering solution

After choosing the clustering algorithm, we applied it to each subject and each region for a range of different number of clusters, $k=\left[2:6\right]$. We established the maximum allowed number of clusters to 6, since increasing the number of clusters (e.g., *k =* 10) would lead to very small clusters, with a mean size around 10% of the original region size, which in some cases means the clusters will have sizes of around 4–5 voxels (Figure S2). Regions with a very small number of voxels encompass little information and thus may not bring additional value to the parcellation, in addition to limiting statistical testing. Since the *k =* 6 solution was already linked to a group of fairly small clusters (e.g., left caudate in Figure S2), we decided to set the maximum to 6 clusters. For each k value, we estimated multiple internal cluster validation metrics. Specifically, we computed the Silhouette, Davies–Bouldin, Calinski–Harabasz, and Elbow metrics, which are often used when the ground truth labels are unknown. As the Hopkins statistic, the elbow criterion also allows to test the hypothesis that no clusters are present in the data (i.e., *k* = 1), so we computed this metric for the range $k=\left[1:6\right]$, which allowed us to verify the existence of clusters and prevent to force a cluster structure on the data. The distortion metric was computed for this criterion, which is the average of the squared distances from the cluster centers of the respective clusters. To identify the elbow, we used a python implementation of an algorithm designed to objectively identify the elbow point (https://pypi.org/project/kneed/; Satopaa, Albrecht, Irwin, & Raghavan, 2011).

To circumvent the random initialization associated with clustering algorithms, we run 10 iterations of this procedure for each subject and region. For each run, we selected the best k according to each metric and computed the mode of all these k (i.e., we choose the most frequent k in the 10 iterations run for each metric), which resulted in a k for each subject and region for the four metrics. Following this, we computed the mode of the k per region, over all subjects, to obtain a single k value per region and metric. Finally, we computed the clustering solution for each subject and region according to the selected k of each region and metric. By combining all the clustered regions of a subject, we obtained the individual clustered parcellations. Since we selected different k values based on each of four metrics, this step resulted in four individual parcellation solutions per subject.

#### 2.6.5 Register individual parcellations to standard space

After obtaining the individual clustered parcellations, they were normalized to the Montreal Neurological Institute (MNI) space so that we could combine them to generate the group‐level parcellation. The normalization to the MNI space was performed using Advanced Normalization Tools (ANTs) software package, available at http://stnava.github.io/ANTs/. First, the transformation from native structural space to MNI space was computed, which was composed by an affine and a non‐linear transform. Then, once again an affine and non‐linear transform were concatenated to create the transformation from diffusion to structural native spaces. Finally, the two transformations (diffusion to structural, structural to MNI) were sequentially applied to normalize the subjects' individual clustered parcellations to the MNI space.

#### 2.6.6 Create group‐wise clustered parcellation

Group‐wise parcellation was obtained by combining all individuals' clustered parcellations through a consensus clustering algorithm. This algorithm is used to aggregate multiple partitions of the same dataset (either coming from different clustering algorithms, different runs of the same algorithm, different samples of data) into a single partition (Vega‐Pons & Ruiz‐Shulcloper, 2011). We used the package Cluster Ensembles available at: https://pypi.org/project/Cluster_Ensembles/. This package combines three approximation algorithms to solve the problem of maximizing the average similarity between partitions and the one with the best performance is selected (Giecold, Marco, Garcia, Trippa, & Yuan, 2016; Strehl & Ghosh, 2002).

First, we created a group reference parcellation, since our initial parcellation (DKT40 and Buckner40) was obtained for each individual as a result of FreeSurfer's segmentation. For this, we transformed each individual parcellation from structural native space to the MNI space using ANTs, as described before. Then, for each voxel, we attributed a label that was the mode of all subjects. Finally, we applied a threshold of 20% of the total number of subjects in order to remove voxels that were only present in few subjects. We chose this threshold because it had a good coverage of GM and did not include too much WM (Figure S3). After obtaining the group reference parcellation, we masked each individual clustered parcellation using this reference and then applied consensus clustering to each region. In the end, we combined all regions to create a group clustered parcellation of the whole brain.

#### 2.6.7 Clusters postprocessing

Since the clustering algorithm can generate spatially disjoint clusters, we forced these to be spatially contiguous by relabeling connected components. Thus, each connected component in a region was assigned to a different cluster. This means that for some regions the final number of clusters was higher than the chosen k.

Finally, clusters with a size under 300 voxels (SWITCHBOX) and 200 voxels (MGH‐HCP) were removed by merging them with its neighborhood. These sizes were chosen after evaluating the clusters' sizes of the group parcellation registered to each individual's native diffusion space. Since subjects' diffusion space had lower resolution (2 × 2 × 2 mm) when compared to the resolution of the group parcellation (1 × 1 × 1 mm), the clusters' sizes registered in the native diffusion space had less voxels. We opted for selecting the threshold level leading to a minimum cluster size higher than 5 voxels in the subjects' diffusion space (Figure S4).

### 2.7 Connectivity homogeneity fingerprint

We developed a metric to evaluate parcellation homogeneity and thus compare the accuracy of different parcellations in terms of regional connectivity fingerprint homogeneity—connectivity homogeneity fingerprint (CHF). This metric reflects homogeneity level of the structural connectivity fingerprint of all voxels in a region (i.e., the magnitude of overlap between the structural connectivity fingerprint of all voxels contained a region). Higher values mean that a region contains a larger pool of voxels with homogeneous connectivity profiles to the rest of the brain. It is calculated using the voxel‐wise SC matrix of each region with the following steps: (a) the matrix is binarized; (b) the mean of each column is computed, which results in a vector with the average of seed voxels that connects to each target voxel, thus we remove the effect of seed size in the CHF metric; (c) a 1% threshold is applied to remove spurious connections; (d) the CHF is equal to the average of the vector and so the target's size has no effect on the metric.

#### 2.7.1 Comparison to null model

Given that metrics of homogeneity are likely to depend on cluster size and the total number of clusters delineated, we compared the CHF of the final parcellation with a null model. With this comparison, we are able to verify if the generated clusters are more homogeneous than would be expected from randomly placed clusters of the same shape and size. We used a similar approach to the one proposed by Gordon and colleagues (Gordon et al., 2016). In summary, we registered the final parcellation to the surface space and then rotated each hemisphere a random amount around each axis (*x*, *y*, and *z*). This ensures that the relocated clusters maintain the relative positions to each other and their original number of vertices. Clusters that were rotated into the medial wall were discarded from the analysis. Then, we registered the rotated versions of the parcellation back to the volume space and calculated the CHF. We repeated this procedure 100 times for the SWITCHBOX dataset. To avoid potential limitations associated to the conversion to the surface space, the CHF calculation was limited to voxels comprised in the cortical mesh of the final parcellation, which eliminated voxels of subcortical areas. Moreover, although the rotated versions of the parcellation in surface space had equal sizes for the same region, this was not observed in the volume space, due to the conversions between these two spaces. Thus, there is a slightly variation in regional size between each version of the rotated parcellation. Statistical comparison between the final parcellation and the rotated parcellations was performed by computing a *Z*‐score, as described in (Gordon et al., 2016):

$$z=\frac{\mathrm{CH}F_{\text{original}}-\frac{1}{n}\sum_{i=1}^{n}\mathrm{CH}F_{\text{rotate}d_{i}}}{\sigma_{\mathrm{CH}F_{\text{rotated}}}}.$$

#### 2.7.2 Evaluation of parcellations

Since the main goal of CBP is to group voxels with similar connectivity patterns, we evaluated the CHF of the original and the final parcellation to verify if our CBP method resulted in a parcellation with higher CHF. To do this, we performed probabilistic tractography with the obtained group parcellation. First, the group parcellation was registered to each individual's diffusion space with ANTs, by applying sequentially the transformation from MNI space to native structural space (composed by an affine and a non‐linear transform) and the transformation from structural to diffusion native spaces (composed by an affine and a non‐linear transform). Gray matter and white matter masks, estimated with FreeSurfer for each individual, were applied to the group parcellation to exclude voxels outside these regions (e.g., voxels belonging to cerebrospinal fluid [CSF]). Then, probabilistic tractography was run using the group parcellation clusters as seed masks (5,000 streamlines were sampled from each voxel in the seed mask). This allowed to obtain the voxel‐wise SC matrix for each cluster (i.e., SC between each voxel of the cluster and the voxels of all clusters in the parcellation) and then we calculated the CHF of each cluster for all subjects. The CHF of the original parcellation was computed with the voxel‐wise SC matrices that were used as the input of CBP. Statistical comparison of the CHF between each developed parcellation and the original parcellation was performed using independent samples *t* tests and *p* values were corrected for multiple comparisons, using the false discovery rate method.

### 2.8 Selection of group parcellation

After evaluating the CHF of the group parcellations estimated with each cluster validity metric (i.e., Silhouette, Davies–Bouldin, Calinski–Harabasz, Elbow), we selected one group parcellation to assess longitudinal changes in the topological properties of white matter structural connectivity networks. To do this, we repeated some of the steps described in the previous section for the second timepoint dataset of the SWITCHBOX cohort, with the selected partition (registration of group parcellation to individual's diffusion space, probabilistic tractography, and calculation of CHF).

### 2.9 Network construction

We created the SC network matrices, for each subject and timepoint, by performing probabilistic tractography using the clusters of the selected group parcellation as seed masks. This resulted in an SC matrix, for each subject, representing the number of streamlines leaving each seed mask and reaching any of the other regions. This matrix was normalized by dividing each line by the waytotal value (i.e., the total number of generated tracts not rejected by inclusion/exclusion mask criteria). We further divided the matrix by its maximum value, in order to have connectivity values between [0, 1]. Since tractography is dependent on seeding location, the connectivity probability from *i* to *j* is not necessarily equal to that from *j* to *i*. Still, these two probabilities are highly correlated across the brain for all participants. Thus, we defined the undirected connectivity probability as the average of these two probabilities, $P_{\mathrm{ij}}$ and $P_{\mathrm{ji}}$, which originated an undirected connectivity matrix. Next, a consistency‐based threshold was applied, which retains the most consistent connections across subjects with the aim of reducing the false‐positive in group‐average connectivity matrices (Roberts, Perry, Roberts, Mitchell, & Breakspear, 2017). We applied this threshold at a 30% density, the same value used in the original work describing the technique (Roberts et al., 2017). Subsequently, we tested different strategies to account for connectivity between clusters of the same region: (a) set intra‐cluster connectivity to 0 and normalize regional connectivity values by the maximum; (b) set intra‐cluster connectivity to 1 and normalize regional connectivity values by the maximum (excluding the intra‐cluster connectivity); and (c) use the original intra‐cluster connectivity values. Figure S5 shows the example for one subject of the connections surviving each of these strategies. We opted to use the original intra‐cluster connectivity values as the other two strategies seem to include more connections which could lead to the inclusion of false‐positive connections. Finally, a threshold set to 1% of the strongest connection was applied to each SC matrix, in order to remove spurious connections that were consistent across participants and thus were not removed with the consistency threshold. This 1% threshold has been proven to remove edges with poor to moderate reproducibility, while maintaining edges with moderate to good reproducibility (Tsai, 2018). Thus, it is suitable to threshold structural connectivity matrices for network analysis.

### 2.10 Graph theoretical analysis

Brain networks can be described in terms of its topological organization, using graph theory measures. Brain Connectivity Toolbox (https://sites.google.com/site/bctnet/) was used to extract these metrics. The structural connectivity networks built with the selected group parcellation were used. The following topological features were evaluated for both timepoints: modularity and hubs (global, provincial, and connector). The description of these metrics is detailed in Appendix S1.

### 2.11 Fingerprints of modular connectivity

We also analyzed, for each timepoint, the network fingerprints of inter‐modular (global and connector‐hub‐driven) and intra‐modular connectivity. The same method of analysis as described in Fernandes et al. (2019)), was applied in this study. In summary, modular connectivity strength was defined as the degree (total number of connections) of all nodes constituting a module. To quantify this connectivity at both timepoints, a reference scheme of community structure was chosen based on the mean score of community‐structure goodness‐of‐fit. Subsequently, matrices of inter‐modular and intra‐modular connectivity were created for both timepoints.

### 2.12 Statistical analysis

Statistical comparison of the SC matrices between first and last assessments, at the edge level, was performed by applying a paired sample *t* test with SC as the dependent variable and time of evaluation as independent variable. The obtained SC networks are comprised of 170 nodes, yielding a total number of possible edges of 14,365 (170×169/2). Testing the hypothesis of interest at the edge level, therefore poses a multiple comparisons problem. In order to increase the statistical power of the analysis, we used the network‐based statistics (NBS) procedure implemented in the NBS toolbox (https://sites.google.com/site/bctnet/comparison/nbs). This is a non‐parametric statistical method that allows the identification of significantly different sub‐networks, while controlling for the family‐wise error rate (FWER; Zalesky, Fornito, & Bullmore, 2010). First, it independently tests the hypotheses at every connection in the network and threshold the ones exceeding a user defined primary threshold, then it identifies sub‐networks constituted by interconnected edges that survived the primary threshold. The significance of these sub‐networks is then calculated by comparing their sizes to the distribution of the size of sub‐networks obtained through random permutations of the original hypothesis. It is important to note that the primary threshold only affects the sensitivity of the method and thus, FWER is assured independently of this threshold. In this study, the primary threshold was set to *F* = 7.0, which was the maximum threshold that detected a unique significant connected component having more than two connections (Figure S6). Longitudinal changes in structural connectivity detected with NBS are represented by significantly connected components at a corrected level of *p* < .05 FWE corrected.

## 3 RESULTS

### 3.1 Sample characterization

Table 1 shows the demographic characterization of the participants from the SWITCHBOX dataset included in this study. In summary, mean age at baseline was 63.5 years (range, 51–82 years) and mean interval between evaluations was 52.8 months (range, 45–73 months). Interval time was not strongly associated with age at baseline (*r* = −.12, *p* = .41). The sample was balanced for sex (51% females, 49% males) and they did not differ with respect to interval time ($t\left(30\right)=0.14,p=.89$). Mean education level was 5.98 years (range, 0–17 years). The MGH‐HCP dataset used in this study included 32 healthy adults (age range 20–59 years, 56% males).

**TABLE 1:** Basic demographic characterization of the study's cohort

	Mean ± *SD* (range)
*N* (females/males)	51 (26/25)
Age at baseline (years)	63.5 ± 7.41 (51–82)
Age at follow‐up (years)	68.0 ± 7.25 (55–86)
Interval (months)	52.8 ± 7.24 (45–73)
Education (years)	5.98 ± 3.97 (0–17)

### 3.2 Cluster tendency

For both parcellations (original—DKT40; generated—Silhouette), all regions exhibited cluster tendency (Figures S7 and S8). Although we observed an increase in the Hopkins statistic, the final clusters, independently of regional size, still display cluster tendency according to this statistic. This might happen because the Hopkins statistic is a method to determine the type of distribution of the data (random, aggregate, and regular), while to assess cluster tendency the goal is to verify if our data is generated from a multimodal distribution (i.e., if our data contain multiple clusters, the pairwise distances distribution will have a group of small distances corresponding to within‐cluster distances, and a group of large distances corresponding to between‐cluster distances; Adolfsson, Ackerman, & Brownstein, 2019). For this reason, the Hopkins statistic may fail to assess cluster tendency. Thus, we decided not to consider this metric to assess cluster tendency and only used the elbow criterion.

### 3.3 Clustering algorithm

**FIGURE 2:**
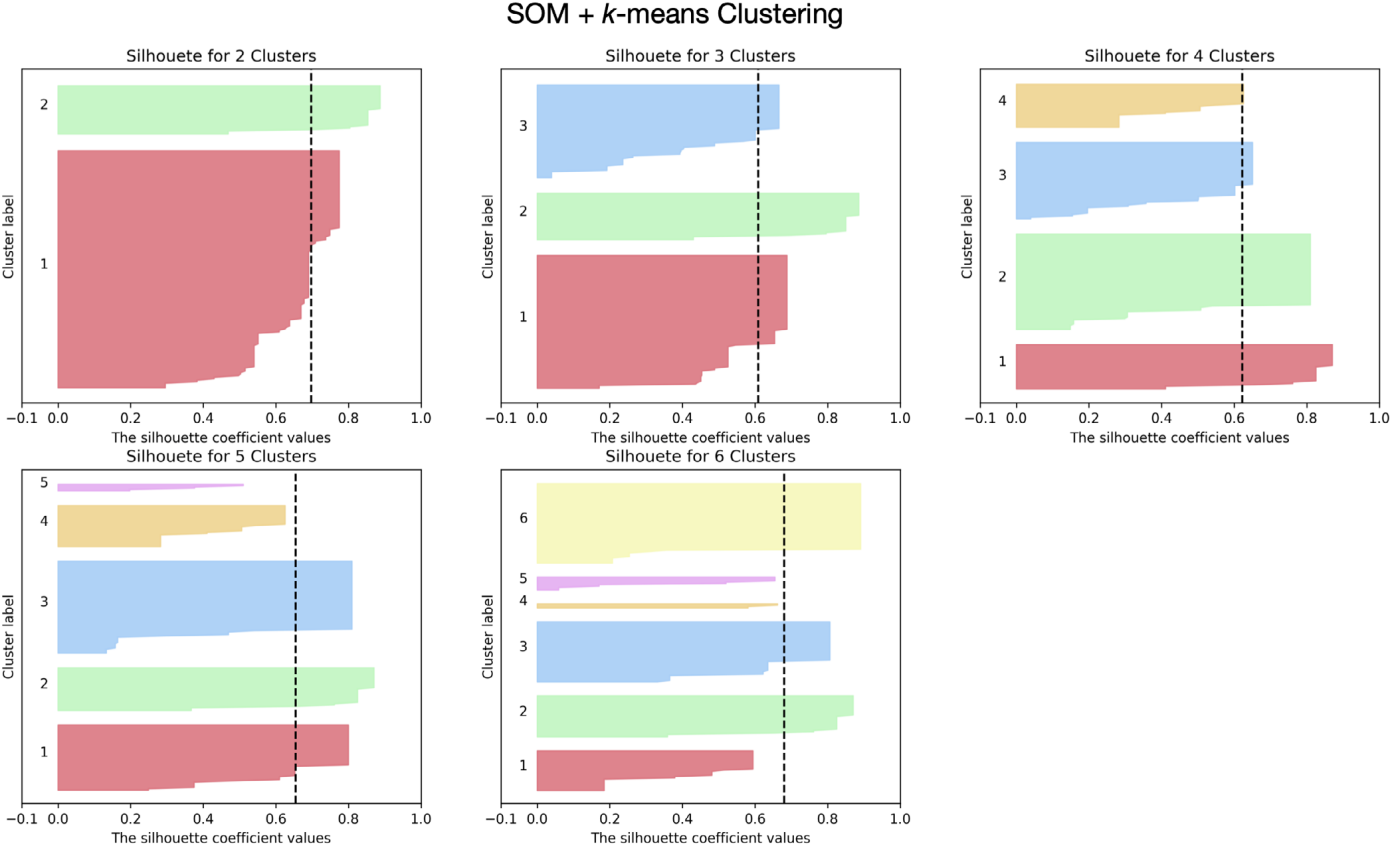
Example of silhouette scores of one brain region for *k*‐means clustering in conjunction with SOM data dimensionality reduction technique. Different clustering solutions (2–6 clusters) were tested. The black dashed line represents the mean silhouette score across all data samples. This approach (SOM + *k*‐means clustering) presents the highest values of silhouette coefficient and with more balanced cluster sizes

The analysis of the silhouette scores of different clustering algorithms revealed that the best solution was the *k*‐means algorithm in conjunction with SOM for dimensionality reduction. Figure 2 represents the silhouette coefficient scores for different number of clusters for one brain region using this solution (for the other solutions, see Figures S9–S13). The *k*‐means + SOM solution in addition to result in higher silhouette scores also originated clusters more balanced in terms of size.

### 3.4 Optimal clustering solution

Table 2 presents the optimal number of clusters for each brain region according to each cluster validity metric (Silhouette, Davies–Bouldin, Calinski–Harabasz, and Elbow). In the SWITCHBOX cohort, for the Silhouette coefficient, most of regions were partitioned in two clusters, with the exception of right entorhinal cortex, left and right caudate, right amygdala, and left and right accumbens area which were subdivided in six clusters. Davies–Bouldin score also subdivided almost all brain regions in two clusters, with the exception of right transverse temporal, left and right accumbens area that were subdivided in six clusters, and right amygdala which was partitioned in three clusters. According to the Calinski–Harabasz coefficient, all brain regions were split in six clusters. Finally, the Elbow score subdivided most of the regions in two clusters, with the exception of left entorhinal, left isthmus cingulate, right transverse temporal, bilateral amygdala, and bilateral accumbens area which were partitioned in three clusters. In the MGH‐HCP cohort, Silhouette, Davies–Bouldin, and Elbow coefficients subdivided the majority of regions in two clusters, while Calinski–Harabasz score subdivided all regions in six clusters.

**TABLE 2:** Optimal number of clusters for each brain region according to each clustering validity metric

	Silhouette				Davies–Bouldin				Calinski–Harabasz				Elbow			
ROI name	$n_{\text{left}}$		$n_{\text{right}}$		$n_{\text{left}}$		$n_{\text{right}}$		$n_{\text{left}}$		$n_{\text{right}}$		$n_{\text{left}}$		$n_{\text{right}}$	
	SW	HCP	SW	HCP	SW	HCP	SW	HCP	SW	HCP	SW	HCP	SW	HCP	SW	HCP
Caudal anterior cingulate	2	2	2	2	2	2	2	2	6	6	6	6	2	2	2	2
Caudal middle frontal	2	6	2	6	2	2	2	4	6	6	6	6	2	2	2	2
Cuneus	2	2	2	2	2	2	2	2	6	6	6	6	2	2	2	2
Entorhinal	2	2	6	2	2	2	2	2	6	6	6	6	3	3	2	2
Fusiform	2	6	2	6	2	2	2	2	6	6	6	6	2	2	2	2
Inferior parietal	2	6	2	2	2	2	2	2	6	6	6	6	2	2	2	2
Inferior temporal	2	6	2	6	2	2	2	2	6	6	6	6	2	2	2	2
Isthmus cingulate	2	2	2	2	2	2	2	2	6	6	6	6	3	2	2	2
Lateral occipital	2	2	2	2	2	2	2	2	6	6	6	6	2	2	2	2
Lateral orbitofrontal	2	2	2	2	2	2	2	2	6	6	6	6	2	2	2	2
Lingual	2	2	2	2	2	2	2	2	6	6	6	6	2	2	2	2
Medial orbitofrontal	2	2	2	2	2	2	2	2	6	6	6	6	2	2	2	2
Middle temporal	2	2	2	2	2	2	2	2	6	6	6	6	2	2	2	2
Parahippocampal	2	2	2	2	2	2	2	2	6	6	6	6	2	3	2	2
Paracentral	2	2	2	2	2	2	2	2	6	6	6	6	2	2	2	2
Pars opercularis	2	6	2	6	2	2	2	2	6	6	6	6	2	2	2	2
Pars orbitalis	2	6	2	6	2	3	2	2	6	6	6	6	2	3	2	2
Pars triangularis	2	6	2	6	2	2	2	3	6	6	6	6	2	2	2	2
Pericalcarine	2	2	2	2	2	2	2	2	6	6	6	6	2	3	2	2
Postcentral	2	2	2	2	2	2	2	2	6	6	6	6	2	2	2	2
Posterior cingulate	2	2	2	2	2	2	2	2	6	6	6	6	2	2	2	2
Precentral	2	2	2	2	2	2	2	2	6	6	6	6	2	2	2	2
Precuneus	2	2	2	2	2	2	2	2	6	6	6	6	2	2	2	2
Rostral anterior cingulate	2	6	2	6	2	2	2	4	6	6	6	6	2	2	2	3
Rostral middle frontal	2	2	2	2	2	2	2	2	6	6	6	6	2	2	2	2
Superior frontal	2	2	2	2	2	2	2	2	6	6	6	6	2	2	2	2
Superior parietal	2	6	2	6	2	2	2	2	6	6	6	6	2	2	2	2
Superior temporal	2	2	2	2	2	2	2	2	6	6	6	6	2	2	2	2
Supramarginal	2	2	2	6	2	2	2	2	6	6	6	6	2	2	2	2
Transverse temporal	2	2	2	6	2	4	6	4	6	6	6	6	2	3	3	3
Insula	2	2	2	6	2	2	2	2	6	6	6	6	2	2	2	2
Thalamus proper	2	2	2	2	2	2	2	2	6	6	6	6	2	2	2	2
Caudate	6	2	6	2	2	2	2	2	6	6	6	6	2	2	2	2
Putamen	2	2	2	2	2	2	2	2	6	6	6	6	2	2	2	2
Pallidum	2	2	2	2	2	2	2	2	6	6	6	6	2	2	2	2
Hippocampus	2	2	2	2	2	2	2	2	6	6	6	6	2	2	2	2
Amygdala	2	3	6	3	2	3	3	3	6	6	6	6	3	2	3	2
Accumbens area	6	3	6	2	6	3	6	2	6	6	6	6	3	2	3	2

Abbreviations: HCP, MGH‐HCP dataset; $n_{\text{left}}$, number of clusters for the left region; $n_{\text{right}}$, number of clusters for the right region; SW, SWITCHBOX dataset.

The final group clustered parcellations according to each metric are represented in Figure S14. Silhouette coefficient generated a group partition with 170 clusters (SWITCHBOX) and 241 clusters (MGH‐HCP), Davies–Bouldin, a group parcellation with 163 clusters (SWITCHBOX) and 176 clusters (MGH‐HCP), Calinski–Harabasz produced a group partition with 472 clusters (SWITCHBOX) and 444 clusters (MGH‐HCP), and Elbow metric generated a group parcellation with 165 clusters (SWITCHBOX) and 167 clusters (MGH‐HCP).

### 3.5 Connectivity homogeneity fingerprint

**FIGURE 3:**
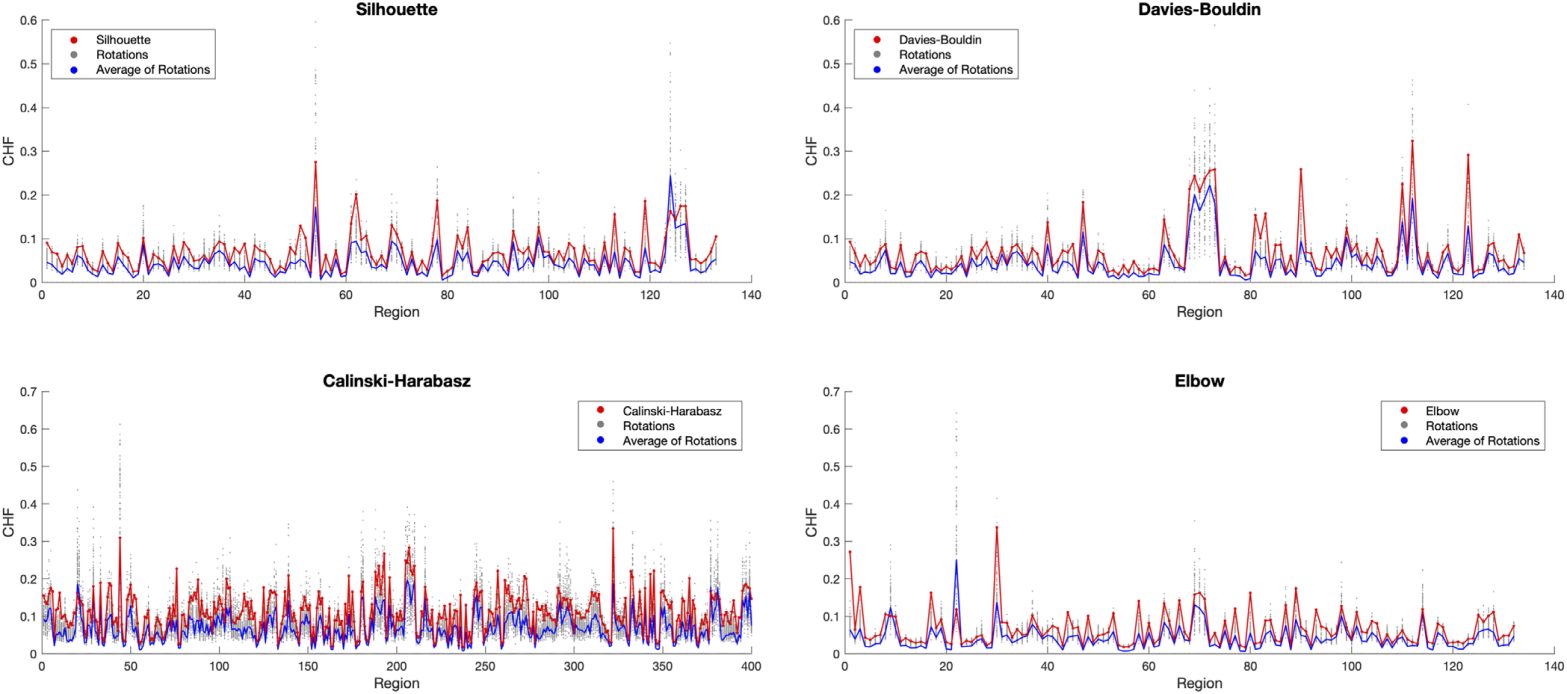
Mean connectivity homogeneity fingerprint (CHF) scores for each group parcellation and for the 100 rotated parcellations, in the SWITCHBOX dataset. Red line represents CHF of each group parcellation (Silhouette, Davies–Bouldin, Calinski–Harabasz, and Elbow), blue line represents the average CHF of the 100 rotated parcellations, and gray dots represent CHF of each rotated parcellation. For all the four parcellations, the CHF is higher than for the mean of the rotated null parcellations

The CHF of each parcellation was higher than the mean of 100 rotated parcellations for the majority of regions (Figure 3). Only a few regions in the Silhouette, Calinski–Harabasz, and Elbow presented higher CHF for the null model parcellations, and these correspond to very small regions, which increases the probability, when we rotate the parcellation, to that cluster be rotated to a region with high homogeneity. Regarding *Z*‐scores, the Silhouette parcellation had 5.98 (i.e., was 5.98 *SD* away from the mean CHF of the null model parcellations), the Davies–Bouldin had 6.04, the Calinski–Harabasz had 9.96, and the Elbow had 6.24.

**FIGURE 4:**
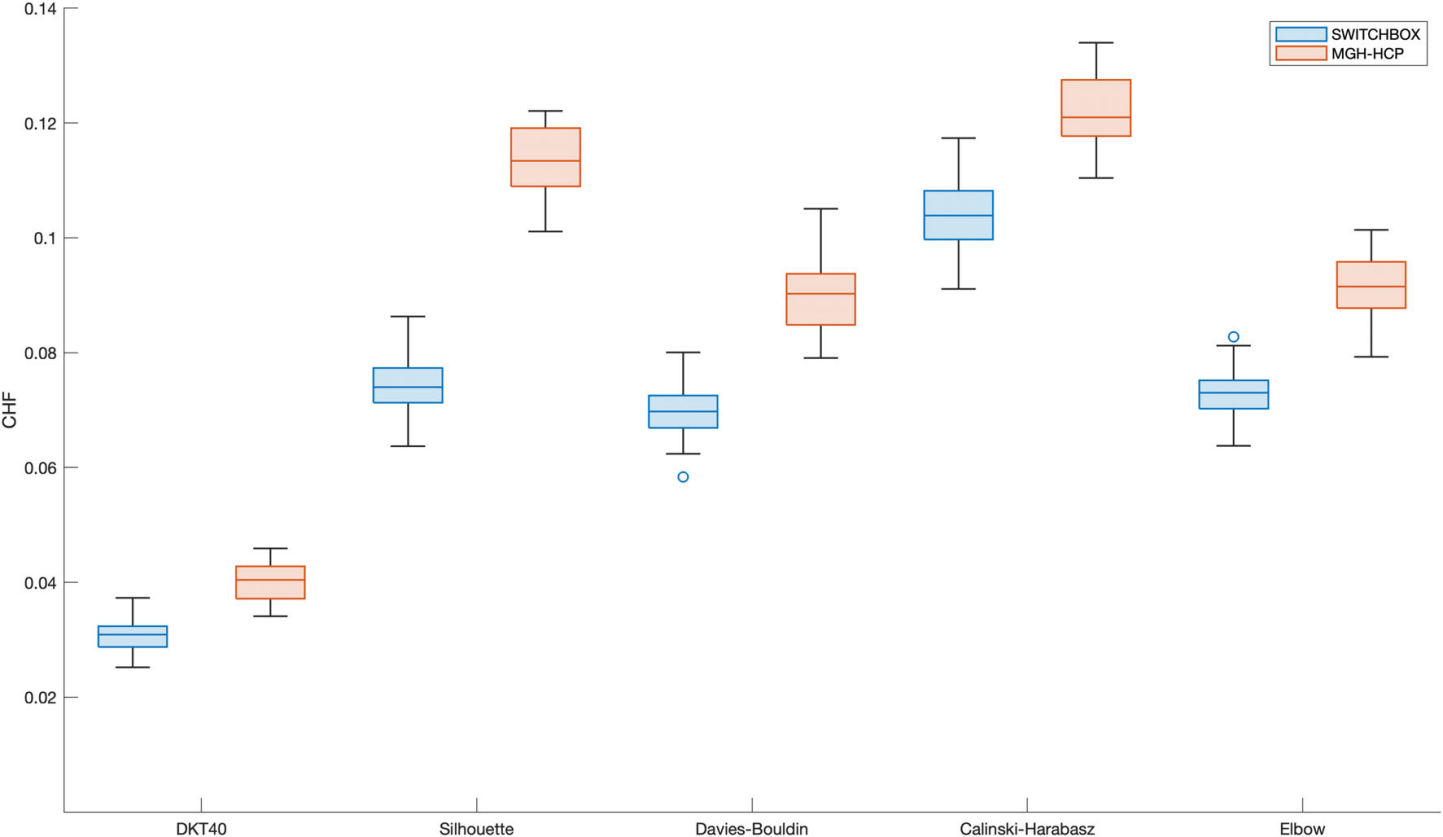
Mean connectivity homogeneity fingerprint (CHF) scores of the different group parcellations for all subjects. The four solutions resulted in parcellations with higher CHF in comparison to the original partition. Calinski–Harabasz parcellation had the highest homogeneity values but also the highest number of clusters

**FIGURE 5:**
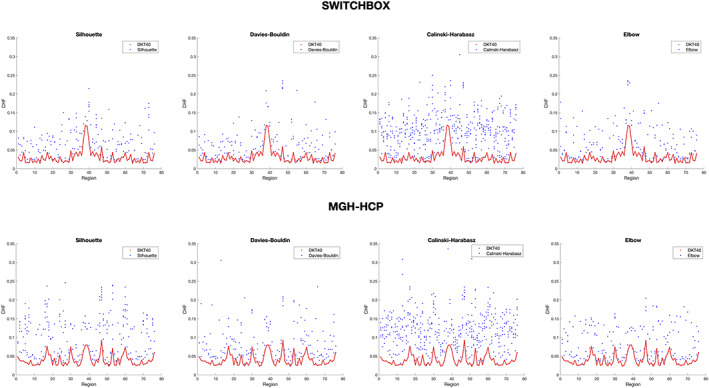
Mean connectivity homogeneity fingerprint (CHF) scores of the regions of the original partition and the individual clusters of the four group parcellations. Red line represents CHF of the original parcellation (DKT40) and blue dots represent CHF of each individual cluster of the group parcellations (Silhouette, Davies–Bouldin, Calinski–Harabasz, and Elbow). Individual clusters belonging to the same region of the original partition present the same value in the *x*‐axis. For all the group parcellations, the CHF of the individual clusters is higher than the CHF of the original region

The four group parcellations present higher values of CHF in comparison to the initial parcellation (DKT40 + Buckner40; Figure 4). For the SWITCHBOX cohort, the parcellations produced from Silhouette, Davies–Bouldin, and Elbow coefficients exhibit similar values of CHF (Silhouette—*M* = 0.074, *SD* = 0.005; Davies–Bouldin—M=0.070,SD=0.004; Elbow—*M* = 0.073, *SD* = 0.004); the one originating from Calinski–Harabasz has the highest value (*M* = 0.10, *SD* = 0.006). For the MGH‐HCP dataset, Silhouette and Calinski–Harabasz had similar CHF values (Silhouette—M=0.11,SD=0.006; Calinski–Harabasz—*M* = 0.12, *SD* = 0.006), while Davies–Bouldin and Elbow partitions had lower and equal values (Davies–Bouldin—*M* = 0.09, *SD* = 0.006; Elbow—*M* = 0.09, *SD* = 0.006). The initial partition displays the lowest value for both datasets (SWITCHBOX: M=0.031,SD=0.003; MGH‐HCP: M=0.040,SD=0.003). Statistical comparison of CHF values revealed that the four parcellations had statistically significant higher CHF values in comparison to the original parcellation (SWITCHBOX: Silhouette – $t_{\left(76.3\right)}=57.6,p<.001,d=11.4$; Davies–Bouldin – $t_{\left(79.4\right)}=54.0,p<.001,d=10.7$; Calinski–Harabasz – $t_{\left(66.7\right)}=78.7,p<.001,d=15.6$; Elbow – $t_{\left(80\right)}=58.8,p<.001,d=11.6$; MGH‐HCP: Silhouette – $t_{\left(49.2\right)}=63.7,p<.001,d=15.9$; Davies–Bouldin – $t_{\left(47.9\right)}=41.4,p<.001,d=10.3$; Calinski–Harabasz – $t_{\left(47.8\right)}=68.8,p<0.001,d=17.2$; Elbow – $t_{\left(49.7\right)}=45.2,p<.001,d=11.3$). Figure 5 shows the CHF values, for each cluster of the group parcellations and the original region of the initial parcellation. We observe that the CHF of the individual clusters is higher than the original region for all parcellations and for all regions.

We selected the estimated group parcellation based on the Silhouette score to assess longitudinal changes in white matter structural connectivity of the SWITCHBOX cohort. The Calinski–Harabasz parcellation has a very high number of clusters, which may in part explain its high value of CHF, but this increased granularity may not be beneficial and can make subsequent analyses computationally expensive. Furthermore, the Davies–Bouldin partition had some regions that were clustered in a pattern that may not be biologically plausible (checkerboard pattern, Figure S15) and Silhouette parcellation had slightly higher mean CHF values in comparison the Elbow partition. Thus, we selected the Silhouette parcellation which does not present these limitations and still has higher CHF in comparison to the initial parcellation. Details of the label and coordinates of the regions belonging to this parcellation are given in Table S2.

**FIGURE 6:**
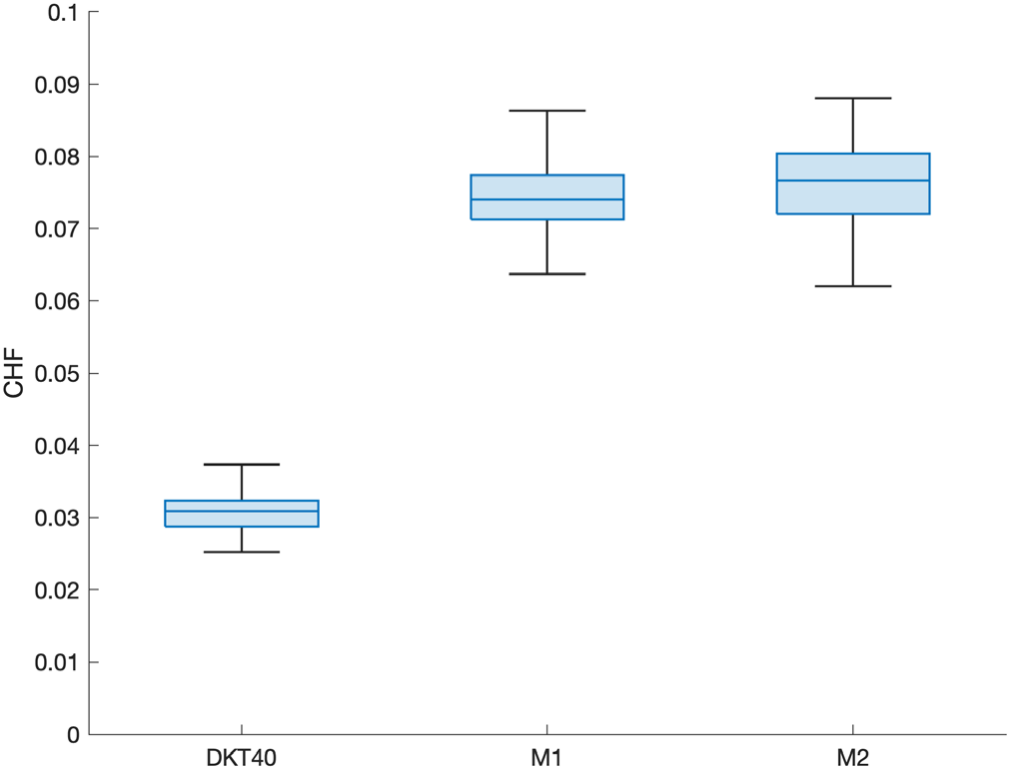
Mean CHF values for the two timepoints and the initial parcellation. At both timepoints, the homogeneity is higher in comparison to the original partition

In addition, in the second timepoint, the values of CHF for the Silhouette parcellation are very similar when compared to the first timepoint (M=0.077,SD=0.006, Figure 6).

### 3.6 Longitudinal changes in brain structural connectivity

**FIGURE 7:**
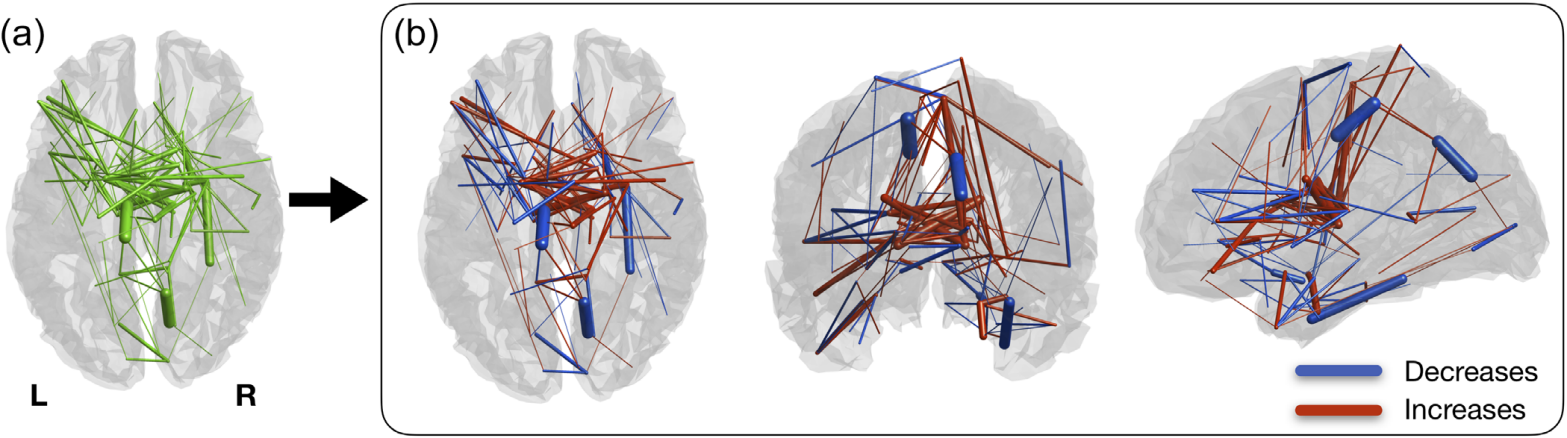
Significant changes in structural connectivity between timepoints. (a) Binarized version of the connected component of significantly altered structural connectivity. (b) Weighted version of (a), with edge thickness representing the amplitude of differences. Blue represents decreases in connectivity strength between timepoints and red represents increases. Connections with decreases are mostly intra‐hemispheric, while most of the increases are composed of intra‐hemispheric connections

Using the Silhouette parcellation, we found significant changes in structural connectivity between timepoints in a brain sub‐network (*p* < .001), comprising 122 connections, from which 52 correspond to decreases and 70 to increases in structural connectivity (Figure 7). When analyzing the individual connections of this sub‐network, we concluded that the connections with longitudinal decreases in connectivity are represented by 19 intra‐left, 24 intra‐right, and 9 inter‐hemispheric connections. The connections with longitudinal increases in connectivity are composed by 16 intra‐left, 22 intra‐right, and 32 inter‐hemispheric connections. The summary of the connections is present in Table 3.

**TABLE 3:** Description of the connections comprising the connected component of significant structural connectivity differences between timepoints (*p* < .001)

Area 1		Area 2		Difference	Intra‐left	Intra‐right	Inter‐hemispheric
*N*	Name	*N*	Name				
Increases							
68	Left thalamus proper 2	99	Right caudate 4	0.035	0	0	1
71	Left caudate 3	102	Right thalamus proper 1	0.033	0	0	1
85	Right amygdala 2	160	Right entorhinal 1	0.029	0	1	0
102	Right thalamus proper 1	140	Right paracentral 2	0.027	0	1	0
70	Left caudate 2	101	Right caudate 6	0.026	0	0	1
85	Right amygdala 2	89	Right amygdala 6	0.024	0	1	0
70	Left caudate 2	99	Right caudate 4	0.023	0	0	1
34	Left pars orbitalis 1	73	Left caudate 5	0.022	1	0	0
70	Left caudate 2	96	Right caudate 1	0.022	0	0	1
71	Left caudate 3	103	Right thalamus proper 2	0.018	0	0	1
37	Left pars triangularis 2	73	Left caudate 5	0.018	1	0	0
115	Right superior frontal 2	123	Right precentral 1	0.017	0	1	0
20	Left lateral orbitofrontal 2	26	Left middle temporal 2	0.016	1	0	0
73	Left caudate 5	96	Right caudate 1	0.016	0	0	1
67	Left thalamus proper 1	99	Right caudate 4	0.015	0	0	1
15	Left isthmus cingulate 1	153	Right isthmus cingulate 2	0.015	0	0	1
49	Left rostral anterior cingulate 1	72	Left caudate 4	0.015	1	0	0
92	Right pallidum 1	140	Right paracentral 2	0.013	0	1	0
68	Left thalamus proper 2	96	Right caudate 1	0.013	0	0	1
69	Left caudate 1	102	Right thalamus proper 1	0.013	0	0	1
95	Right putamen 2	130	Right postcentral 3	0.013	0	1	0
73	Left caudate 5	102	Right thalamus proper 1	0.012	0	0	1
142	Right parahippocampal 2	154	Right inferior temporal 1	0.011	0	1	0
102	Right thalamus proper 1	139	Right paracentral 1	0.011	0	1	0
67	Left thalamus proper 1	101	Right caudate 6	0.011	0	0	1
94	Right putamen 1	120	Right rostral anterior cingulate 2	0.011	0	1	0
94	Right putamen 1	111	Right superior temporal 2	0.011	0	1	0
90	Right hippocampus 1	96	Right caudate 1	0.010	0	1	0
44	Left posterior cingulate 2	140	Right paracentral 2	0.010	0	0	1
115	Right superior frontal 2	124	Right precentral 2	0.009	0	1	0
67	Left thalamus proper 1	96	Right caudate 1	0.009	0	0	1
92	Right pallidum 1	121	Right precuneus 1	0.009	0	1	0
83	Right accumbens area 1	105	Right insula 2	0.009	0	1	0
97	Right caudate 2	99	Right caudate 4	0.009	0	1	0
15	Left isthmus cingulate 1	122	Right precuneus 2	0.008	0	0	1
30	Left paracentral 1	122	Right precuneus 2	0.008	0	0	1
70	Left caudate 2	97	Right caudate 2	0.007	0	0	1
71	Left caudate 3	115	Right superior frontal 2	0.007	0	0	1
34	Left pars orbitalis 1	68	Left thalamus proper 2	0.007	1	0	0
54	Left superior frontal 1	71	Left caudate 3	0.007	1	0	0
31	Left paracentral 2	121	Right precuneus 1	0.007	0	0	1
70	Left caudate 2	140	Right paracentral 2	0.006	0	0	1
161	Right entorhinal 2	164	Right entorhinal 5	0.006	0	1	0
3	Left caudal middle frontal 1	32	Left pars opercularis 1	0.006	1	0	0
15	Left isthmus cingulate 1	165	Right cuneus 1	0.006	0	0	1
7	Left entorhinal 1	78	Left hippocampus 1	0.006	1	0	0
31	Left paracentral 2	122	Right precuneus 2	0.006	0	0	1
69	Left caudate 1	140	Right paracentral 2	0.006	0	0	1
74	Left putamen 1	101	Right caudate 6	0.005	0	0	1
35	Left pars orbitalis 2	73	Left caudate 5	0.005	1	0	0
73	Left caudate 5	103	Right thalamus proper 2	0.005	0	0	1
29	Left parahippocampal 2	38	Left pericalcarine 1	0.005	1	0	0
74	Left putamen 1	115	Right superior frontal 2	0.005	0	0	1
4	Left caudal middle frontal 2	37	Left pars triangularis 2	0.005	1	0	0
40	Left postcentral 1	140	Right paracentral 2	0.005	0	0	1
101	Right caudate 6	149	Right lateral orbitofrontal 2	0.005	0	1	0
67	Left thalamus proper 1	97	Right caudate 2	0.004	0	0	1
76	Left pallidum 1	116	Right superior frontal 3	0.004	0	0	1
26	Left middle temporal 2	39	Left pericalcarine 2	0.004	1	0	0
102	Right thalamus proper 1	136	Right pars orbitalis 2	0.004	0	1	0
151	Right lateral occipital 2	166	Right cuneus 2	0.004	0	1	0
101	Right caudate 6	134	Right pars triangularis 2	0.004	0	1	0
31	Left paracentral 2	67	Left thalamus proper 1	0.004	1	0	0
19	Left lateral orbitofrontal 1	26	Left middle temporal 2	0.003	1	0	0
131	Right pericalcarine 1	158	Right fusiform 1	0.003	0	1	0
95	Right putamen 2	115	Right superior frontal 2	0.003	0	1	0
21	Left lingual 1	28	Left Parahippocampal 1	0.003	1	0	0
77	Left pallidum 2	98	Right caudate 3	0.002	0	0	1
73	Left caudate 5	78	Left hippocampus 1	0.002	1	0	0
1	Left caudal anterior cingulate 1	126	Right posterior cingulate 1	0.002	0	0	1
Decreases							
67	Left thalamus proper 1	77	Left pallidum 2	−0.002	1	0	0
88	Right amygdala 5	155	Right inferior parietal 1	−0.002	0	1	0
63	Left transverse temporal 1	75	Left putamen 2	−0.003	1	0	0
16	Left isthmus cingulate 2	165	Right cuneus 1	−0.003	0	0	1
88	Right amygdala 5	98	Right caudate 3	−0.003	0	1	0
54	Left superior frontal 1	94	Right putamen 1	−0.003	0	0	1
84	Right amygdala 1	102	Right thalamus proper 1	−0.003	0	1	0
134	Right pars Triangularis 2	167	Right caudal middle frontal 1	−0.003	0	1	0
98	Right caudate 3	114	Right superior frontal 1	−0.003	0	1	0
83	Right Accumbens area 1	92	Right pallidum 1	−0.004	0	1	0
54	Left superior frontal 1	99	Right caudate 4	−0.004	0	0	1
38	Left Pericalcarine 1	153	Right isthmus cingulate 2	−0.004	0	0	1
154	Right inferior temporal 1	161	Right entorhinal 2	−0.004	0	1	0
89	Right amygdala 6	155	Right inferior parietal 1	−0.004	0	1	0
159	Right fusiform 2	160	Right entorhinal 1	−0.004	0	1	0
35	Left pars orbitalis 2	53	Left rostral middle frontal 3	−0.004	1	0	0
45	Left precentral 1	75	Left putamen 2	−0.004	1	0	0
126	Right posterior cingulate 1	152	Right isthmus cingulate 1	−0.004	0	1	0
89	Right amygdala 6	120	Right rostral anterior cingulate 2	−0.004	0	1	0
3	Left caudal middle frontal 1	45	Left precentral 1	−0.005	1	0	0
30	Left paracentral 1	70	Left caudate 2	−0.005	1	0	0
83	Right accumbens area 1	119	Right rostral anterior cingulate 1	−0.005	0	1	0
146	Right medial orbitofrontal 2	161	Right entorhinal 2	−0.005	0	1	0
58	Left superior temporal 1	72	Left caudate 4	−0.006	1	0	0
71	Left caudate 3	74	Left putamen 1	−0.006	1	0	0
49	Left rostral anterior cingulate 1	77	Left pallidum 2	−0.007	1	0	0
56	Left superior parietal 1	130	Right postcentral 3	−0.007	0	0	1
98	Right caudate 3	120	Right rostral anterior cingulate 2	−0.007	0	1	0
86	Right amygdala 3	146	Right medial orbitofrontal 2	−0.008	0	1	0
53	Left rostral middle frontal 3	76	Left pallidum 1	−0.008	1	0	0
142	Right parahippocampal 2	161	Right entorhinal 2	−0.008	0	1	0
84	Right amygdala 1	154	Right inferior temporal 1	−0.008	0	1	0
26	Left middle temporal 2	78	Left hippocampus 1	−0.009	1	0	0
85	Right amygdala 2	92	Right pallidum 1	−0.009	0	1	0
8	Left entorhinal 2	20	Left lateral orbitofrontal 2	−0.009	1	0	0
1	Left caudal anterior cingulate 1	15	Left isthmus cingulate 1	−0.009	1	0	0
46	Left precentral 2	115	Right superior frontal 2	−0.009	0	0	1
39	Left pericalcarine 2	131	Right pericalcarine 1	−0.011	0	0	1
37	Left pars triangularis 2	67	Left thalamus proper 1	−0.012	1	0	0
120	Right rostral anterior cingulate 2	148	Right lateral orbitofrontal 1	−0.013	0	1	0
111	Right superior temporal 2	129	Right postcentral 2	−0.013	0	1	0
26	Left middle temporal 2	81	Left amygdala 2	−0.014	1	0	0
67	Left thalamus proper 1	75	Left putamen 2	−0.014	1	0	0
132	Right pericalcarine 2	153	Right isthmus cingulate 2	−0.014	0	1	0
37	Left pars triangularis 2	70	Left caudate 2	−0.014	1	0	0
53	Left rostral middle frontal 3	70	Left caudate 2	−0.015	1	0	0
21	Left lingual 1	131	Right pericalcarine 1	−0.017	0	0	1
45	Left precentral 1	115	Right superior frontal 2	−0.018	0	0	1
86	Right amygdala 3	148	Right lateral orbitofrontal 1	−0.029	0	1	0
158	Right fusiform 1	163	Right entorhinal 4	−0.041	0	1	0
122	Right precuneus 2	166	Right cuneus 2	−0.052	0	1	0
30	Left paracentral 1	43	Left posterior cingulate 1	−0.057	1	0	0

### 3.7 Longitudinal analysis of topological properties

#### 3.7.1 Modularity

**FIGURE 8:**
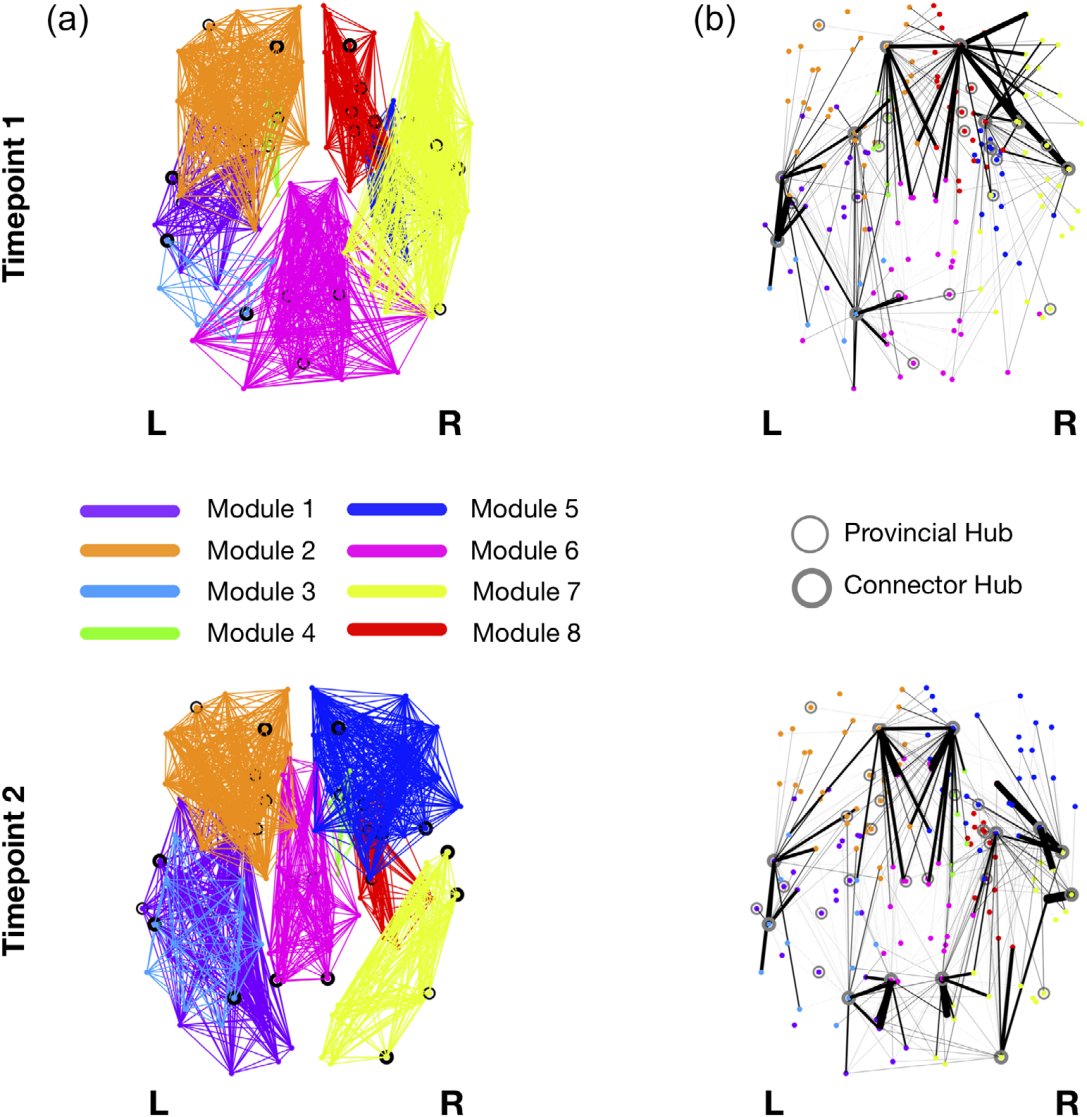
Modularity structure (a) and connector‐hub connectivity (b) at Timepoint 1 (top row) and Timepoint 2 (bottom row). Filled circles represent connector hubs and unfilled circles represent provincial hubs. The same number of modules was found at both timepoints but there were evident differences in modular arrangements (a) and in the undirected structural connectivity profile for the connector hubs (b). These differences are probably due to the higher number of connector hubs at the last timepoint. Giving the role of connector hubs in inter‐modular communication, the increase in their number between timepoints reflects an increase in integration of brain structural networks in aging

The optimal modularity structure had eight modules at both timepoints, and the two configurations had a similarity of 0.55. The arrangement of the modules has some differences between timepoints (Figure 8). Particularly, Module 1 at Timepoint 2 includes some occipital regions that are not included in the first timepoint, Module 4 changes from left to right hemisphere, Module 5 comprises frontal regions at Timepoint 2 that are not present at Timepoint 1, Module 6 loses occipital regions between timepoints, Module 7 shifts from frontal to occipital regions, and Module 8 also loses some frontal regions. Details of the regions belonging to each module are given in Table S3. Regarding the connector hubs' connectivity profiles, we observe differences between evaluations, with a higher number of connections in the posterior regions of the brain at Timepoint 2 (Figure 8).

#### 3.7.2 Hubs

**FIGURE 9:**
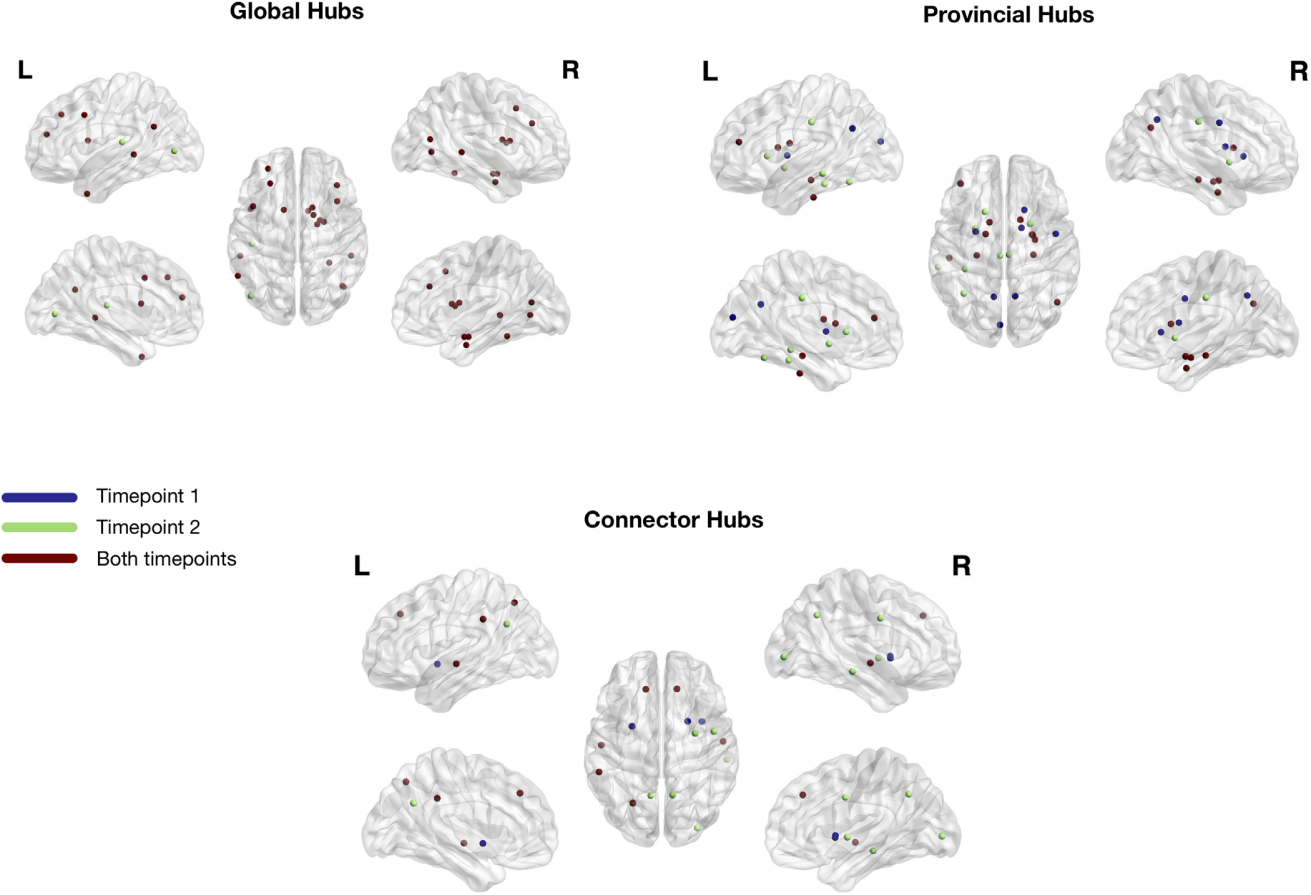
Hubs (global, provincial, and connector) identified in the two timepoints. Blue represents hubs only identified at Timepoint 1, green represents hubs only identified at Timepoint 2, and red represents hubs identified at both timepoints. We observe an increase in all type of hubs (global, provincial, and connector) between timepoints. Furthermore, some hubs change their role between timepoints (from provincial to connector—left precuneus 2, right precuneus 2, and right precentral 1; and from connector to provincial—left putamen 2 and right putamen 1)

Global hubs were defined as regions with high normalized nodal efficiency. In the first timepoint, 19 regions were identified as hubs, while at Timepoint 2, two additional regions were classified as hubs, namely left lateral occipital 1 (nomenclature of anatomical parcels is done according to: [hemisphere region subdivision]) and left transverse temporal 1 (Table 4; Figure 9).

**TABLE 4:** Global hubs of the brain for the two timepoints

Global hubs	
M1	M2
Left rostral middle frontal 1	Left rostral middle frontal 1
Left rostral middle frontal 2	Left rostral middle frontal 2
Right amygdala 2	Right lateral occipital 1
Right lateral occipital 1	Left caudate 1
Left caudate 1	Right inferior parietal 2
Right caudate 1	Right amygdala 2
Right middle temporal 2	Right middle temporal 2
Right inferior parietal 2	Left caudal middle frontal 1
Right caudate 6	Left middle temporal 2
Left caudal middle frontal 1	Right caudate 5
Left supramarginal 2	Right caudal middle frontal 1
Right rostral middle frontal 2	Right caudate 1
Right caudal middle frontal 1	Right rostral middle frontal 2
Right caudate 5	Right amygdala 1
Left middle temporal 2	Left supramarginal 2
Right amygdala 3	Right amygdala 3
Right amygdala 1	Right caudate 6
Left middle temporal 3	Left transverse temporal 1
Right fusiform 1	Left lateral occipital 1
	Left middle temporal 3
	Right fusiform 1

*Note*: Hubs are sorted by nodal efficiency.

Regarding provincial hubs, which play a key role in intra‐modular communication, 18 hubs were detected at the first timepoint and 19 at the last timepoint (Table 5). Left cuneus 2, left precuneus 2, left putamen 1, right caudate 1, right caudate 2, right precuneus 2, and right precentral 1 were only detected at Timepoint 1, while left fusiform 1, left fusiform 2, left middle temporal 1, left posterior cingulate 2, left caudate 5, left putamen 2, right putamen 1, and right posterior cingulate 2 were only detected at Timepoint 2. The rest of the regions were common to both timepoints (Figure 9).

**TABLE 5:** Provincial and connector hubs for the two timepoints

Provincial hubs	Connector hubs		
M1	M2	M1	M2
Right precuneus 2	Right putamen 1	Left superior frontal 2	Right superior frontal 3
Right precentral 1	Right inferior parietal 1	Right superior temporal 2	Left superior frontal 2
Right caudate 6	Right posterior cingulate 2	Right superior frontal 3	Left supramarginal 1
Left precuneus 2	Right caudate 6	Left superior temporal 2	Left superior temporal 2
Left rostral middle frontal 3	Left rostral middle frontal 3	Left putamen 2	Right precuneus 2
Right hippocampus 2	Right hippocampus 2	Left superior parietal 2	Left superior parietal 2
Right inferior parietal 1	Right amygdala 1	Right putamen 1	Right superior temporal 2
Right amygdala 5	Left posterior cingulate 2	Right insula 2	Right lateral occipital 2
Right amygdala 1	Right amygdala 5	Left supramarginal 1	Right precentral 1
Left hippocampus 2	Left putamen 2		Right middle temporal 1
Left cuneus 2	Left hippocampus 2		Right putamen 2
Right amygdala 4	Right amygdala 4		Left precuneus 2
Left putamen 1	Left fusiform 2		
Left inferior temporal 2	Left caudate 1		
Left caudate 1	Left inferior temporal 2		
Right caudate 1	Left caudate 2		
Left caudate 2	Left caudate 5		
Right caudate 2	Left fusiform 1		
	Left middle temporal 1		

*Note*: Hubs are sorted by modularity degree *z*‐score.

In the case of connector hubs, which have a central role in inter‐modular communication, 9 hubs were detected at Timepoint 1 and 12 at Timepoint 2 (Table 5).

Left putamen 2, right putamen 1 and right insula 2 were only detected at timepoint 1, while left precuneus 2, right putamen 2, right precuneus 2, right precentral 1, right middle temporal 1, and right lateral occipital 2 was only detected at Timepoint 2 (Figure 9).

Interestingly, left putamen 2 and right putamen 2 lost their connector hub status between timepoints but they were identified as provincial hubs at Timepoint 2. In the opposite direction, left precuneus 2, right precuneus 2, and right precentral 1 lost its provincial hub status and were identified as a connector hub at Timepoint 2. Furthermore, left caudate 1, right amygdala 1, right caudate 1, and right caudate 6 were identified as global and provincial hubs at both timepoints.

#### 3.7.3 Fingerprints of modular connectivity

**FIGURE 10:**
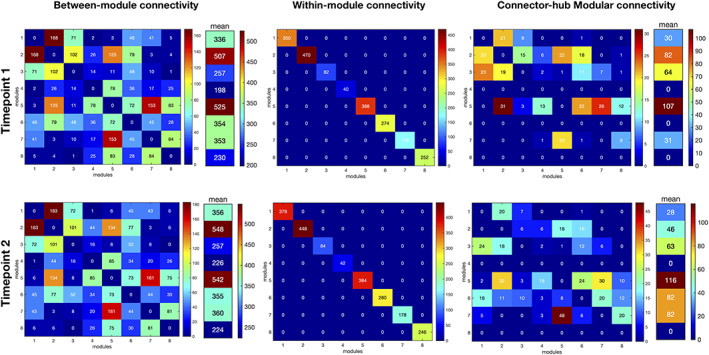
Fingerprints of modular connectivity at Timepoint 1 (top row) and Timepoint 2 (bottom row). Left column represents the inter‐modular connectivity, middle column the intra‐module connectivity, and right column the connector‐hub driven inter‐modular connectivity. Modular connectivity strength is quantified as the total number of connections (degree) of all nodes forming a module. Community structure of Timepoint 2 was selected as the reference scheme, since it had higher group goodness‐of‐fit. We observe different patterns only in connector‐hub driven inter‐modular connectivity. Overall, there was an increase of around 33% in this connectivity between timepoints, which is probably due to the increase in the number of connector hub. This results again suggests an increase in integration of brain SC during aging

The reference scheme chosen to analyze fingerprints of modular connectivity was the community structure of Timepoint 2. Connector‐hub‐driven inter‐modular connectivity had significant alterations between timepoints (Figure 10). Overall, there was an increase of around 33% in modular connectivity strength in the second timepoint. Specifically, we found increased connectivity from Module 7 (right hemisphere; temporal, parietal, and occipital regions) to Modules 1 (left hemisphere; entorhinal, hippocampus, amygdala, parahippocampal, temporal, and occipital regions), 5 (right hemisphere; accumbens area, pallidum, putamen, caudate, insula, cingulate, and frontal regions), and 8 (right hemisphere; amygdala, hippocampus, parahippocampal, entorhinal, fusiform, and temporal regions). Also, Module 6 (bilateral cingulate cortex regions, bilateral paracentral, bilateral precuneus, and right postcentral) had no connectivity with any other module at Timepoint 1, but at Timepoint 2, there was connectivity between Module 6 and the other modules. Connectivity between Module 2 (left hemisphere; caudate, putamen, pallidum, accumbens area, thalamus, insula, rostral anterior cingulate, and frontal regions) and Modules 1 and 3 (left hemisphere; inferior and superior parietal, postcentral, precentral, and supramarginal) decreased between timepoints. Of notice, at Timepoint 1, Module 2 had two connector hubs (left superior frontal 2 and left putamen 2), Module 6 had none and Module 7 had one (right superior temporal 2), while at Timepoint 2, Module 2 had one connector hub (left superior frontal 2), Module 6 had two (left precuneus 2 and right precuneus 2), and Module 7 had three (right superior temporal 2, right middle temporal 1, and right lateral occipital 2). Modules 4 and 8 had no connector hubs at both timepoints. No differences were found for intra‐modular and inter‐modular connectivity.

## 4 DISCUSSION

In this study, we developed a new CBP method, based on diffusion MRI data. We evaluated different clustering algorithms in conjunction with different dimensionality reduction techniques and chose the best performing method. *K*‐means clustering combined with SOM was the selected method due to its higher silhouette coefficient and resulting clusters with more evenly distributed sizes. Previous studies have used *k*‐means clustering, making it the most popular clustering algorithm amongst CBP works, so its appropriateness for CBP has already been validated (Abivardi & Bach, 2017; Anwander et al., 2007; Bach, Behrens, Garrido, Weiskopf, & Dolan, 2011; J. C. Klein et al., 2007; Reuter et al., 2020). We also demonstrated that SOMs are a suitable method for dimensionality reduction that can be applied prior to clustering. To date, this technique has only been used in one study performing CBP of functional data (Mishra et al., 2014). Here we show evidence that pre‐clustering dimensionality reduction with SOM presents a valid and recommended solution for CBP methods which preserves the topographic organization of the input data (Kohonen, 1982, 1990).

Moreover, we developed a new metric to evaluate the estimated CBP, specifically, to verify if the goal of grouping voxels with similar connectivity profiles was successfully accomplished—connectivity homogeneity fingerprint. This novel metric takes into account the connections of each voxel inside a region, with higher values representing a region with more homogeneous signatures of structural connectivity across all of its voxels (i.e., most of the region's voxels are connected to the same brain regions). So far, many techniques to evaluate the quality of a parcellation have been proposed. Yet, choosing the most suitable evaluation method is a challenging task due to the lack of a ground truth. The existing methods either evaluate the reproducibility (i.e., the alignment between different parcellations from different subjects or different acquisitions of the same subject), quality of clustering solutions (i.e., the similarity of voxels grouped in the same cluster), agreement with cytoarchitecture, task fMRI activation and myelination or impact on network analysis (Arslan et al., 2018). One of the metrics developed to evaluate the quality of clustering solutions is the functional homogeneity: the average Pearson's correlation coefficient between the connectivity fingerprints of each pair of voxels inside a cluster (Chong et al., 2017; Kong et al., 2019; Schaefer et al., 2018). The metric we propose here (CHF) does not consider the connectivity weights directly which can lead to lower homogeneity values, even if a pair of voxels is connected to the same brain regions but with different connectivity strengths. Thus, the CHF reflects a more adequate measure of regional homogeneity, which evaluates the strength or consistency level of a region's connectivity fingerprint to the rest of the brain, thus representing a more complete and robust approach to evaluating a parcellation. Furthermore, the previous existing homogeneity measure was designed specifically for functional data, thus limiting its extension to structural data. Additionally, our metric also takes into account the size of the seed region, by estimating the proportion of seed voxels that are connected to each target, thus the CHF will not be biased by the region size. Our results from CHF analysis validate the CBP method developed here, since all the parcellations exhibit higher homogeneity values in comparison to random parcellations and to the original partition. In the comparison with the null model, a few regions exhibited higher homogeneity for the random parcellations. These correspond to small clusters and so the probability that they are being rotated into regions with high connectivity homogeneity is higher and we assume this is the cause for the higher CHF for the null model in these clusters. However, future work should confirm that this is the case, otherwise these regions could be merged with others in the final parcellation. Furthermore, in addition to apply our CBP method to an aging cohort, we also applied it to an HCP dataset with ultra‐high *b*‐value diffusion MRI. The results were highly convergent between the two cohorts, which demonstrates the robustness of our method. Moreover, when one of the generated parcellations was applied to the data of a different timepoint (for the same cohort), the homogeneity values were very similar to the baseline, which demonstrates its appropriateness for longitudinal analysis.

Regarding longitudinal changes in structural connectivity, our results revealed a significant sub‐network with both decreases and increases in WM structural connectivity along time. Increases in connectivity were mainly composed by inter‐hemispheric connections, while decreases occurred mostly in intra‐hemispheric connections. These results are in accordance with the “last‐in, first‐out” hypothesis, which states that regions developing later are more prone to age‐related decline (Raz, 2000). This theory has been supported by DTI studies investigating white matter microstructural properties, which report steepest declines for association fibers (i.e., fibers connecting regions of the same hemisphere) in comparison to commissural fibers (i.e., fibers crossing hemispheres).

The longitudinal analysis of topological features of brain WM structural networks also revealed some alterations. Concerning nodal efficiency and the topological roles of nodes (provincial and connector), there was an increase in the number of hubs (global, provincial, and connector) from the first to last timepoint. Interestingly, left caudate 1, right amygdala 1, and right caudate 6 were consistently identified as both global and provincial hubs in all timepoints. The caudate nuclei are involved in different cognitive dimensions, such as, motor and action planning, decision making, motivation, and reward processing (Bick et al., 2019; Cera, Esposito, Cieri, & Tartaro, 2019; Grahn, Parkinson, & Owen, 2008; Wilson et al., 2018). Previous studies found significant atrophy of the caudate along aging (Hoffstaedter et al., 2015; Raz et al., 2003). Interestingly, we identified as hubs three clusters in the right caudate and only one in the left caudate and there is one study reporting a longitudinal rightward lateralization of the caudate volume in older adults (Esteves et al., 2019). The amygdala has been associated with emotion processing of both fearful and rewarding stimuli. It is also known to modulate memory and attention for emotional stimuli and to be involved in positive affect and motivation (Gallagher & Chiba, 1996; Janak & Tye, 2015; Mather, 2016; Sah, Faber, Lopez de Armentia, & Power, 2003; Salzman & Fusi, 2010). Past studies report relative preservation of both structure and function of the amygdala in normal aging (Good et al., 2001; Mather, 2016; Nashiro, Sakaki, & Mather, 2012). Alongside with this, emotional processing also appears to be spared in aging (Nashiro et al., 2012). Our results align with these findings, since the amygdala maintains its importance in the brain structural network along time, both in terms of nodal efficiency and intra‐modular communication.

Two clusters (left putamen 2 and right putamen 1) lost their role as connector hubs and were identified as provincial hubs. This means that the participation coefficient of these clusters was lower and thus they established more connections with regions belonging to the same module than with regions outside their own module and lost their importance in integrating different regions of the brain. The putamina are involved in different cognitive functions, such as reinforcement learning and motor control, language and processing of sensory, and motor aspects of pain (Haber, 2016; Starr et al., 2011; Vinas‐Guasch & Wu, 2017). Studies investigating age effects on this region described significant atrophy (Fjell & Walhovd, 2010; Jancke, Merillat, Liem, & Hanggi, 2015; Raz et al., 2003) and also microstructural damage (Cherubini, Peran, Caltagirone, Sabatini, & Spalletta, 2009) of these subcortical nuclei, which might explain why the putamen lost its connector hub role in the brain structural network. On the opposite direction, right precuneus 2 and right precentral 1 changed from provincial to connector hubs. Precuneus plays an important role in multiple higher‐order cognitive functions, such as visuo‐spatial imagery, episodic memory retrieval, self‐processing, and consciousness (Cavanna & Trimble, 2006). Some aging studies report a relative preservation of precuneus' cortical thickness in comparison to other regions (Fjell et al., 2009; Lee et al., 2018). In line with our findings, Gong et al. (2009) found that the precuneus was consistently identified as a hub independent of age in white matter structural networks. Precentral gyrus is known to be involved in motor performance (Picard & Strick, 2003; Porro et al., 1996; Ribas, 2010; Yousry et al., 1997) but some studies also report a role of this region in emotion processing (de Gelder, Snyder, Greve, Gerard, & Hadjikhani, 2004; Hajcak et al., 2007; Hardee et al., 2017; Mazzola et al., 2013; Saarimaki et al., 2016). Although aging studies report significant atrophy of this region (Salat et al., 2004; Thambisetty et al., 2010), one recent fMRI study using graph theory analysis described increased degree centrality (i.e., a measure of the importance of the node in the network) of precentral gyrus in both cognitively normal older adults and subjects with mild cognitive impairment (MCI) despite existing volume declines (Behfar et al., 2020). Furthermore, Behfar and colleagues showed that the increased degree centrality was correlated with better scores of cognitive performance in the MCI group, which might represent a compensatory mechanism. Our results align with these findings, since the precentral gyrus increased its importance in the network along time, by moving from having a role only in intra‐modular communication to have the function of establishing communication with different modules of the brain and thus being important for functional integration.

Concerning modularity structure, the same number of modules was identified at the two timepoints but some differences in the modules' configuration were found. In terms of fingerprints of modular connectivity, there was a longitudinal increase in connector‐hub driven inter‐modular connectivity, which could be driven by the increase in the number of detected connector hubs at Timepoint 2. This result suggests an increase of brain structural networks' integration during aging. Some past fMRI studies report increased integration along aging (Cao et al., 2014; He, Wang, Zhuang, & Qiu, 2020). However, a recent study exploring white matter structural connectivity report decreased integration with normal aging (Puxeddu et al., 2020). Our result could be attributed to the higher resolution of our parcellation, which may have allowed the identification of additional connector hubs, that at lower resolutions would not be identified.

This study has some limitations, namely the use of a 1.5‐T MRI scanner. While we recognize that this limitation may inevitably influence to some extent our results (changes in network connectivity, hubs, and modularity structure), we believe that its effects are minimized by the fact that we compared two evaluations/timepoints sharing the same neuroimaging acquisition and preprocessing protocol (same scanner, acquisition parameters, and data processing pipeline). Another limitation concerns the criterion to set the maximum number of clusters. While we demonstrated its suitability for the datasets included in this study, future work must investigate and potentially further optimize this criterion in order to assure its robustness when applied to different datasets.

In summary, we present a new method to create a CBP of the human brain based on white matter structural connectivity data which has accomplished the main goal of grouping voxels with similar connectivity profiles. Additionally, we propose a new metric (connectivity homogeneity fingerprint) to evaluate the quality of a parcellation by computing the consistency level of regional connectivity fingerprints, with potential for application to other types of neuroimaging data. Furthermore, we applied the derived parcellation to explore longitudinal changes in structural networks of an aging cohort and found signatures of brain's reorganization along aging. Particularly, we found decreases in intra‐hemispheric connectivity and increases in inter‐hemispheric connectivity, which supports the “last‐in, first‐out” hypothesis and a rearrangement in the topological roles of the nodes in the network. We also found evidence of increased integration, which was not observed in previous studies, but it can be explained by the higher resolution of our parcellation which allowed the identification of more connector hubs. Taken together, our study proposes a novel and robust solution for performing and evaluating CBP of the human brain. With potential for application to any whole‐brain DTI‐based cohort, here we show its potential appropriateness by characterizing the longitudinal changes of the structural connectome in aging, which were highly consistent with the existing literature. As future work, a comparison between the results obtained in the analysis of age‐related longitudinal changes of the structural networks using the CBP method and using the original parcellation (DKT40 and Buckner 40) could help understand if the developed CBP unraveled new findings regarding the aging brain and thus prove some of its advantages over conventional methods.

## CONFLICT OF INTERESTS

The authors declare that they have no conflict of interests.

## AUTHOR CONTRIBUTIONS

Nuno Sousa, Ana Coelho, and Henrique M. Fernandes conceived the study. Liliana Amorim, Teresa Castanho, Carlos Portugal‐Nunes, Nadine Correia Santos performed participants' recruitment. Liliana Amorim and Teresa Castanho performed the neurocognitive assessments. Ricardo Magalhães, Pedro S. Moreira, and Ana Coelho performed the MRI acquisitions. Ana Coelho performed the MRI data pre‐processing. Ana Coelho and Henrique M. Fernandes performed the data analysis and designed/developed the proposed methodological framework. Ana Coelho wrote the first draft of the manuscript and all authors contributed for the following and final versions of the manuscript.

## ETHICS STATEMENT

All procedures followed were in accordance with the ethical standards of the responsible committee on human experimentation (institutional and national) and with the Helsinki Declaration of 1975, and the applicable revisions at the time of the investigation. Informed consent was obtained from all patients for being included in the study.

## Supporting information


**Appendix S1**: Supporting Information

## Data Availability

The datasets generated during and/or analyzed during the current study are available from the corresponding author on reasonable request.
