# Supplementary material for: A novel method for estimating connectivity‐based parcellation of the human brain from diffusion MRI: Application to an aging cohort
Source: Hum Brain Mapp. 2022 Mar 11;43(8):2419–43. doi: 10.1002/hbm.25773 (PMC9057102; doi:10.1002/hbm.25773)
Supplement: Supplementary file 1 — Appendix S1: Supporting Information [file HBM-43-2419-s001.docx]

**A novel method for estimating connectivity-based parcellation of the human brain from diffusion MRI: application to an aging cohort**

Ana Coelho^1,2,3^, Ricardo Magalhães^1,2,3^, Pedro S. Moreira^1,2,3^, Liliana Amorim^1,2,3^, Carlos Portugal-Nunes^1,2,3^, Teresa Castanho^1,2,3^, Nadine Correia Santos^1,2,3^, Nuno Sousa^1,2,3#^, Henrique M. Fernandes^4,5#^

^1^Life and Health Sciences Research Institute (ICVS), School of Medicine, University of Minho, 4710-057 Braga, Portugal.

^2^ICVS/3B’s, PT Government Associate Laboratory, 4710-057 Braga/Guimarães, Portugal.

^3^Clinical Academic Center – Braga, 4710-057 Braga, Portugal.

^4^Center for Music in the Brain (MIB), Aarhus University, Aarhus, Denmark

^5^Department of Psychiatry, University of Oxford, Oxford, UK

# - Corresponding authors and equal contribution

**Author Note**

Correspondence concerning this article should be addressed to Nuno Sousa, Life and Health Sciences Research Institute (ICVS), School of Medicine, University of Minho, Campus Gualtar, 4710-057 Braga, Portugal. Tel: +351 253 604878. Fax: +351 253 604809. Email: [njcsousa@med.uminho.pt](mailto:njcsousa@med.uminho.pt)

**Supplementary Information**

*Graph Theory Metrics*

The following graph theory metrics were computed in this study.

*Modularity.* Modularity quantifies the degree to which nodes of a network may aggregate into densely connected non-overlapping modules or communities (Fornito et al., 2016). Nodes within a community are more strongly connected with each other than with nodes outside this community. Thus, the optimal community structure will be the partition of the network that maximizes intra-module connectivity and minimizes inter-module connectivity. The index of modularity, Q, is given by the difference between the empirical degree of intra-module connectivity and the degree expected by chance (Fornito et al., 2016). The optimal community structure can be found by searching for the partition that maximizes Q. One popular algorithm used to find the optimal partition is the Louvain algorithm and, shortly, this is how it works: first, it starts with all nodes in a distinct module, then it chooses a node at random and merges it with the module that produces the largest gain in Q, these steps are repeated until no additional gains in Q are possible (Blondel et al., 2008). Given that at each iteration, nodes are chosen randomly, running the algorithm multiple times can lead to different solutions. Also, another limitation is the so-called degeneracy problem, that can cause the existence of large number of different solutions, since there is not a clear global maximum of Q (Good et al., 2010). To circumvent this problem, we ran the Louvain algorithm 10000 times and selected the partition having the higher number of occurrences in the set of 10000 partitions, i.e., the partition that was more consistent. To compare the optimal community structures found at each timepoint, we defined a similarity metric. For each module in a partition, we found the module in the other partition that was more similar to this one (by finding the maximum of the number of shared regions divided by the total number of regions in the two modules). The similarity metric was then calculated as the mean of the maximum values for each module. Values close to 1 indicate higher similarity between the partitions.

*Hubs.* Hubs can be defined as nodes with high regional efficiency ($E_{nodal}$) (Achard & Bullmore, 2007). This measure reflects how well a node is integrated within the network via its shortest paths and is defined as the mean of the inverse shortest path length, $l_{ij}$, between a given node and all other nodes in the network:

$$E_{nodal}(j)=\frac{1}{(N-1)}\sum_{i} \frac{1}{l_{ij}}$$

If the normalized $E_{nodal}$ (divided by the mean $E_{nodal}$ of all nodes) is larger than the normalized mean $E_{nodal}$ of all nodes of the network plus one standard deviation (SD), the node is considered a hub (Lo et al., 2010).

Furthermore, we analyzed the topological roles of nodes in the communication within and between modules. This allowed the classification of nodes into provincial and connector hubs. The definition of these roles is described below.

*Provincial Hubs* are nodes with high within-module degree z-score (greater than the mean plus SD of all nodes) and low participation coefficient (PC $\leq$ 0.3). Positive values of within-module degree z-score indicate high (above the average) intra-module connectivity, and thus higher values of this measure suggest that the node plays a central role in intra-modular communication. Participation coefficient (PC) compares the number of connections of a node with other nodes in different modules, to the total number of connections to other nodes in the same module. Values close to one indicate that the edges of a node are distributed uniformly across modules while a value of zero means that all edges of a nodes are limited to its own module. Thus, provincial hubs are characterized by comprising most of their connections within their own module (Fornito et al., 2016).

*Connector Hubs* were also defined as nodes with high within-module degree z-score and high participation coefficient (PC > 0.3). This means they have many connections with other modules, and thus play a key role in inter-modular communication (Fornito et al., 2016).

**
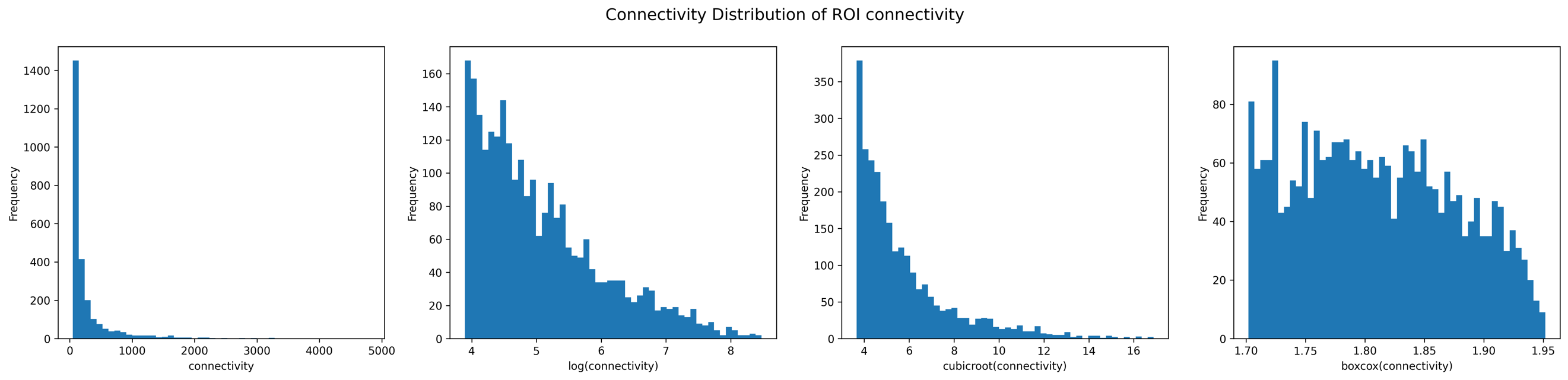
Supplementary Figures**

**Figure S1 –** Example of the distribution of structural connectivity values for a region and the different transforms applied to normalize values. The BoxCox transform is the one achieving a distribution more approximated to a normal distribution.


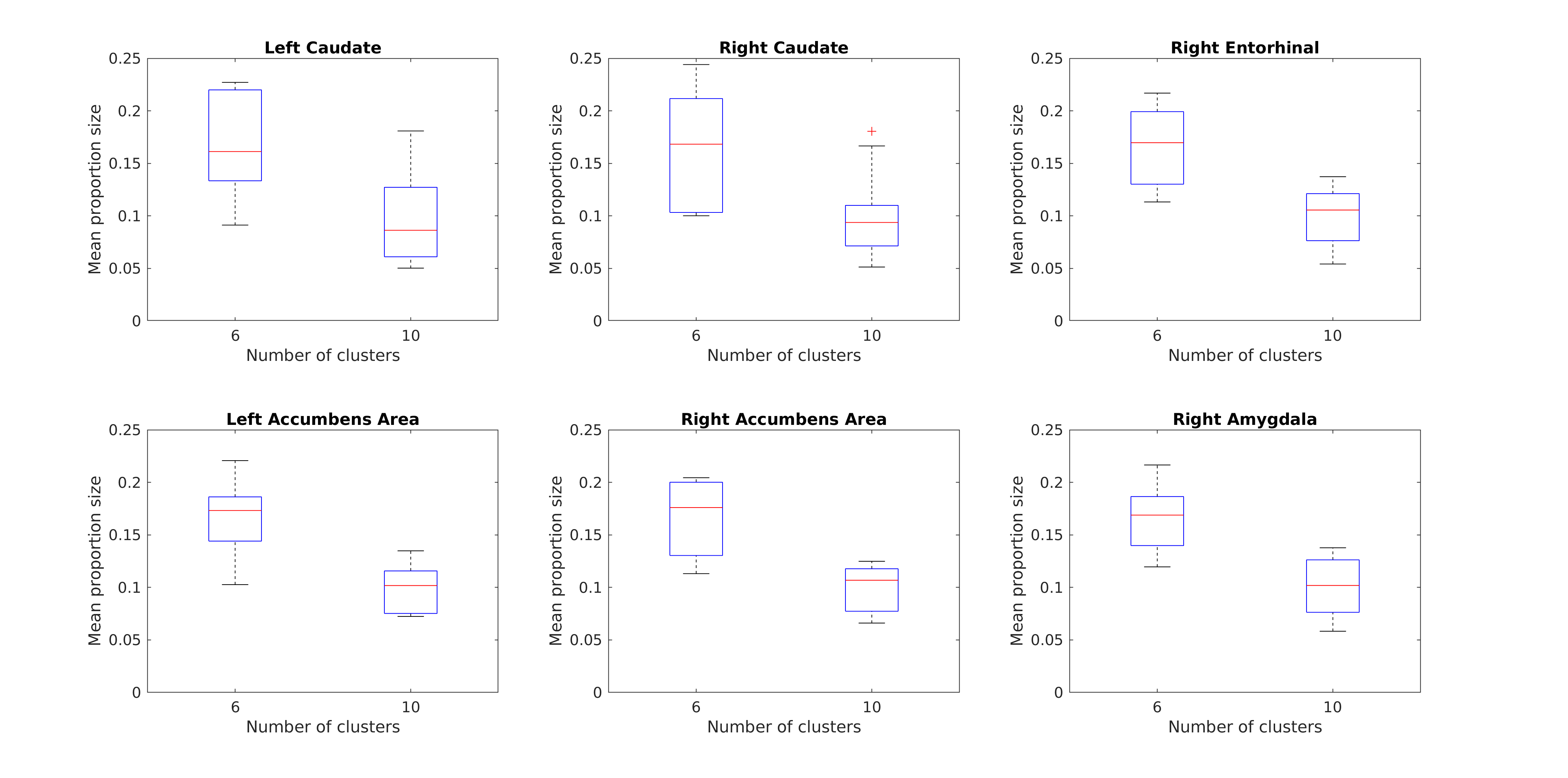


**Figure S2 -** Mean proportion size (i.e., size of clusters divided by the size of the original region) for the k=6 and k=10 solutions for regions subdivided in 6 clusters according to the silhouette score.

**Figure S3 –** Hopkins statistic for each region of the SWITCHBOX cohort. Top row represents regions of the DKT40 parcellation, bottom row represents regions of the generated Silhouette parcellation. Red line represents the threshold for rejecting the null hypothesis, where values lower than this threshold indicate that we can reject the null hypothesis that the data originates from a uniform distribution. All regions present values lower than 0.5, thus they present non uniform data.


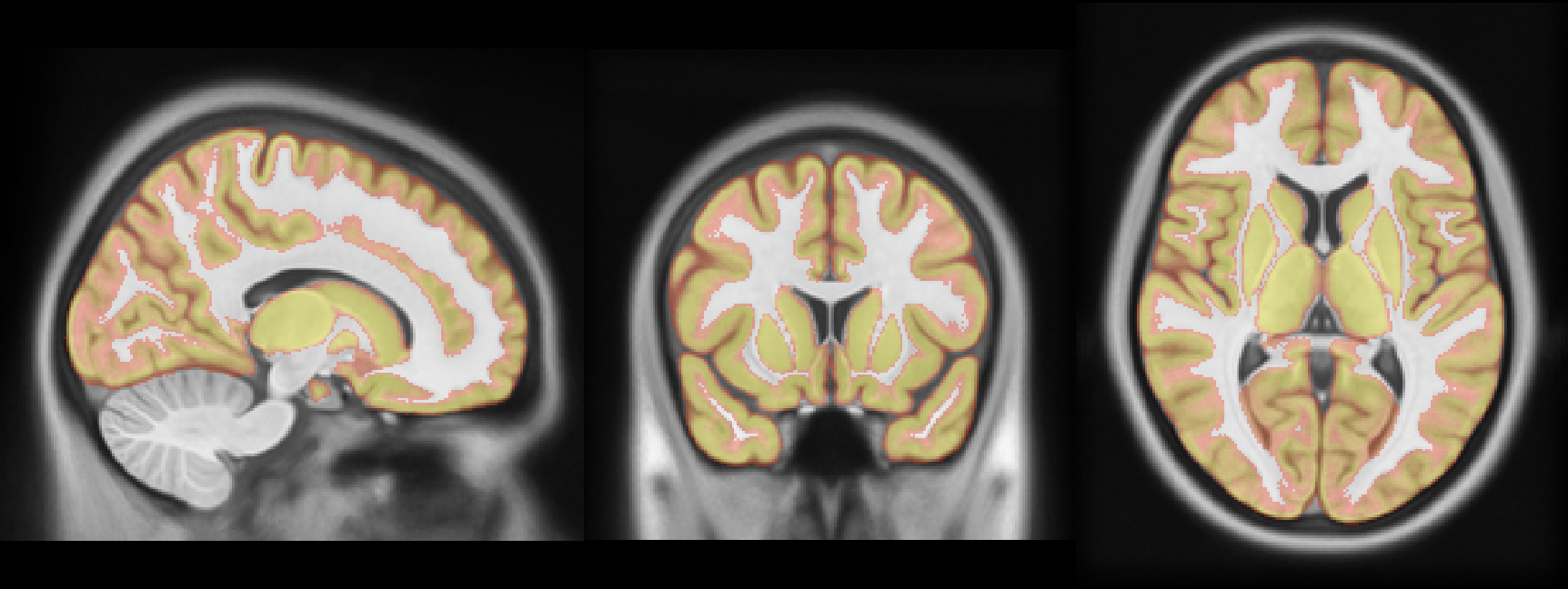

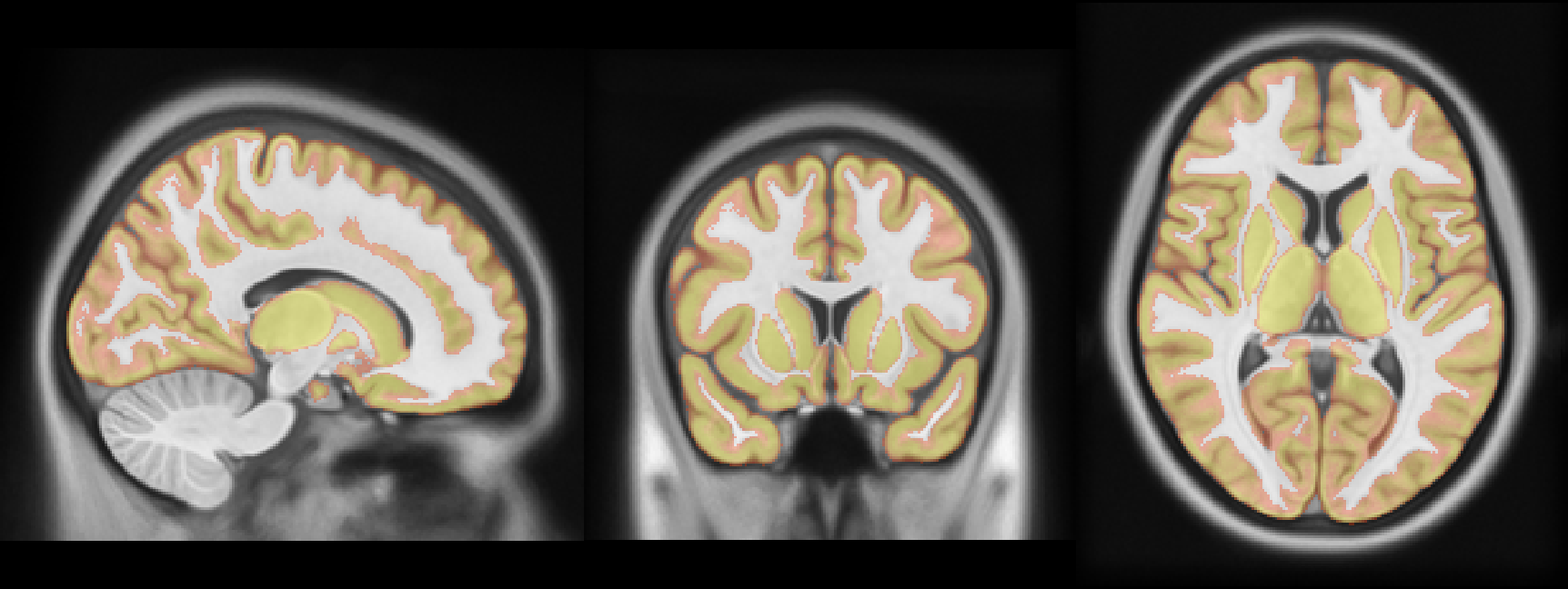

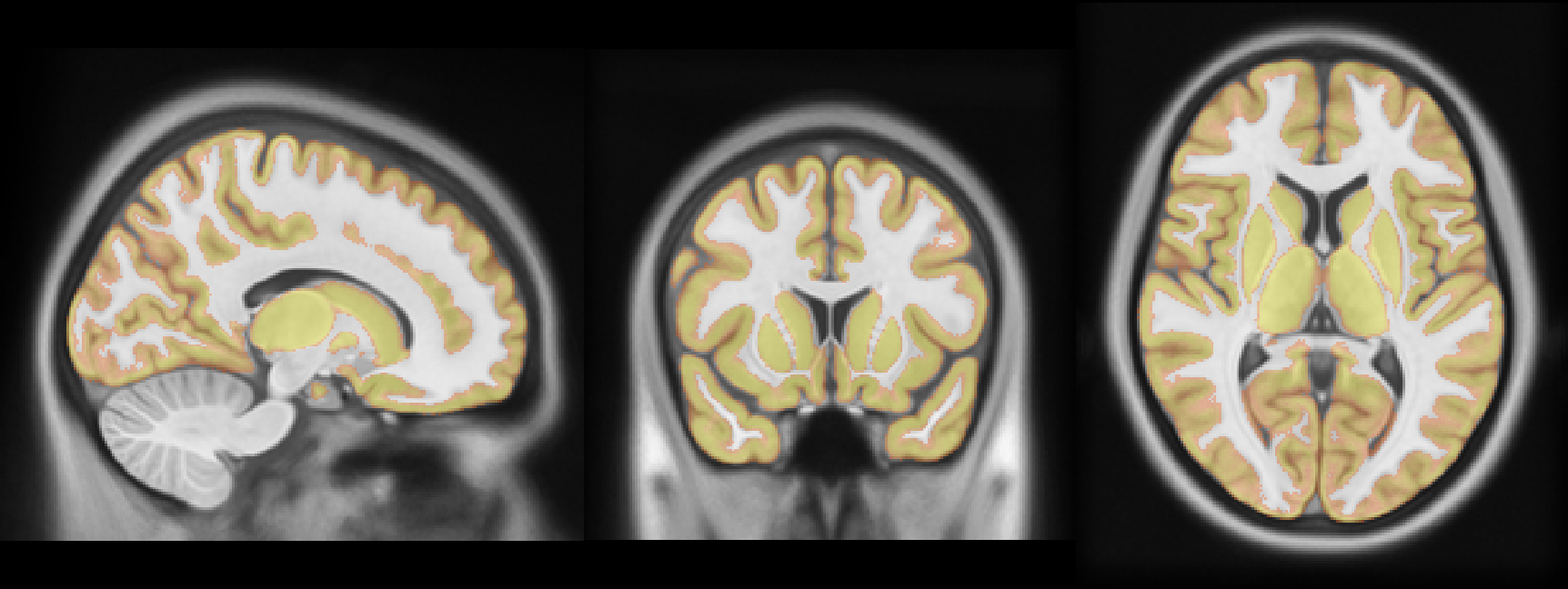

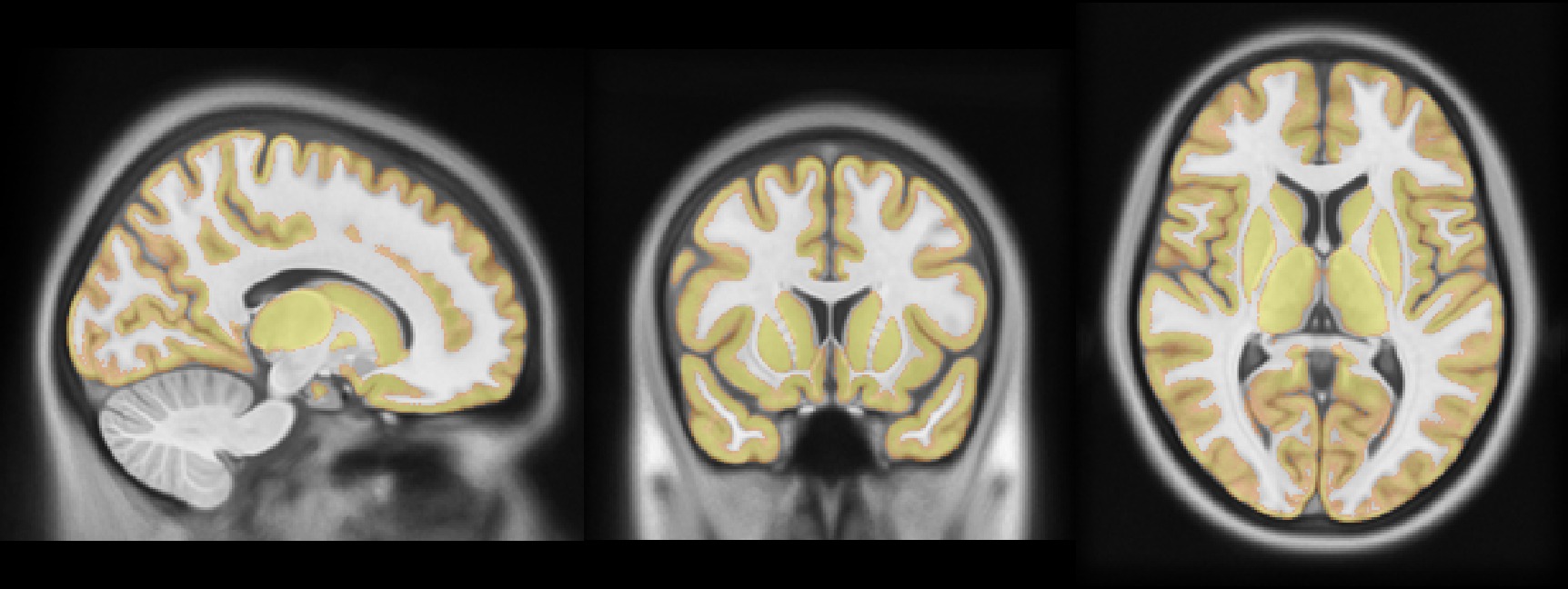


**10%**

**20%**

**30%**

**40%**

**Figure S3** - Group probability map of DKT40 template with different density thresholds. Threshold represents a percentage of the total number of subjects. We selected the 20% threshold as it had a better coverage of grey matter.


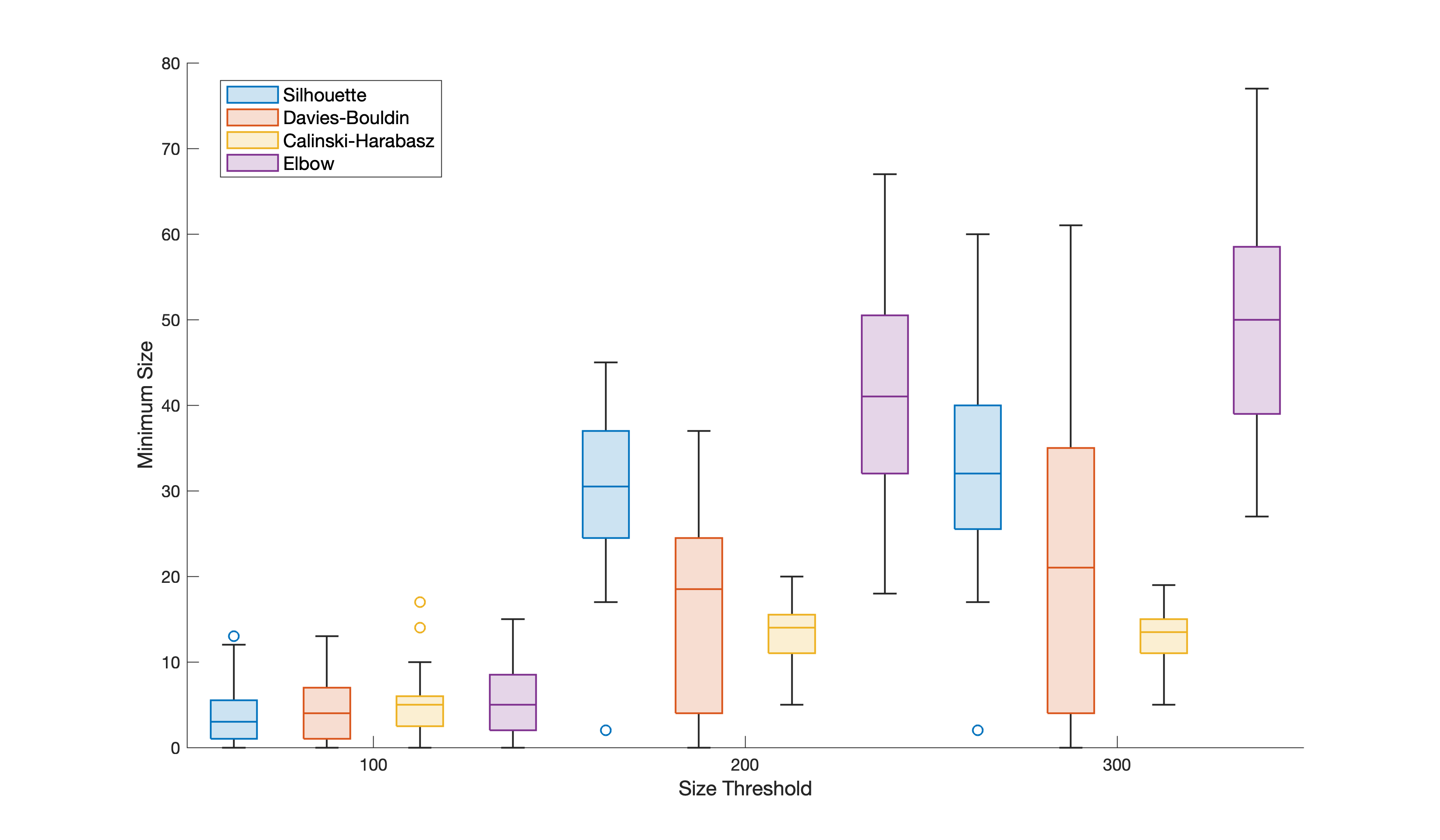


**Figure S4 –** Plot with the minimum cluster size of all subjects for each threshold and each metric: A) SWITCHBOX dataset, B) MGH-HCP dataset. In the SWITCHBOX dataset, the threshold of 300 voxels gives a minimum cluster size above 5 voxels for all metrics. In the MGH-HCP dataset, the threshold of 200 voxels was selected.


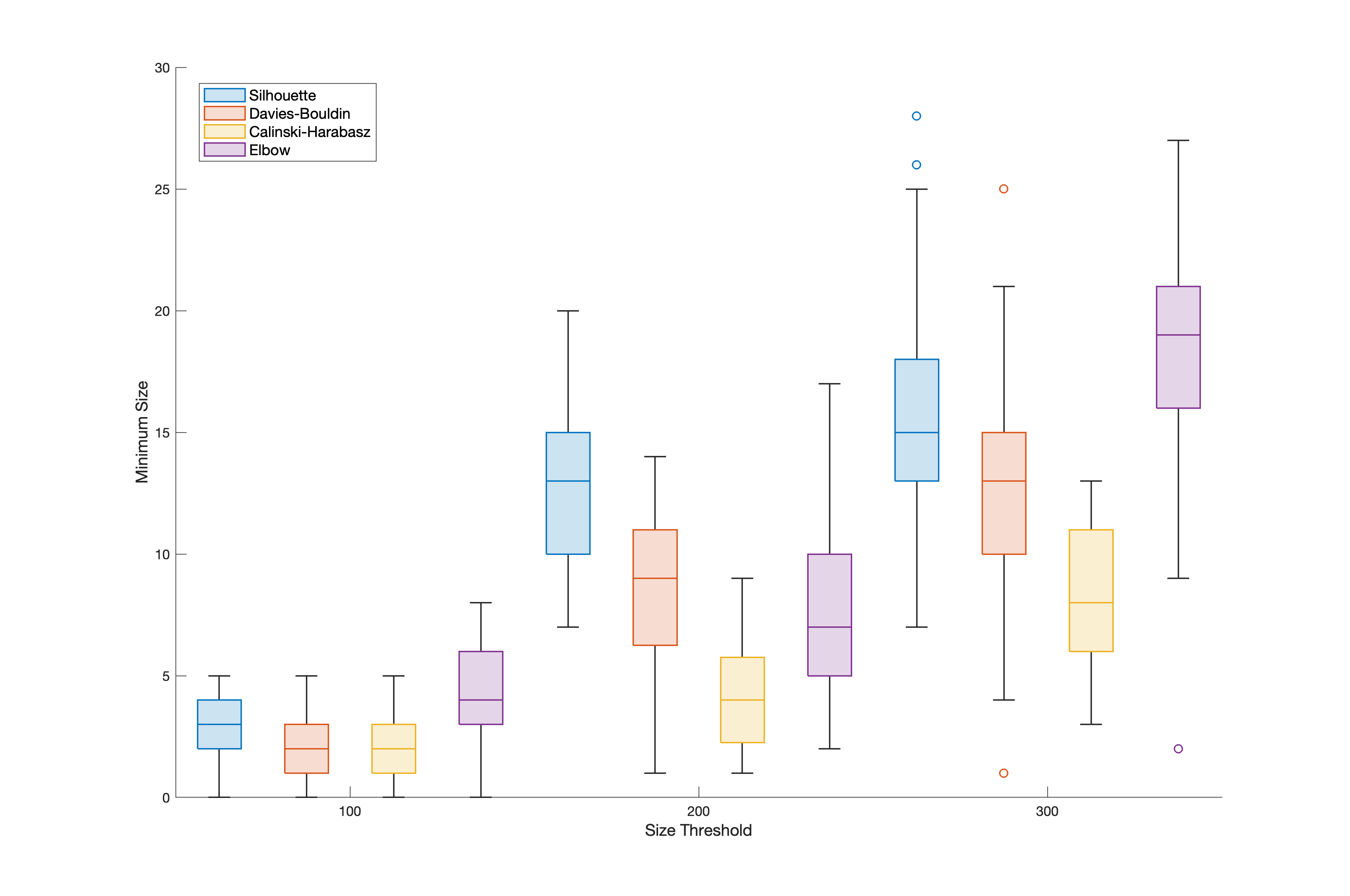


**A.**

**B.**


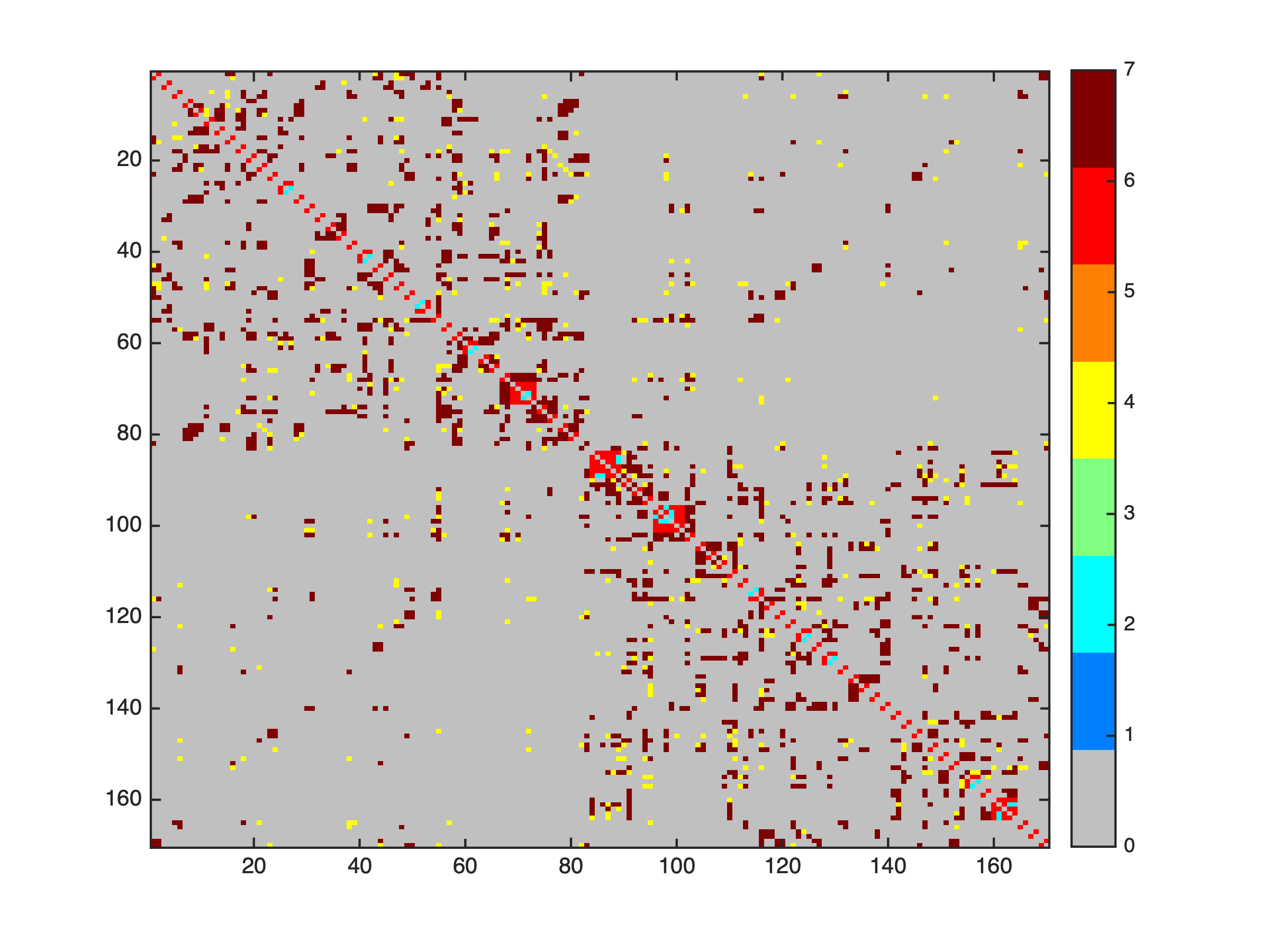


Node

Node

**Figure S5 –** Structural connections surviving the different methods accounting for intra-cluster connectivity. 1 represents connections with intra-cluster connectivity set to 0; 2 represents connections with intra-cluster connectivity set to 1; 3 represents connections with original intra-cluster connectivity values; 4 represents connections common to intra-cluster connectivity set to 0 and 1; 5 represents connections common to intra-cluster connectivity set to 0 and original values; 6 represents connections common to intra-cluster connectivity set to 1 and original values; 7 represents connections common to the three strategies.


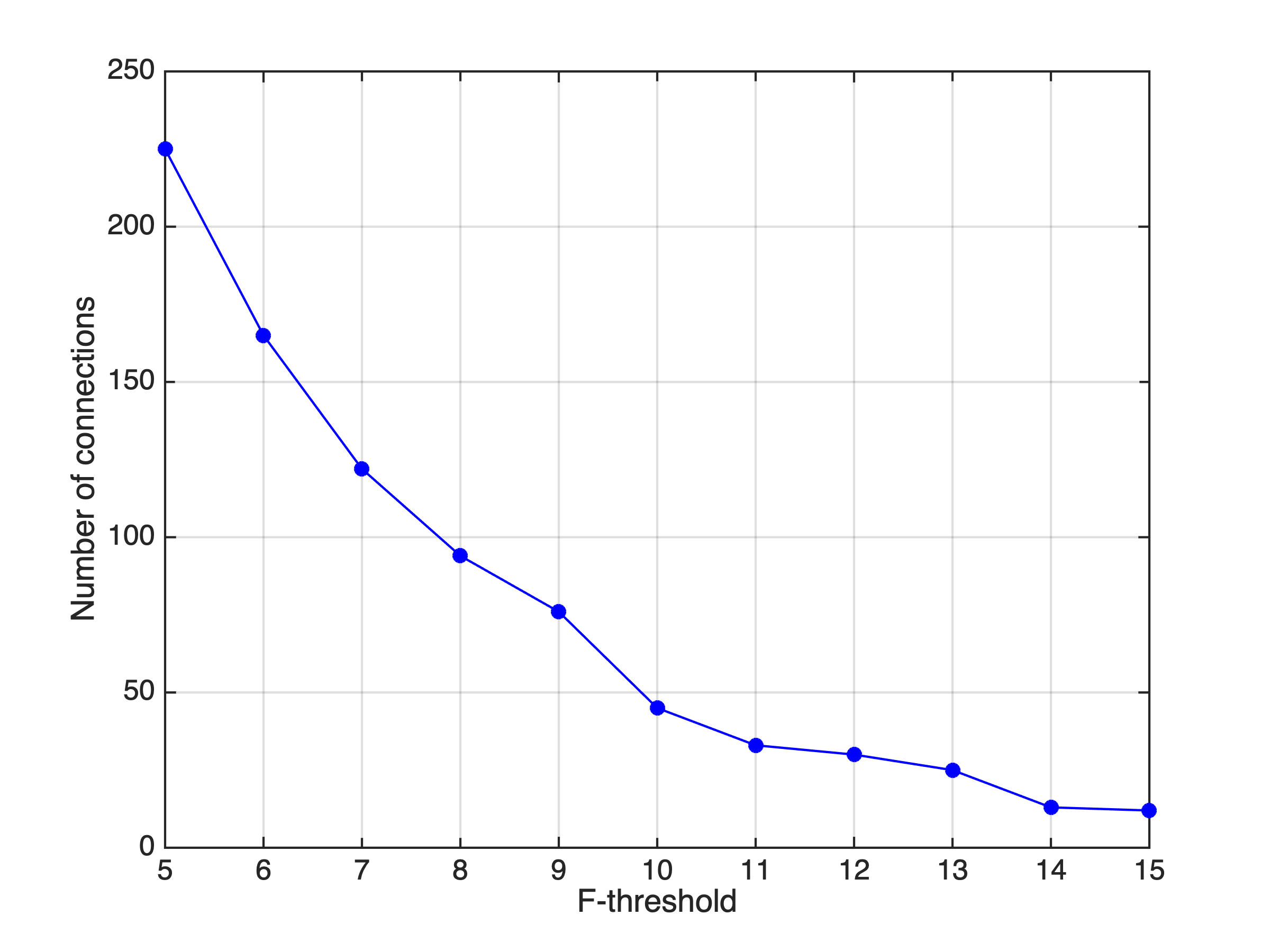

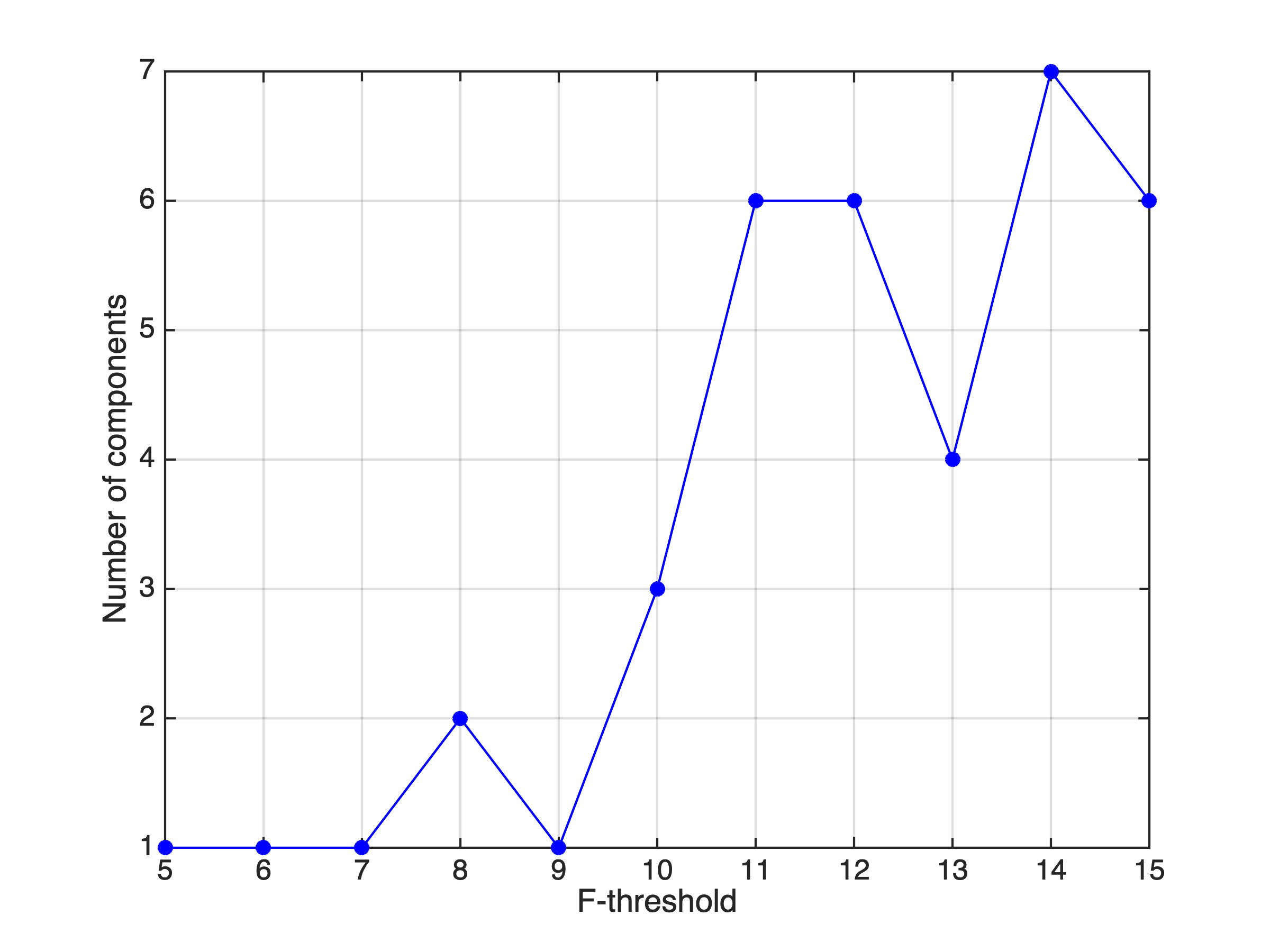


**Figure S6 *–*** Relationship between F-threshold and number of connections/components, that detected a significant component. The F-threshold used in this study (7.0) was selected based on the maximal F-threshold that detected a single component with more than two connections. This generated an NBS component with 59% nodes of the network (100 nodes) and 122 links.


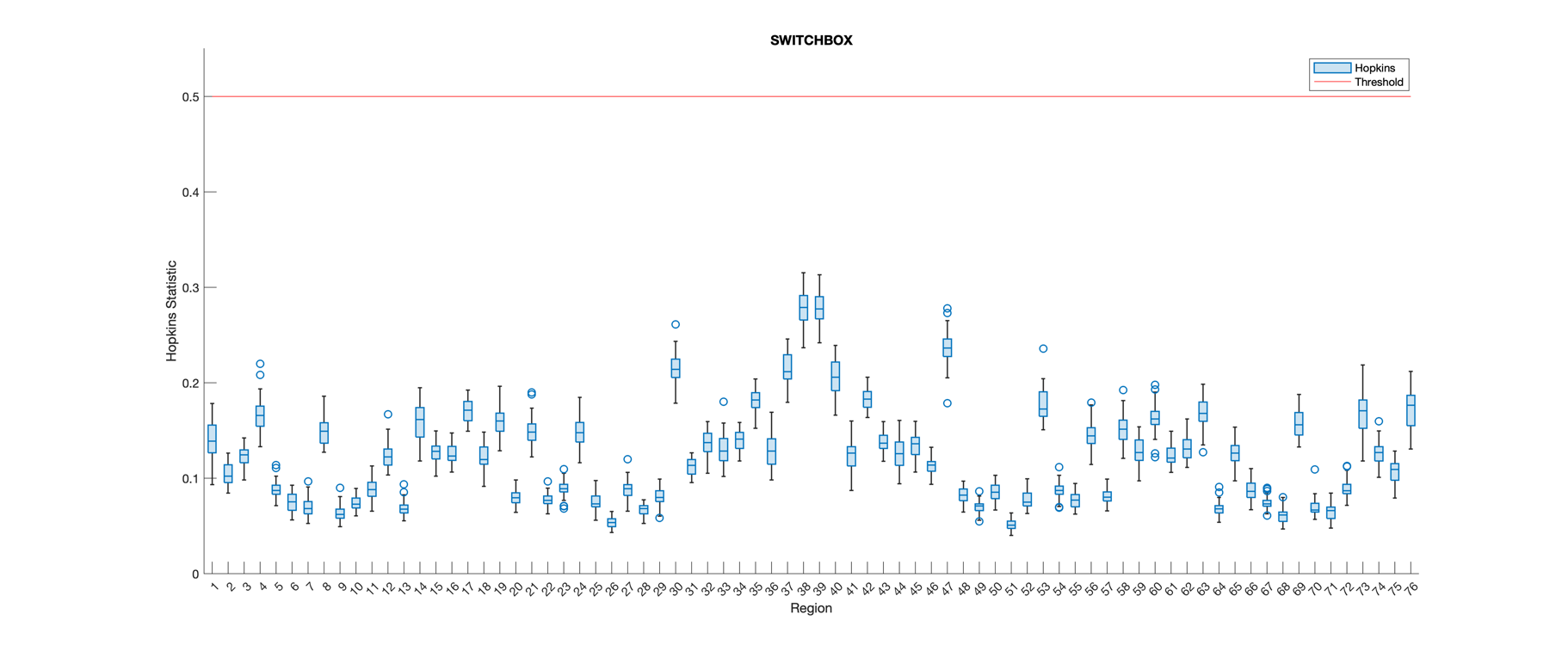

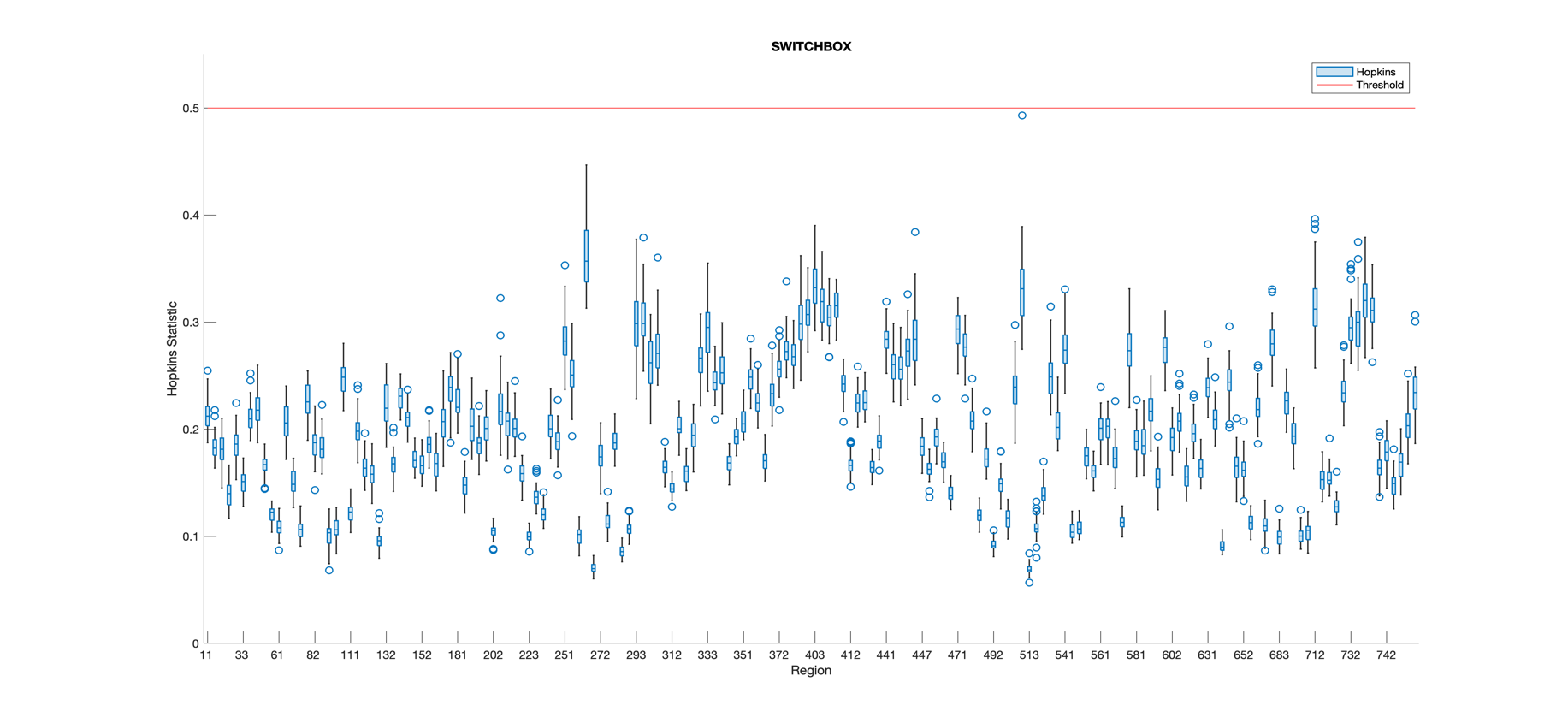


**Figure S7 –** Hopkins statistic for each region of the SWITCHBOX cohort. Top row represents regions of the DKT40 parcellation, bottom row represents regions of the generated Silhouette parcellation. Red line represents the threshold for rejecting the null hypothesis, where values lower than this threshold indicate that we can reject the null hypothesis that the data originates from a uniform distribution. All regions present values lower than 0.5, thus they present non uniform data.

.

**Figure S8 –** Hopkins statistic for each region of the MGH-HCP cohort. Top row represents regions of the DKT40 parcellation, bottom row represents regions of the generated Silhouette parcellation. Red line represents the threshold for rejecting the null hypothesis, where values lower than this threshold indicate that we can reject the null hypothesis that the data originates from a uniform distribution. All regions present values lower than 0.5, thus they present non uniform data.

.


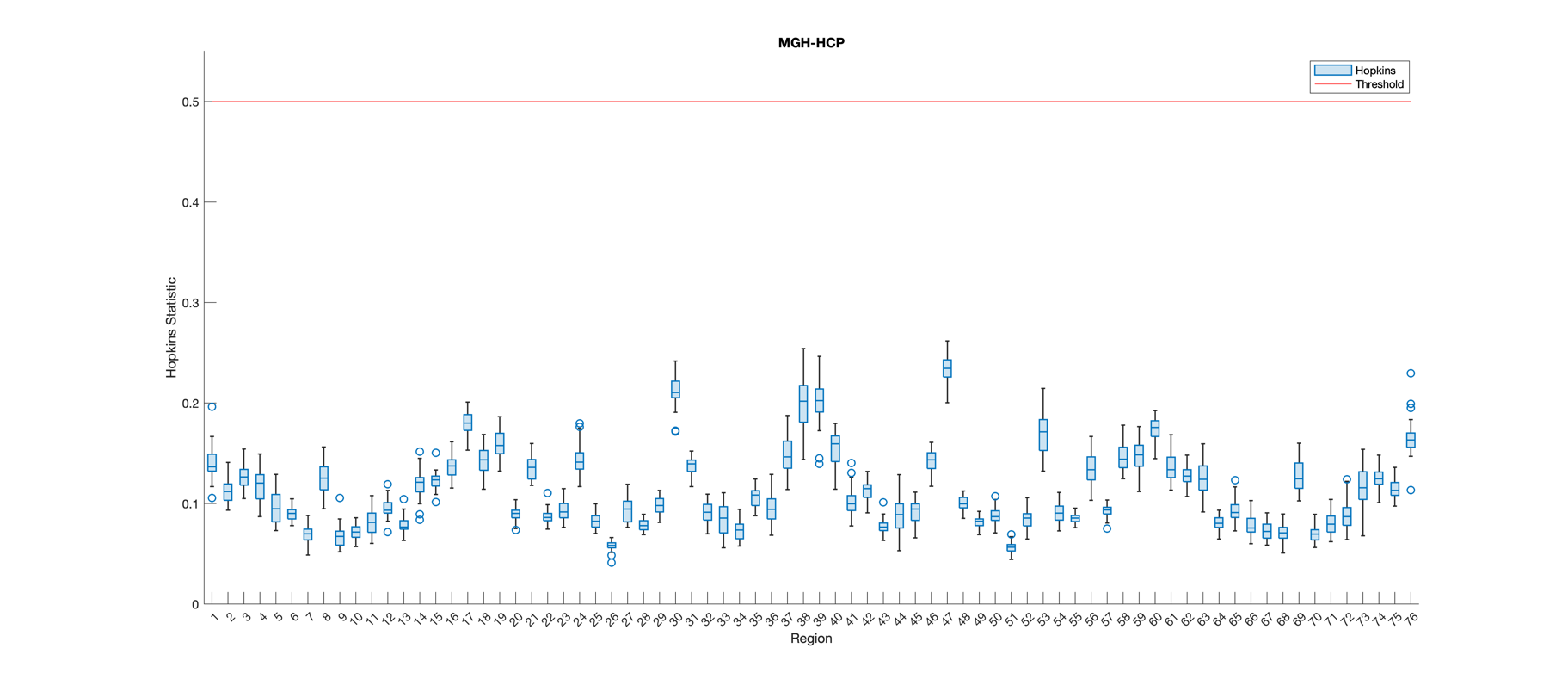

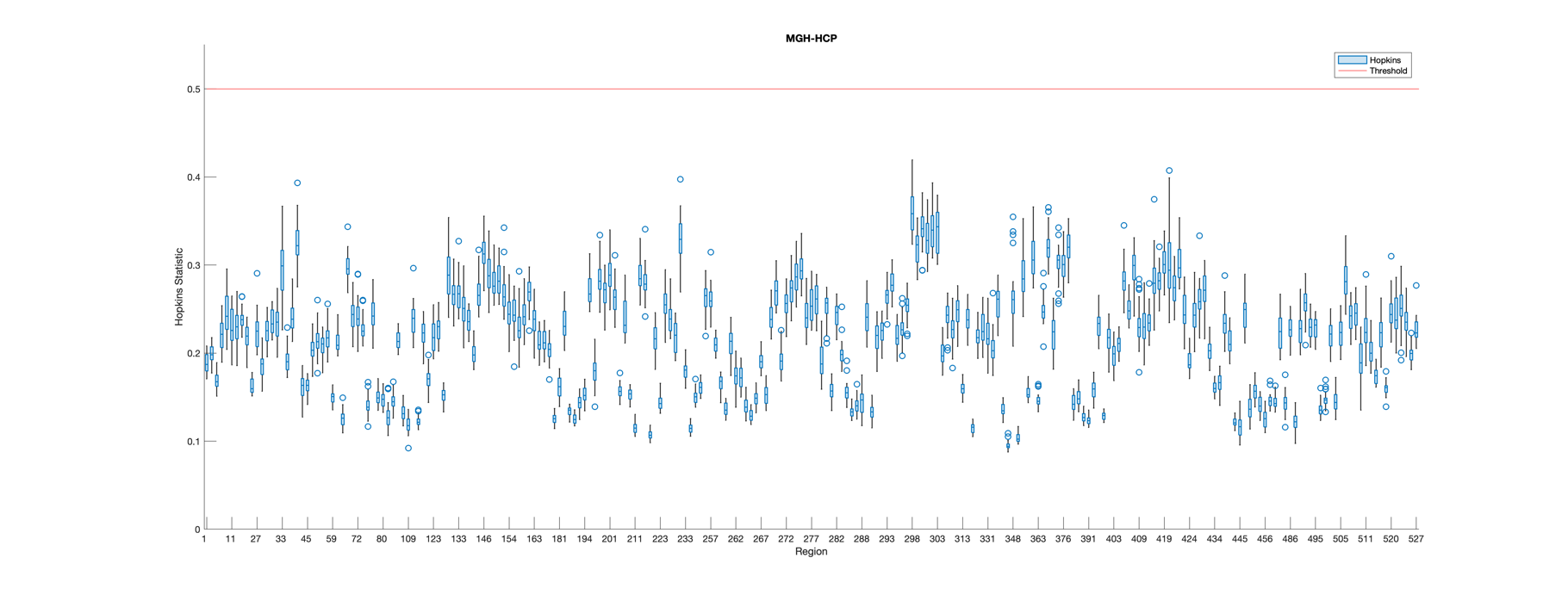


**
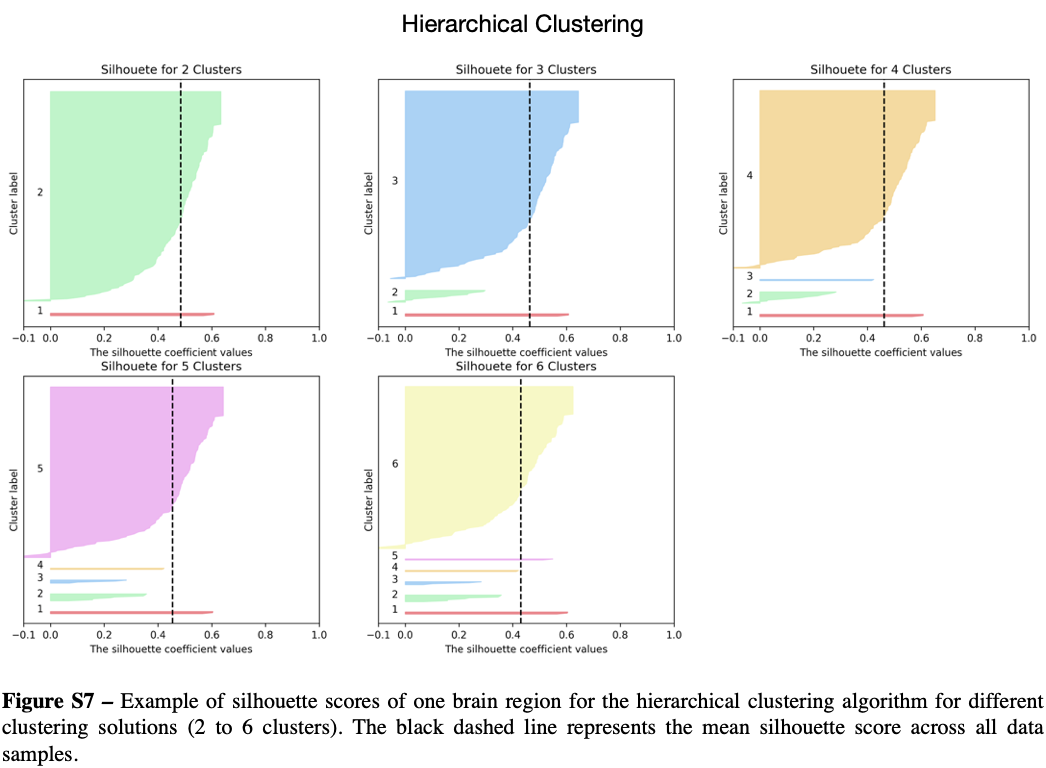
**

**
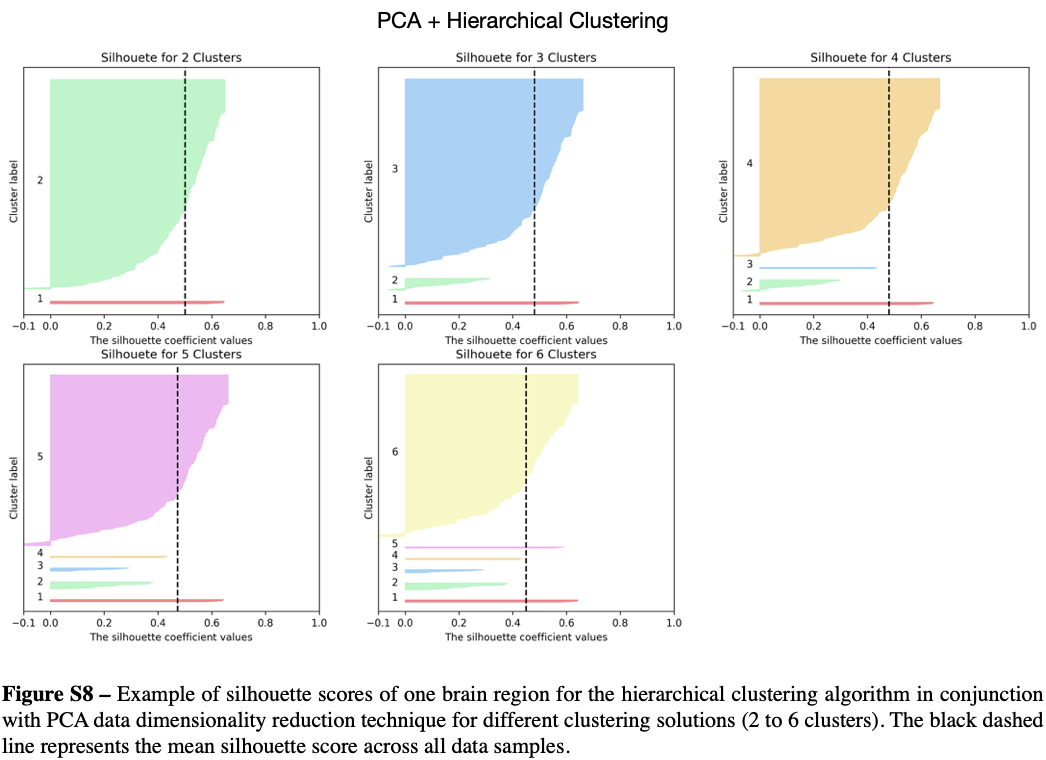
**

**Figure S9 *–*** Example of silhouette scores of one brain region for the hierarchical clustering algorithm for different clustering solutions (2 to 6 clusters). The black dashed line represents the mean silhouette score across all data samples.

**Figure S10 *–*** Example of silhouette scores of one brain region for the hierarchical clustering algorithm in conjunction with PCA data dimensionality reduction technique for different clustering solutions (2 to 6 clusters). The black dashed line represents the mean silhouette score across all data samples.

**
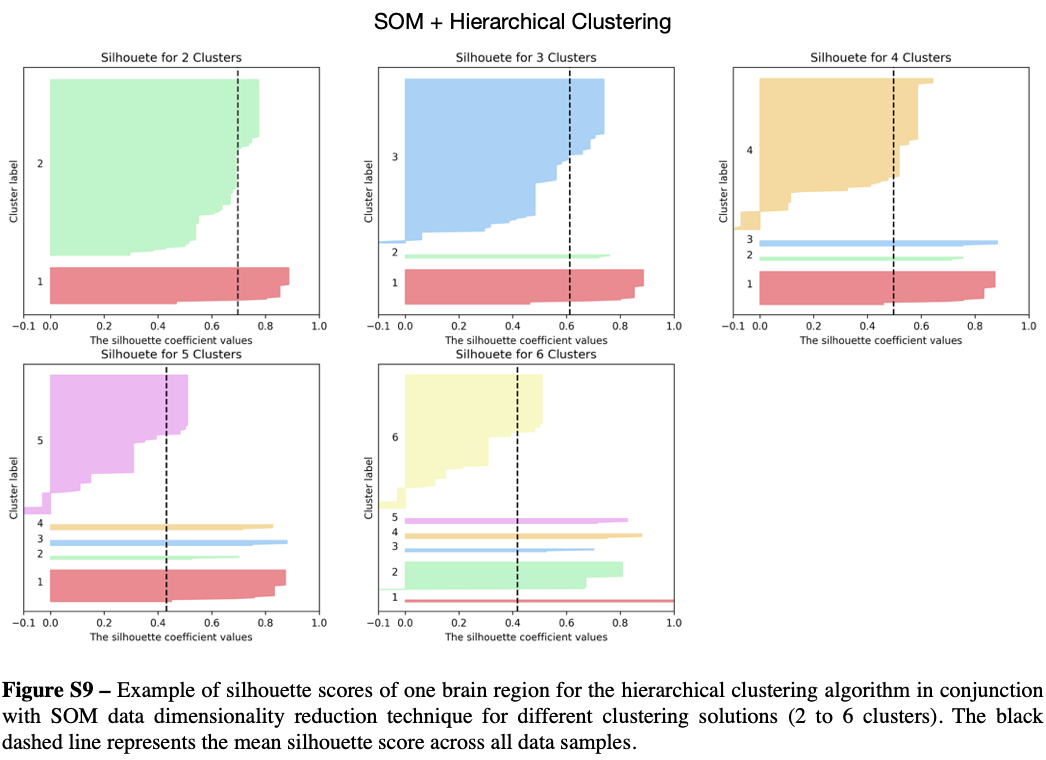
**

**
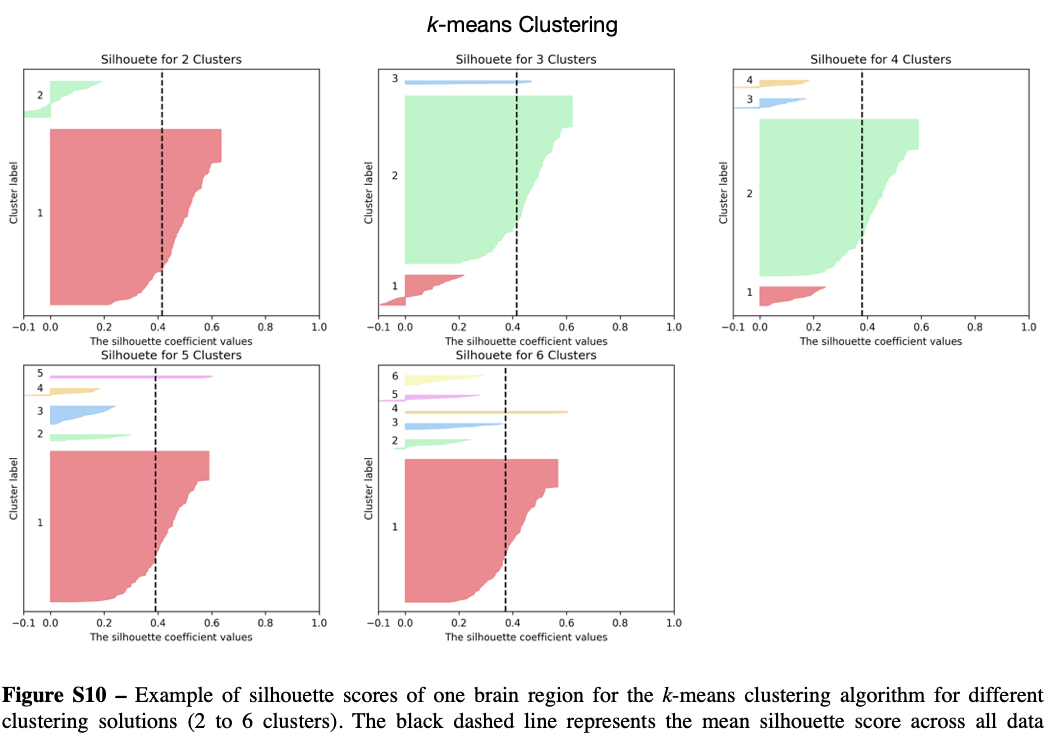
**

**Figure S12 *–*** Example of silhouette scores of one brain region for the *k*-means clustering algorithm for different clustering solutions (2 to 6 clusters). The black dashed line represents the mean silhouette score across all data samples.

**Figure S11 *–*** Example of silhouette scores of one brain region for the hierarchical clustering algorithm in conjunction with SOM data dimensionality reduction technique for different clustering solutions (2 to 6 clusters). The black dashed line represents the mean silhouette score across all data samples.

**
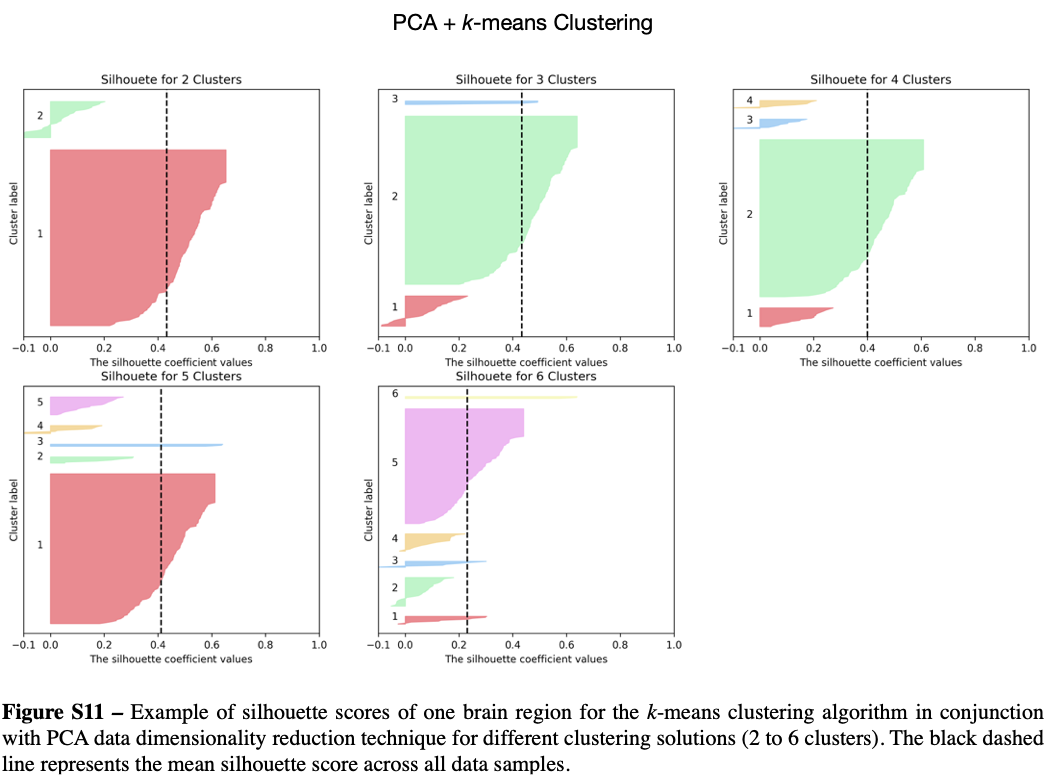
**

**Figure S13 *–*** Example of silhouette scores of one brain region for the *k*-means clustering algorithm in conjunction with PCA data dimensionality reduction technique for different clustering solutions (2 to 6 clusters). The black dashed line represents the mean silhouette score across all data samples.


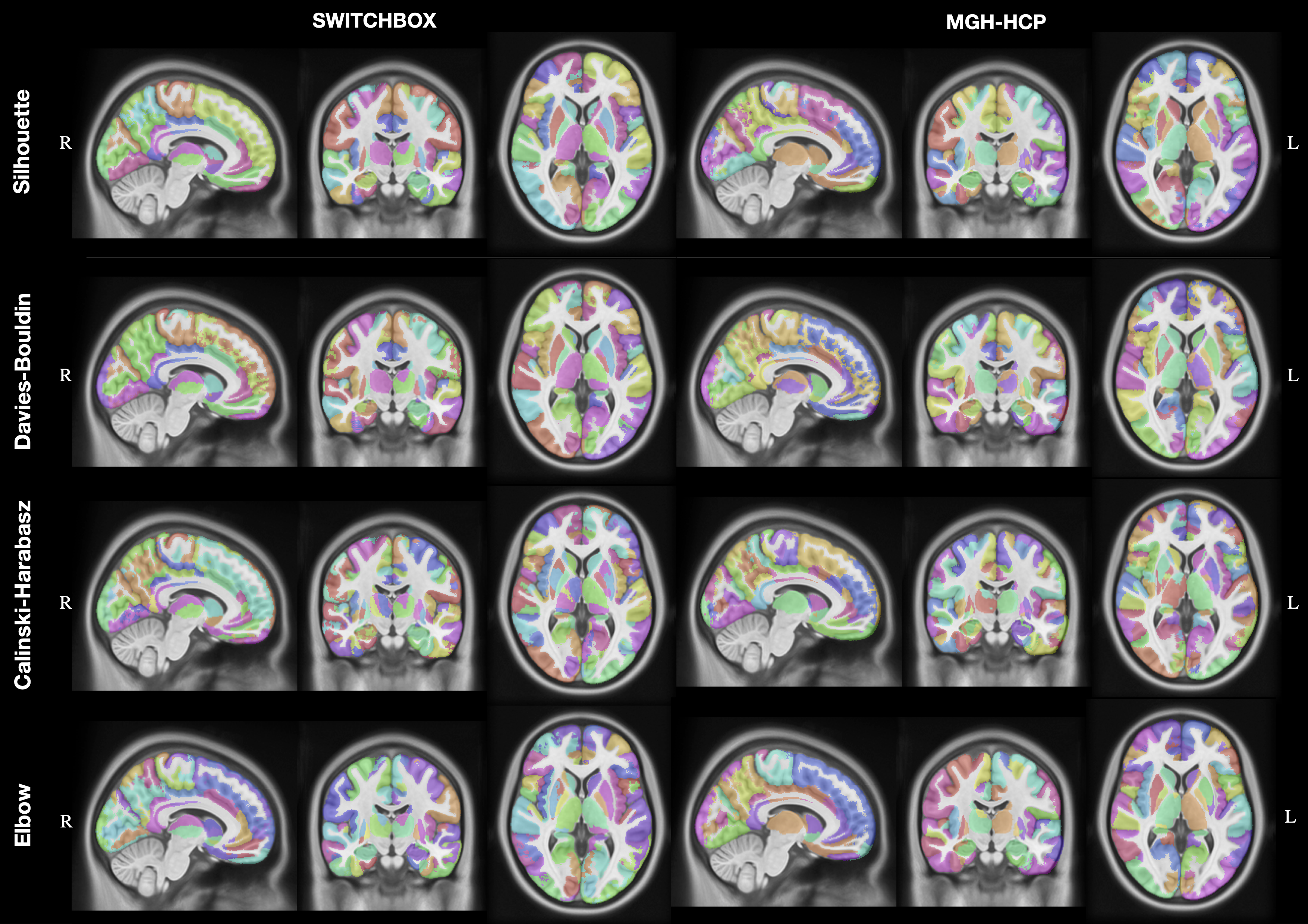

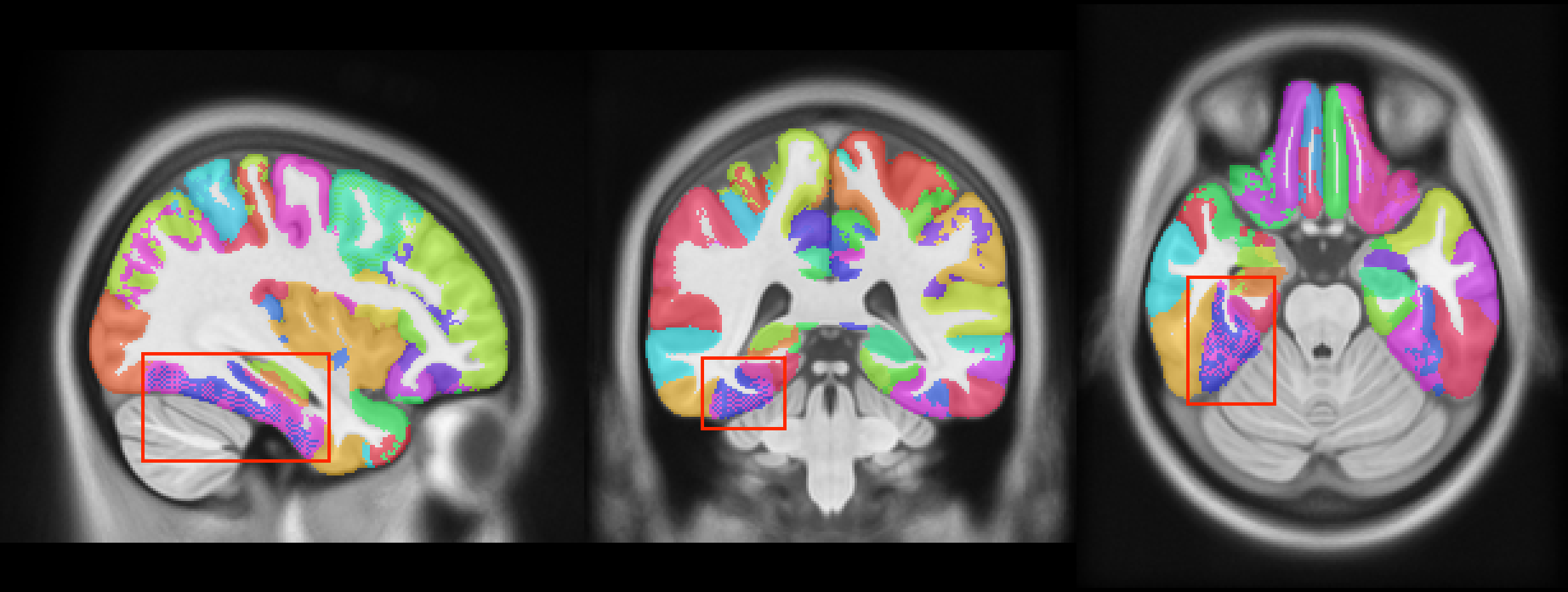


**Figure S14 –** Final group parcellations for the different validity metrics. Parcellations are displayed in MNI standard space. Parcellations of the SWITCHBOX cohort have 170 clusters (Silhouette), 163 clusters (Davies-Bouldin), 472 clusters (Calinski-Harabasz) and 165 clusters (Elbow). Regarding MGH-HCP cohort, parcellations have 241 clusters (Silhouette), 176 clusters (Davies-Bouldin), 444 clusters (Calinski-Harabasz) and 167 clusters (Elbow). The Calinski-Harabasz coefficient led to parcellations with higher number of clusters. For example, the right thalamus has the same number of clusters for the Silhouette, Davies-Bouldin and Elbow parcellations, while the Calinski-Harabasz parcellation divided this region in a higher number of clusters.

**Figure S15 –** Davies-Bouldin parcellation with the region displaying a checkerboard pattern highlighted.

**Supplementary Tables**

**Table S1 –** Skewness and kurtosis values for the distribution of structural connectivity values for a region and the different transforms applied to normalize values. Log and Box-Cox transforms are the closest to the values of a normal distribution.

| Distribution | Skewness | Kurtosis |
| --- | --- | --- |
| Original | 4.29 | 27.3 |
| Log | 0.99 | 3.38 |
| Cubic Root | 1.79 | 6.45 |
| Box-Cox | 0.18 | 1.97 |

**Table S2 –** Labels and center of gravity coordinates, in mm, of each region of the Silhouette parcellation for the SWITCHBOX dataset.

|  | Center of gravity coordinates (mm) | | |
| --- | --- | --- | --- |
| Name | x | y | z |
| Left Caudal Anterior Cingulate 1 | -4.71 | 24.02 | 24.08 |
| Left Caudal Anterior Cingulate 2 | -7.15 | 17.85 | 35.08 |
| Left Caudal Middle Frontal 1 | -43.34 | 11.66 | 38.03 |
| Left Caudal Middle Frontal 2 | -35.12 | 11.33 | 53.19 |
| Left Cuneus 1 | -13.89 | -77.10 | 29.30 |
| Left Cuneus 2 | -3.89 | -84.27 | 18.55 |
| Left Entorhinal 1 | -21.06 | -5.00 | -31.00 |
| Left Entorhinal 2 | -28.92 | -8.75 | -34.92 |
| Left Fusiform 1 | -35.56 | -33.82 | -20.03 |
| Left Fusiform 2 | -36.54 | -55.93 | -17.80 |
| Left Inferior Parietal 1 | -43.75 | -69.80 | 32.95 |
| Left Inferior Parietal 2 | -30.01 | -75.87 | 35.05 |
| Left Inferior Temporal 1 | -50.30 | -49.66 | -13.79 |
| Left Inferior Temporal 2 | -49.65 | -23.88 | -31.85 |
| Left Isthmus Cingulate 1 | -12.25 | -48.91 | 5.02 |
| Left Isthmus Cingulate 2 | -5.90 | -45.06 | 24.94 |
| Left Lateral Occipital 1 | -45.67 | -75.76 | 2.91 |
| Left Lateral Occipital 2 | -26.39 | -93.82 | 0.50 |
| Left Lateral Orbitofrontal 1 | -23.78 | 38.27 | -19.12 |
| Left Lateral Orbitofrontal 2 | -22.46 | 5.24 | -15.80 |
| Left Lingual 1 | -12.28 | -72.85 | -6.28 |
| Left Lingual 2 | -18.95 | -57.79 | 1.43 |
| Left Medial Orbitofrontal 1 | -6.38 | 21.79 | -19.62 |
| Left Medial Orbitofrontal 2 | -5.37 | 49.93 | -16.74 |
| Left Middle Temporal 1 | -59.78 | -32.12 | -10.50 |
| Left Middle Temporal 2 | -45.74 | 8.94 | -38.73 |
| Left Middle Temporal 3 | -55.78 | -36.57 | -0.60 |
| Left Parahippocampal 1 | -30.02 | -29.00 | -21.48 |
| Left Parahippocampal 2 | -20.53 | -33.71 | -16.25 |
| Left Paracentral 1 | -9.79 | -31.39 | 54.01 |
| Left Paracentral 2 | -4.83 | -21.84 | 62.74 |
| Left Pars Opercularis 1 | -45.25 | 14.01 | 5.88 |
| Left Pars Opercularis 2 | -51.21 | 14.56 | 18.66 |
| Left Pars Orbitalis 1 | -46.34 | 35.19 | -15.26 |
| Left Pars Orbitalis 2 | -39.82 | 26.53 | -6.81 |
| Left Pars Triangularis 1 | -41.86 | 26.29 | 1.99 |
| Left Pars Triangularis 2 | -50.85 | 33.33 | 5.53 |
| Left Pericalcarine 1 | -14.98 | -73.68 | 9.55 |
| Left Pericalcarine 2 | -8.86 | -89.42 | 3.42 |
| Left Postcentral 1 | -22.17 | -33.83 | 69.91 |
| Left Postcentral 2 | -50.83 | -21.20 | 39.50 |
| Left Postcentral 3 | -15.17 | -45.55 | 76.45 |
| Left Posterior Cingulate 1 | -8.78 | -16.54 | 41.07 |
| Left Posterior Cingulate 2 | -3.97 | -21.90 | 36.04 |
| Left Precentral 1 | -22.32 | -21.57 | 70.60 |
| Left Precentral 2 | -47.76 | -4.59 | 35.59 |
| Left Precuneus 1 | -6.99 | -59.41 | 52.34 |
| Left Precuneus 2 | -9.53 | -58.97 | 30.30 |
| Left Rostral Anterior Cingulate 1 | -4.44 | 29.35 | -5.06 |
| Left Rostral Anterior Cingulate 2 | -6.57 | 43.38 | 4.70 |
| Left Rostral Middle Frontal 1 | -28.73 | 48.26 | 19.11 |
| Left Rostral Middle Frontal 2 | -26.78 | 33.93 | 38.53 |
| Left Rostral Middle Frontal 3 | -39.54 | 43.20 | 18.05 |
| Left Superior Frontal 1 | -2.52 | -1.40 | 64.85 |
| Left Superior Frontal 2 | -13.90 | 35.16 | 39.18 |
| Left Superior Parietal 1 | -23.97 | -54.43 | 65.35 |
| Left Superior Parietal 2 | -25.35 | -65.63 | 49.27 |
| Left Superior Temporal 1 | -32.87 | 12.42 | -30.24 |
| Left Superior Temporal 2 | -53.28 | -14.33 | -4.89 |
| Left Supramarginal 1 | -55.20 | -37.98 | 34.68 |
| Left Supramarginal 2 | -58.07 | -56.02 | 26.62 |
| Left Supramarginal 3 | -48.28 | -45.05 | 44.20 |
| Left Transverse Temporal 1 | -44.57 | -25.11 | 11.38 |
| Left Transverse Temporal 2 | -46.05 | -18.51 | 5.84 |
| Left Insula 1 | -37.64 | -9.66 | 0.77 |
| Left Insula 2 | -36.04 | 8.76 | -0.48 |
| Left Thalamus Proper 1 | -13.44 | -20.87 | 9.62 |
| Left Thalamus Proper 2 | -13.73 | -16.81 | 3.98 |
| Left Caudate 1 | -13.44 | 8.23 | 13.07 |
| Left Caudate 2 | -16.90 | -2.40 | 16.91 |
| Left Caudate 3 | -18.92 | -3.33 | 22.81 |
| Left Caudate 4 | -12.17 | 15.73 | -1.87 |
| Left Caudate 5 | -17.51 | 17.78 | 5.52 |
| Left Putamen 1 | -25.93 | -0.28 | 5.94 |
| Left Putamen 2 | -26.05 | 2.38 | -5.17 |
| Left Pallidum 1 | -22.48 | -9.66 | 1.42 |
| Left Pallidum 2 | -18.49 | -0.95 | -1.86 |
| Left Hippocampus 1 | -30.15 | -25.52 | -10.39 |
| Left Hippocampus 2 | -24.93 | -21.59 | -16.11 |
| Left Amygdala 1 | -21.99 | -4.71 | -20.55 |
| Left Amygdala 2 | -27.77 | -6.79 | -18.27 |
| Left Accumbens Area 1 | -10.34 | 11.45 | -7.95 |
| Right Accumbens Area 1 | 9.69 | 12.30 | -7.62 |
| Right Amygdala 1 | 25.54 | -3.52 | -27.22 |
| Right Amygdala 2 | 19.11 | -5.82 | -19.27 |
| Right Amygdala 3 | 21.76 | -1.69 | -19.35 |
| Right Amygdala 4 | 27.45 | -7.56 | -17.68 |
| Right Amygdala 5 | 24.89 | -2.81 | -16.60 |
| Right Amygdala 6 | 29.06 | -4.29 | -16.56 |
| Right Hippocampus 1 | 29.20 | -25.17 | -8.30 |
| Right Hippocampus 2 | 26.14 | -20.98 | -15.77 |
| Right Pallidum 1 | 23.67 | -10.37 | 1.34 |
| Right Pallidum 2 | 18.46 | -0.19 | -0.54 |
| Right Putamen 1 | 23.14 | 6.79 | -0.01 |
| Right Putamen 2 | 29.76 | -3.96 | 0.23 |
| Right Caudate 1 | 15.33 | 3.20 | 13.88 |
| Right Caudate 2 | 17.91 | 19.41 | 5.20 |
| Right Caudate 3 | 12.06 | 18.11 | -3.13 |
| Right Caudate 4 | 18.89 | -7.89 | 22.23 |
| Right Caudate 5 | 10.19 | 7.25 | 10.82 |
| Right Caudate 6 | 14.31 | 10.41 | 12.74 |
| Right Thalamus Proper 1 | 13.10 | -15.59 | 4.04 |
| Right Thalamus Proper 2 | 13.42 | -19.34 | 9.69 |
| Right Insula 1 | 38.28 | -4.91 | -3.65 |
| Right Insula 2 | 35.79 | 6.61 | 1.84 |
| Right Transverse Temporal 1 | 46.63 | -14.94 | 4.90 |
| Right Transverse Temporal 2 | 44.03 | -21.14 | 10.83 |
| Right Supramarginal 1 | 44.70 | -25.22 | 19.71 |
| Right Supramarginal 2 | 55.79 | -33.34 | 37.81 |
| Right Superior Temporal 1 | 30.02 | 14.75 | -33.21 |
| Right Superior Temporal 2 | 54.48 | -10.98 | -4.18 |
| Right Superior Parietal 1 | 21.41 | -56.28 | 65.80 |
| Right Superior Parietal 2 | 27.12 | -65.00 | 47.14 |
| Right Superior Frontal 1 | 5.94 | 47.29 | -4.53 |
| Right Superior Frontal 2 | 4.79 | -2.94 | 62.96 |
| Right Superior Frontal 3 | 13.61 | 35.49 | 37.77 |
| Right Rostral Middle Frontal 1 | 38.83 | 47.47 | 15.39 |
| Right Rostral Middle Frontal 2 | 38.29 | 32.15 | 29.71 |
| Right Rostral Anterior Cingulate 1 | 6.81 | 42.18 | 8.86 |
| Right Rostral Anterior Cingulate 2 | 4.22 | 32.89 | -2.64 |
| Right Precuneus 1 | 10.29 | -48.01 | 69.24 |
| Right Precuneus 2 | 9.62 | -58.33 | 38.25 |
| Right Precentral 1 | 46.31 | -2.15 | 35.51 |
| Right Precentral 2 | 59.33 | 6.07 | 28.27 |
| Right Precentral 3 | 25.38 | -21.00 | 67.83 |
| Right Posterior Cingulate 1 | 8.22 | -15.20 | 41.73 |
| Right Posterior Cingulate 2 | 4.21 | -20.98 | 36.46 |
| Right Postcentral 1 | 21.20 | -34.63 | 71.40 |
| Right Postcentral 2 | 50.99 | -17.43 | 37.36 |
| Right Postcentral 3 | 11.51 | -42.59 | 78.25 |
| Right Pericalcarine 1 | 10.66 | -90.37 | 3.78 |
| Right Pericalcarine 2 | 14.03 | -73.38 | 10.89 |
| Right Pars Triangularis 1 | 43.23 | 27.16 | 2.05 |
| Right Pars Triangularis 2 | 50.52 | 35.78 | 4.29 |
| Right Pars Orbitalis 1 | 39.01 | 27.21 | -7.13 |
| Right Pars Orbitalis 2 | 44.63 | 37.44 | -15.29 |
| Right Pars Opercularis 1 | 43.92 | 17.05 | 5.80 |
| Right Pars Opercularis 2 | 51.20 | 16.74 | 16.60 |
| Right Paracentral 1 | 12.13 | -31.00 | 51.20 |
| Right Paracentral 2 | 4.51 | -22.99 | 62.16 |
| Right Parahippocampal 1 | 27.29 | -33.47 | -16.07 |
| Right Parahippocampal 2 | 21.86 | -28.17 | -20.08 |
| Right Middle Temporal 1 | 57.97 | -26.91 | -11.75 |
| Right Middle Temporal 2 | 52.09 | -36.30 | 1.78 |
| Right Medial Orbitofrontal 1 | 3.72 | 50.54 | -18.15 |
| Right Medial Orbitofrontal 2 | 5.17 | 23.78 | -19.08 |
| Right Lingual 1 | 13.45 | -68.28 | -4.16 |
| Right Lateral Orbitofrontal 1 | 18.76 | 9.89 | -15.99 |
| Right Lateral Orbitofrontal 2 | 22.70 | 40.24 | -19.25 |
| Right Lateral Occipital 1 | 43.59 | -65.72 | 2.06 |
| Right Lateral Occipital 2 | 31.63 | -87.73 | 1.19 |
| Right Isthmus Cingulate 1 | 5.31 | -43.25 | 28.77 |
| Right Isthmus Cingulate 2 | 10.86 | -45.77 | 8.46 |
| Right Inferior Temporal 1 | 48.96 | -28.54 | -27.70 |
| Right Inferior Parietal 1 | 47.64 | -63.84 | 30.98 |
| Right Inferior Parietal 2 | 44.71 | -66.96 | 14.29 |
| Right Inferior Parietal 3 | 38.93 | -61.11 | 41.50 |
| Right Fusiform 1 | 30.67 | -43.26 | -18.63 |
| Right Fusiform 2 | 35.97 | -46.58 | -20.74 |
| Right Entorhinal 1 | 20.27 | -8.02 | -32.15 |
| Right Entorhinal 2 | 21.42 | 3.41 | -30.89 |
| Right Entorhinal 3 | 25.09 | -0.01 | -38.45 |
| Right Entorhinal 4 | 28.66 | -5.82 | -35.23 |
| Right Entorhinal 5 | 29.48 | -12.87 | -33.10 |
| Right Cuneus 1 | 7.28 | -87.98 | 24.51 |
| Right Cuneus 2 | 11.38 | -72.00 | 24.17 |
| Right Caudal Middle Frontal 1 | 38.45 | 16.57 | 44.37 |
| Right Caudal Middle Frontal 2 | 34.67 | 7.26 | 53.99 |
| Right Caudal Anterior Cingulate 1 | 5.66 | 19.88 | 30.93 |
| Right Caudal Anterior Cingulate 2 | 4.93 | 22.06 | 23.24 |

**Table S3 –** Brain regions belonging to the different modules of each timepoint’s modularity community structure.

| Timepoint 1 | |  | Timepoint 2 | |
| --- | --- | --- | --- | --- |
| Module | Area |  | Module | Area |
| 1 | Left Entorhinal 1 |  | 1 | Left Cuneus 1 |
|  | Left Entorhinal 2 |  |  | Left Cuneus 2 |
|  | Left Fusiform 1 |  |  | Left Entorhinal 1 |
|  | Left Fusiform 2 |  |  | Left Entorhinal 2 |
|  | Left Inferior Temporal 1 |  |  | Left Fusiform 1 |
|  | Left Inferior Temporal 2 |  |  | Left Fusiform 2 |
|  | Left Middle Temporal 1 |  |  | Left Inferior Temporal 1 |
|  | Left Middle Temporal 2 |  |  | Left Inferior Temporal 2 |
|  | Left Middle Temporal 3 |  |  | Left Lateral Occipital 1 |
|  | Left Parahippocampal 1 |  |  | Left Lateral Occipital 2 |
|  | Left Parahippocampal 2 |  |  | Left Lingual 1 |
|  | Left Superior Temporal 1 |  |  | Left Lingual 2 |
|  | Left Superior Temporal 2 |  |  | Left Middle Temporal 1 |
|  | Left Transverse Temporal 1 |  |  | Left Middle Temporal 2 |
|  | Left Transverse Temporal 2 |  |  | Left Middle Temporal 3 |
|  | Left Hippocampus 1 |  |  | Left Parahippocampal 1 |
|  | Left Hippocampus 2 |  |  | Left Parahippocampal 2 |
|  | Left Amygdala 1 |  |  | Left Pericalcarine 1 |
|  | Left Amygdala 2 |  |  | Left Pericalcarine 2 |
| 2 | Left Caudal Anterior Cingulate 1 |  |  | Left Superior Temporal 1 |
|  | Left Caudal Anterior Cingulate 2 |  |  | Left Superior Temporal 2 |
|  | Left Caudal Middle Frontal 1 |  |  | Left Transverse Temporal 1 |
|  | Left Caudal Middle Frontal 2 |  |  | Left Transverse Temporal 2 |
|  | Left Lateral Orbitofrontal 1 |  |  | Left Hippocampus 1 |
|  | Left Lateral Orbitofrontal 2 |  |  | Left Hippocampus 2 |
|  | Left Medial Orbitofrontal 1 |  |  | Left Amygdala 1 |
|  | Left Medial Orbitofrontal 2 |  |  | Left Amygdala 2 |
|  | Left Pars Opercularis 1 |  | 2 | Left Caudal Middle Frontal 1 |
|  | Left Pars Opercularis 2 |  |  | Left Caudal Middle Frontal 2 |
|  | Left Pars Orbitalis 1 |  |  | Left Lateral Orbitofrontal 1 |
|  | Left Pars Orbitalis 2 |  |  | Left Lateral Orbitofrontal 2 |
|  | Left Pars Triangularis 1 |  |  | Left Medial Orbitofrontal 1 |
|  | Left Pars Triangularis 2 |  |  | Left Medial Orbitofrontal 2 |
|  | Left Postcentral 1 |  |  | Left Pars Opercularis 1 |
|  | Left Postcentral 2 |  |  | Left Pars Opercularis 2 |
|  | Left Precentral 1 |  |  | Left Pars Orbitalis 1 |
|  | Left Precentral 2 |  |  | Left Pars Orbitalis 2 |
|  | Left Rostral Anterior Cingulate 1 |  |  | Left Pars Triangularis 1 |
|  | Left Rostral Anterior Cingulate 2 |  |  | Left Pars Triangularis 2 |
|  | Left Rostral Middle Frontal 1 |  |  | Left Rostral Anterior Cingulate 1 |
|  | Left Rostral Middle Frontal 2 |  |  | Left Rostral Anterior Cingulate 2 |
|  | Left Rostral Middle Frontal 3 |  |  | Left Rostral Middle Frontal 1 |
|  | Left Superior Frontal 1 |  |  | Left Rostral Middle Frontal 2 |
|  | Left Superior Frontal 2 |  |  | Left Rostral Middle Frontal 3 |
|  | Left Insula 1 |  |  | Left Superior Frontal 1 |
|  | Left Insula 2 |  |  | Left Superior Frontal 2 |
|  | Left Putamen 1 |  |  | Left Insula 1 |
|  | Left Putamen 2 |  |  | Left Insula 2 |
|  | Left Pallidum 1 |  |  | Left Thalamus Proper 1 |
|  | Left Pallidum 2 |  |  | Left Thalamus Proper 2 |
|  | Left Accumbens Area 1 |  |  | Left Caudate 1 |
| 3 | Left Inferior Parietal 1 |  |  | Left Caudate 2 |
|  | Left Inferior Parietal 2 |  |  | Left Caudate 3 |
|  | Left Postcentral 3 |  |  | Left Caudate 4 |
|  | Left Superior Parietal 1 |  |  | Left Caudate 5 |
|  | Left Superior Parietal 2 |  |  | Left Putamen 1 |
|  | Left Supramarginal 1 |  |  | Left Putamen 2 |
|  | Left Supramarginal 2 |  |  | Left Pallidum 1 |
|  | Left Supramarginal 3 |  |  | Left Pallidum 2 |
| 4 | Left Thalamus Proper 1 |  |  | Left Accumbens Area 1 |
|  | Left Thalamus Proper 2 |  | 3 | Left Inferior Parietal 1 |
|  | Left Caudate 1 |  |  | Left Inferior Parietal 2 |
|  | Left Caudate 2 |  |  | Left Postcentral 1 |
|  | Left Caudate 3 |  |  | Left Postcentral 2 |
|  | Left Caudate 4 |  |  | Left Postcentral 3 |
|  | Left Caudate 5 |  |  | Left Precentral 1 |
| 5 | Right Amygdala 1 |  |  | Left Precentral 2 |
|  | Right Amygdala 2 |  |  | Left Superior Parietal 1 |
|  | Right Amygdala 3 |  |  | Left Superior Parietal 2 |
|  | Right Amygdala 4 |  |  | Left Supramarginal 1 |
|  | Right Amygdala 5 |  |  | Left Supramarginal 2 |
|  | Right Amygdala 6 |  |  | Left Supramarginal 3 |
|  | Right Hippocampus 1 |  | 4 | Right Caudate 1 |
|  | Right Hippocampus 2 |  |  | Right Caudate 2 |
|  | Right Superior Temporal 1 |  |  | Right Caudate 4 |
|  | Right Parahippocampal 1 |  |  | Right Caudate 5 |
|  | Right Parahippocampal 2 |  |  | Right Caudate 6 |
|  | Right Inferior Temporal 1 |  |  | Right Thalamus Proper 1 |
|  | Right Fusiform 1 |  |  | Right Thalamus Proper 2 |
|  | Right Fusiform 2 |  | 5 | Right Accumbens Area 1 |
|  | Right Entorhinal 1 |  |  | Right Pallidum 1 |
|  | Right Entorhinal 2 |  |  | Right Pallidum 2 |
|  | Right Entorhinal 3 |  |  | Right Putamen 1 |
|  | Right Entorhinal 4 |  |  | Right Putamen 2 |
|  | Right Entorhinal 5 |  |  | Right Caudate 3 |
| 6 | Left Cuneus 1 |  |  | Right Insula 1 |
|  | Left Cuneus 2 |  |  | Right Insula 2 |
|  | Left Isthmus Cingulate 1 |  |  | Right Superior Frontal 1 |
|  | Left Isthmus Cingulate 2 |  |  | Right Superior Frontal 2 |
|  | Left Lateral Occipital 1 |  |  | Right Superior Frontal 3 |
|  | Left Lateral Occipital 2 |  |  | Right Rostral Middle Frontal 1 |
|  | Left Lingual 1 |  |  | Right Rostral Middle Frontal 2 |
|  | Left Lingual 2 |  |  | Right Rostral Anterior Cingulate 1 |
|  | Left Paracentral 1 |  |  | Right Rostral Anterior Cingulate 2 |
|  | Left Paracentral 2 |  |  | Right Precentral 1 |
|  | Left Pericalcarine 1 |  |  | Right Precentral 2 |
|  | Left Pericalcarine 2 |  |  | Right Precentral 3 |
|  | Left Posterior Cingulate 1 |  |  | Right Pars Triangularis 1 |
|  | Left Posterior Cingulate 2 |  |  | Right Pars Triangularis 2 |
|  | Left Precuneus 1 |  |  | Right Pars Orbitalis 1 |
|  | Left Precuneus 2 |  |  | Right Pars Orbitalis 2 |
|  | Right Precuneus 1 |  |  | Right Pars Opercularis 1 |
|  | Right Precuneus 2 |  |  | Right Pars Opercularis 2 |
|  | Right Posterior Cingulate 1 |  |  | Right Medial Orbitofrontal 1 |
|  | Right Posterior Cingulate 2 |  |  | Right Medial Orbitofrontal 2 |
|  | Right Pericalcarine 1 |  |  | Right Lateral Orbitofrontal 1 |
|  | Right Pericalcarine 2 |  |  | Right Lateral Orbitofrontal 2 |
|  | Right Paracentral 1 |  |  | Right Caudal Middle Frontal 1 |
|  | Right Paracentral 2 |  |  | Right Caudal Middle Frontal 2 |
|  | Right Lingual 1 |  | 6 | Left Caudal Anterior Cingulate 1 |
|  | Right Lateral Occipital 1 |  |  | Left Caudal Anterior Cingulate 2 |
|  | Right Lateral Occipital 2 |  |  | Left Isthmus Cingulate 1 |
|  | Right Isthmus Cingulate 1 |  |  | Left Isthmus Cingulate 2 |
|  | Right Isthmus Cingulate 2 |  |  | Left Paracentral 1 |
|  | Right Cuneus 1 |  |  | Left Paracentral 2 |
|  | Right Cuneus 2 |  |  | Left Posterior Cingulate 1 |
| 7 | Right Insula 1 |  |  | Left Posterior Cingulate 2 |
|  | Right Insula 2 |  |  | Left Precuneus 1 |
|  | Right Transverse Temporal 1 |  |  | Left Precuneus 2 |
|  | Right Transverse Temporal 2 |  |  | Right Precuneus 1 |
|  | Right Supramarginal 1 |  |  | Right Precuneus 2 |
|  | Right Supramarginal 2 |  |  | Right Posterior Cingulate 1 |
|  | Right Superior Temporal 2 |  |  | Right Posterior Cingulate 2 |
|  | Right Superior Parietal 1 |  |  | Right Postcentral 1 |
|  | Right Superior Parietal 2 |  |  | Right Postcentral 3 |
|  | Right Rostral Middle Frontal 1 |  |  | Right Paracentral 1 |
|  | Right Rostral Middle Frontal 2 |  |  | Right Paracentral 2 |
|  | Right Precentral 1 |  |  | Right Isthmus Cingulate 1 |
|  | Right Precentral 2 |  |  | Right Isthmus Cingulate 2 |
|  | Right Precentral 3 |  |  | Right Caudal Anterior Cingulate 1 |
|  | Right Postcentral 1 |  |  | Right Caudal Anterior Cingulate 2 |
|  | Right Postcentral 2 |  | 7 | Right Transverse Temporal 1 |
|  | Right Postcentral 3 |  |  | Right Transverse Temporal 2 |
|  | Right Pars Triangularis 1 |  |  | Right Supramarginal 1 |
|  | Right Pars Triangularis 2 |  |  | Right Supramarginal 2 |
|  | Right Pars Orbitalis 1 |  |  | Right Superior Temporal 2 |
|  | Right Pars Orbitalis 2 |  |  | Right Superior Parietal 1 |
|  | Right Pars Opercularis 1 |  |  | Right Superior Parietal 2 |
|  | Right Pars Opercularis 2 |  |  | Right Postcentral 2 |
|  | Right Middle Temporal 1 |  |  | Right Pericalcarine 1 |
|  | Right Middle Temporal 2 |  |  | Right Pericalcarine 2 |
|  | Right Inferior Parietal 1 |  |  | Right Middle Temporal 1 |
|  | Right Inferior Parietal 2 |  |  | Right Middle Temporal 2 |
|  | Right Inferior Parietal 3 |  |  | Right Lingual 1 |
|  | Right Caudal Middle Frontal 1 |  |  | Right Lateral Occipital 1 |
|  | Right Caudal Middle Frontal 2 |  |  | Right Lateral Occipital 2 |
| 8 | Right Accumbens Area 1 |  |  | Right Inferior Parietal 1 |
|  | Right Pallidum 1 |  |  | Right Inferior Parietal 2 |
|  | Right Pallidum 2 |  |  | Right Inferior Parietal 3 |
|  | Right Putamen 1 |  |  | Right Cuneus 1 |
|  | Right Putamen 2 |  |  | Right Cuneus 2 |
|  | Right Caudate 1 |  | 8 | Right Amygdala 1 |
|  | Right Caudate 2 |  |  | Right Amygdala 2 |
|  | Right Caudate 3 |  |  | Right Amygdala 3 |
|  | Right Caudate 4 |  |  | Right Amygdala 4 |
|  | Right Caudate 5 |  |  | Right Amygdala 5 |
|  | Right Caudate 6 |  |  | Right Amygdala 6 |
|  | Right Thalamus Proper 1 |  |  | Right Hippocampus 1 |
|  | Right Thalamus Proper 2 |  |  | Right Hippocampus 2 |
|  | Right Superior Frontal 1 |  |  | Right Superior Temporal 1 |
|  | Right Superior Frontal 2 |  |  | Right Parahippocampal 1 |
|  | Right Superior Frontal 3 |  |  | Right Parahippocampal 2 |
|  | Right Rostral Anterior Cingulate 1 |  |  | Right Inferior Temporal 1 |
|  | Right Rostral Anterior Cingulate 2 |  |  | Right Fusiform 1 |
|  | Right Medial Orbitofrontal 1 |  |  | Right Fusiform 2 |
|  | Right Medial Orbitofrontal 2 |  |  | Right Entorhinal 1 |
|  | Right Lateral Orbitofrontal 1 |  |  | Right Entorhinal 2 |
|  | Right Lateral Orbitofrontal 2 |  |  | Right Entorhinal 3 |
|  | Right Caudal Anterior Cingulate 1 |  |  | Right Entorhinal 4 |
|  | Right Caudal Anterior Cingulate 2 |  |  | Right Entorhinal 5 |

**References**

Achard, S., & Bullmore, E. (2007). Efficiency and cost of economical brain functional networks. *PLoS Computational Biology*, *3*(2), 174–183. https://doi.org/10.1371/journal.pcbi.0030017

Blondel, V. D., Guillaume, J.-L., Lambiotte, R., & Lefebvre, E. (2008). Fast unfolding of communities in large networks. *Journal of Statistical Mechanics: Theory and Experiment*, *2008*(10), P10008. https://doi.org/10.1088/1742-5468/2008/10/p10008

Fornito, A., Zalesky, A., & Bullmore, E. T. (2016). *Fundamentals of Brain Network Analysis*. Academic Press.

Good, B. H., de Montjoye, Y.-A., & Clauset, A. (2010). Performance of modularity maximization in practical contexts. *Phys. Rev. E*, *81*(4), 046106. https://doi.org/10.1103/PhysRevE.81.046106

Lo, C.-Y., Wang, P.-N., Chou, K.-H., Wang, J., He, Y., & Lin, C.-P. (2010). Diffusion Tensor Tractography Reveals Abnormal Topological Organization in Structural Cortical Networks in Alzheimer’s Disease. *Journal of Neuroscience*, *30*(50), 16876–16885. https://doi.org/10.1523/JNEUROSCI.4136-10.2010
